# Supplementary material for: MOBFinder: a tool for mobilization typing of plasmid metagenomic fragments based on a language model
Source: Gigascience. 2024 Aug 5;13:giae047. doi: 10.1093/gigascience/giae047 (PMC11299106; doi:10.1093/gigascience/giae047)
Supplement: giae047_GIGA-D-24-00070_Original_Submission [file giae047_giga-d-24-00070_original_submission.pdf]

GigaScience

MOBFinder: a tool for MOB typing for plasmid metagenomic fragments based on language model

--Manuscript Draft--

Manuscript Number:	GIGA-D-24-00070
Full Title:	MOBFinder: a tool for MOB typing for plasmid metagenomic fragments based on language model
Article Type:	Technical Note
Funding Information:	National Natural Science Foundation of China (82102508) — Dr. Zhencheng Fang<br>National Natural Science Foundation of China (81925026) — Prof. Hongwei Zhou<br>National Key Research and Development Program of China (2022YFA0806400) — Prof. Hongwei Zhou
Abstract:	Background<br>MOB typing is a classification scheme that classifies plasmid genomes based on their relaxase gene. The host range of plasmids of different MOB categories are diverse and MOB typing is crucial for investigating the mobilization of plasmid, especially the transmission of resistance genes and virulence factors. However, MOB typing of plasmid metagenomic data is challenging due to the highly fragmented characteristic of metagenomic contigs.<br>Results<br>We developed MOBFinder, an 11-class classifier to classify the plasmid fragments into 10 MOB categories and a non-mobilizable category. We first performed the MOB typing for classifying complete plasmid genomes using the relaxase information, and constructed the artificial benchmark plasmid metagenomic fragments from these complete plasmid genomes whose MOB types are well annotated. Based on natural language models, we used the word vector to characterize the plasmid fragments. Several random forest classification models were trained and integrated for predicting plasmid fragments with different lengths. Evaluating the tool over the benchmark dataset, MOBFinder demonstrates higher performance compared to the existing tools MOBscan and MOB-suite, with an overall accuracy of approximately 59% higher than the MOB-suite. Moreover, the balanced accuracy, harmonic mean and F1-score could reach 99% in some MOB types. In an application focused on a T2D cohort, MOBFinder offered insights suggesting that the MOBF type plasmid, which is widely present in Escherichia and Klebsiella, and MOBQ type plasmid, might accelerate the antibiotic resistance transmission in patients suffering from T2D.<br>Conclusions<br>To the best of our knowledge, MOBFinder is the first tool for MOB tying for plasmid metagenomic fragments. MOBFinder is freely available at https://github.com/FengTaoSMU/MOBFinder.
Corresponding Author:	Zhencheng Fang<br>Zhujiang Hospital of Southern Medical University<br>Guangzhou, CHINA
Corresponding Author Secondary Information:	
Corresponding Author's Institution:	Zhujiang Hospital of Southern Medical University
Corresponding Author's Secondary Institution:	
First Author:	Tao Feng
First Author Secondary Information:	
Order of Authors:	Tao Feng

Powered by Editorial Manager® and ProduXion Manager® from Aries Systems Corporation

	Shufang Wu
	Hongwei Zhou
	Zhencheng Fang
**Order of Authors Secondary Information:**	
**Additional Information:**	
**Question**	**Response**
Are you submitting this manuscript to a special series or article collection?	No
**Experimental design and statistics**<br><br>Full details of the experimental design and statistical methods used should be given in the Methods section, as detailed in our Minimum Standards Reporting Checklist. Information essential to interpreting the data presented should be made available in the figure legends.<br><br>Have you included all the information requested in your manuscript?	Yes
**Resources**<br><br>A description of all resources used, including antibodies, cell lines, animals and software tools, with enough information to allow them to be uniquely identified, should be included in the Methods section. Authors are strongly encouraged to cite Research Resource Identifiers (RRIDs) for antibodies, model organisms and tools, where possible.<br><br>Have you included the information requested as detailed in our Minimum Standards Reporting Checklist?	Yes
**Availability of data and materials**<br><br>All datasets and code on which the conclusions of the paper rely must be either included in your submission or deposited in publicly available repositories (where available and ethically	Yes

appropriate), referencing such data using a unique identifier in the references and in the "Availability of Data and Materials" section of your manuscript.<br><br>Have you have met the above requirement as detailed in our Minimum Standards Reporting Checklist?	

Powered by Editorial Manager® and ProduXion Manager® from Aries Systems Corporation

Manuscript
Click here to access/download;Manuscript;manuscript.docx

MOBFinder: a tool for MOB typing for plasmid metagenomic fragments based on language model

Tao Feng[a], Shufang Wu[a], Hongwei Zhou[a, *] and Zhencheng Fang[a, *]

a Microbiome Medicine Center, Department of Laboratory Medicine, Zhujiang Hospital, Southern Medical University, Guangzhou 510280, China

*To whom correspondence should be addressed.

Institutional addresses: 253 Gongye Middle Avenue, Haizhu District, Guangzhou, Guangdong, China, 510280.

Email addresses:
Tao Feng: fengtaosmu@foxmail.com
Shufang Wu: wu-shufang@pku.edu.cn
Hongwei Zhou: hzhou@smu.edu.cn
Zhencheng Fang: fangzc@smu.edu.cn

// # Abstract

Background

MOB typing is a classification scheme that classifies plasmid genomes based on their relaxase gene. The host range of plasmids of different MOB categories are diverse and MOB typing is crucial for investigating the mobilization of plasmid, especially the transmission of resistance genes and virulence factors. However, MOB typing of plasmid metagenomic data is challenging due to the highly fragmented characteristic of metagenomic contigs.

Results

We developed MOBFinder, an 11-class classifier to classify the plasmid fragments into 10 MOB categories and a non-mobilizable category. We first performed the MOB typing for classifying complete plasmid genomes using the relaxase information, and constructed the artificial benchmark plasmid metagenomic fragments from these complete plasmid genomes whose MOB types are well annotated. Based on natural language models, we used the word vector to characterize the plasmid fragments. Several random forest classification models were trained and integrated for predicting plasmid fragments with different lengths. Evaluating the tool over the benchmark dataset, MOBFinder demonstrates higher performance compared to the existing tools MOBscan and MOB-suite, with an overall *accuracy* of approximately 59% higher than the MOB-suite. Moreover, the *balanced accuracy*, *harmonic mean* and *F1-score* could reach 99% in some MOB types. In an application focused on a T2D cohort, MOBFinder offered insights suggesting that the MOBF type plasmid, which is widely present in *Escherichia* and *Klebsiella*, and MOBQ type plasmid, might accelerate the antibiotic resistance transmission in patients suffering from T2D.

Conclusions

To the best of our knowledge, MOBFinder is the first tool for MOB tying for plasmid metagenomic fragments. MOBFinder is freely available at https://github.com/FengTaoSMU/MOBFinder.

Keywords: MOB typing; language model; metagenomic sequencing; plasmid; random forest

1. Introduction

Plasmids are usually small, double-stranded, and circular DNA molecules found within bacterial

cells [1]. Existing separately from the bacterial chromosome, plasmids have the ability to replicate independently and can transition between bacteria through conjugation [2]. Bacteria, specifically pathogenic strains, can acquire antibiotic resistant genes or virulence factors via plasmid-mediated horizontal gene transfer, thereby equipping them to adapt to various environments [3].

Plasmid classification is important for investigating multiple properties of plasmids, such as host range, replication and mobilization mechanism [4]. Many classification schemes have been developed according to the distinct characteristics of plasmids, like taxonomic classification, replicon (Rep) typing, incompatibility (Inc) typing, mate-pair formation (MPF) typing and mobilization (MOB) typing. Taxonomic classification refers to classify plasmids based on their host bacteria [5]. Rep typing is accomplished by genes controlling plasmid replication, known as replication initiation (Rep) protein [4, 6]. As plasmids with similar replication or partition system are incompatible within the same cell, Inc typing is a method of categorizing plasmids based on their compatibility [6]. MPF typing is based on the discovery of genes encoding the MPF system, which consists of proteins that can mediate contact and DNA exchange between donor and recipient cells during conjugation [4, 7]. Compared to these methods, MOB typing, another classification scheme, classifies plasmids based on the relaxase gene, which is present in all transmissible plasmids [8-10]. Plasmids with different MOB types, classified according to their relaxase types, possess distinct transmission mechanisms that determine their taxonomic host range [4, 11]. These variations among different MOB types are critical in researching the spread of virulence traits, the emergence of antibiotic resistance, and the adaption and evolution of bacteria. Moreover, MOB typing has demonstrated its effectiveness in identifying novel mobilizable plasmids that were previously unassigned to any Rep or Inc types, and investigating the mobilization characteristics of plasmids that have similar mobilization systems [12, 13].

Recently, many experimental and computational schemes have been devised for plasmid typing, as well as to explore the diversity and functionality of plasmids (Table 1). PlasTax-PCR (PLASmid TAXonomic PCR) [14], PBRT (PCR-Based Replicon Typing) [15], and DPMT (Degenerate Primer MOB Typing) [12] are multiplex PCR methods devised to identify plasmids with analogous replication or mobilization systems. PlasTrans, designed based on deep learning, was available to

identify mobilizable metagenomic plasmid fragments [16]. Web servers like PlasmidFinder [6], pMLST, and oriTfinder [17] were established utilizing collected maker gene databases and alignment-based methods, to facilitate Rep typing, Inc typing, or MOB typing. COPLA [5], developed based on average nucleotide identity, is a tool designed to perform taxonomic classifications of complete plasmid genomes with an overall accuracy of 41%. For the MOB typing, MOBscan [18] uses the HMMER model to annotated the relaxases and further perform MOB typing. Another tool, MOB-suite [19, 20], was designed to perform plasmid typing for plasmid assemblies. MOB-suite using Mash distance to cluster plasmid assemblies into clusters, and then using collected marker gene databases to annotate them with an *e-value* of 1e-5, a *query coverage* of 80% and an *identity* of 80%.

Table 1. Experimental and computational schemes developed for plasmid classification.

Metagenomic sequencing makes it possible to obtain all plasmid DNA from microbial communities at once, and a number of computational tools for identify plasmid fragments from metagenomic data have been developed, such as PlasFlow [21], PlasmidSeeker [22], PlasClass [23], PPR-Meta [24] and PlasForest [25]. As the DNA fragments of plasmids and bacteria are intermingled in metagenomic data [26], recognizing the host and transmission range of plasmids can be challenging. To explore the host range and transmission mechanism of plasmids with different mobilizable systems using metagenomic sequencing, it is crucial to achieve the MOB annotation of metagenomic plasmid fragments. However, obstacles arise due to the incompleteness of plasmid assembly fragments from metagenomic data and the absence of essential genes for annotation, thereby making it difficult to accurately annotate the MOB class of plasmid fragments. Given that plasmids with the same MOB type share similarities in their transmission mechanisms and host ranges, the genomic signatures, such as the GC content and codon usage of each MOB type tend to be alike, not only relaxase [4, 27]. Neural networks have demonstrated powerful performance in the classification and identification of biological sequences [28, 29]. Furthermore, language models [30, 31] derived from these neural networks have also showcased their impressive ability to characterize sequence features [32, 33]. In this methodology, short sequences of nucleotides (referred to as *k*-mers) or amino acids are analogous to "words", and the longer sequences of DNA or proteins are

analogous to “sentences”. Through the application of unsupervised learning on large datasets, each “word” is linked to a feature vector that captures its context, offering a more sophisticated analysis than the traditional *k*-mer frequency method, which simply counts the occurrence of nucleotide sequences without acknowledging their biochemical characteristics. Unlike the conventional method, this language model-based approach assesses sequences based on their contextual importance across different genetic environments, positioning contextually similar sequences close together in a multidimensional space. This technique provides deeper insights into the biochemical complexities of nucleotide sequences, thereby furnishing a more comprehensive understanding of an organism’s functional biology [34]. To characterize the features of plasmids within the same MOB type, we employed language models to perform the MOB annotation. In addition to the relaxase-coding gene, language models exhibit the ability to capture more biological features and associations within comparable mobilization systems, making it possible to perform MOB annotation for metagenomic plasmid assemblies.

To address the challenge of MOB typing for metagenomic plasmid fragments, we presented MOBFinder, a tool designed for annotating MOB types in plasmid metagenomic fragments. MOBFinder can process single or multiple plasmid DNA sequences, and provide predicted MOB types for each input fragment, including MOBB, MOBC, MOBF, MOBH, MOBL, MOBM, MOBP, MOBQ, MOBT, MOBV and non-mob. Moreover, MOBFinder also provides the option to annotate plasmid bins from metagenomics data. The overview of this work is shown in Figure 1A, and the development of MOBFinder involved the following steps: (1) Benchmark dataset construction: Plasmid complete genomes obtained from National Center for Biotechnology Information (NCBI) were classified into different MOB types based on relaxase databases. To simulate plasmid fragments in metagenomic data, artificial benchmark datasets with varying lengths were generated. (2) Word embeddings. Numerical word vectors were generated using skip-gram to characterize the sequence features in different MOB categories. (3) Classification model ensemble and optimization. Several classification models, specifically designed for different lengths, were trained and then integrated to enhance the overall performance of MOBFinder. The evaluation of the test dataset demonstrated that MOBFinder is a powerful tool for MOB typing of plasmid fragments and bins. MOBFinder’s application in a T2D cohort revealed a potential correlation between some MOB

types and the spread of antibiotic resistance genes among T2D patients. This indicates that MOBFinder offers an effective data analysis approach for investigating plasmid-mediated horizontal gene transfer within microbial communities.

2. Materials and methods

2.1. The workflow of MOBFinder

To annotate the MOB type of plasmid fragments in metagenomics, we designed MOBFinder (Figure 1). As MOB-suite [19, 20] didn't offer a quantitative likelihood score for the outcomes and some plasmids would be classified into multiple MOB types (Figure S1), we constructed a benchmark dataset using a high-resolution MOB typing strategy for categorizing complete plasmid genomes (Figure 1B, 1C). Then, based on a language model and random forest, we designed an algorithm to perform MOB typing for plasmid metagenomic fragments (Figure 1D, 1E).

Figure 1. The overview of the technical approach utilized in this study. (A). The workflow for the development and testing of the MOBFinder tool. (B). Using plasmid relaxases with known MOB types as reference sequences, we developed a database of relaxases from the NR database representing different MOB types. (C). Utilizing the relaxase database constructed in (B), complete plasmid genomes from the NCBI were subjected to MOB typing. (D). Based on the plasmid complete genome data in NCBI, we trained a 4-mer language model using the skip-gram algorithm, allowing each 4-mer to be represented by a 100-dimensional word vector. For a DNA fragment, the average word vector of all 4-mers on its sequence serves as the feature vector for that DNA. (E). We constructed simulated metagenomic contigs from the plasmid complete genomes that had been MOB typed in (C) as a benchmark and encoded these contigs into word vectors. These word vectors were then used to train a random forest. The trained random forest, with metagenomic DNA fragments as input, can predict the MOB typing of the corresponding DNA fragment based on its word vectors.

2.2. MOB typing for complete plasmid genomes

Traditionally, plasmid MOB typing of complete plasmid genomes has been a bioinformatics task

based on the analysis of relaxase sequence similarity. The practice of annotating MOB types through BLAST similarity searches using representative sequences of different MOB type relaxases has gradually evolved into the standard method for MOB typing [4, 19, 20]. In this work, we aim to construct simulated metagenomic benchmark contigs using plasmid complete genome data with known MOB typing, and published works on plasmid complete genome MOB typings have included a relatively small number of plasmids in their analyses. To expand the MOB typing dataset for plasmid complete genomes, we annotated the newly collected plasmid complete genome data for MOB typing, utilizing relaxase information.

Ten validated MOB relaxase protein families were collected, including MOBB, MOBC, MOBF, MOBH, MOBL, MOBM, MOBP, MOBQ, MOBT and MOBV [7-10, 35, 36] (Figure 1B). For each MOB category, blastp [37] was used to search homologous protein sequences against NCBI non-redundant protein sequence database, with an *e-value* threshold of 1e-10, a *query coverage* threshold of 70% and an *identity* threshold of 70%. In previous study, the selection criteria for homologous protein sequence searches are established with an *e-value* threshold of 1e-5, and minimum requirements for *query coverage* and *identity* set at 50% [4]. However, employing these criteria, we observed that certain relaxases could be annotated as belonging to multiple MOB types. To eliminate ambiguous annotations and construct a more reliable dataset for the training of MOBFinder, we imposed stricter criteria for the homologous sequence search of relaxases, setting the *e-value* threshold to 1e-10, and raising both identity and query coverage to 70%. After the expansion of protein sequences, local relaxase databases were built using the ‘makeblastdb’ command for MOB typing of plasmid genomes.

Plasmid genomes were retrieved from the NCBI nucleotide database using the keywords ‘complete’ and ‘plasmid’, and incomplete plasmid fragments were removed manually for further analysis. The accession list of these plasmids is provided in Supplementary Table 1. For each plasmid genome, coding sequences were extracted from the genebank file, and blastp [37] was employed to search for the best alignment of local relaxase databases. Here, we defined the *mob_score* to measure the likelihood of homology:

$$mob\_score = \sqrt{0.01 * qcov\_max * (1 - 1/\log10(bitscore\_max))}$$

where *qcov_max* and *bitscore_max* represent the *query coverage* and *bitscore* corresponding to the match with highest bit score, respectively. To identify plasmid genomes encoding known relaxase families, we set a *mob_score* threshold of 0.5, which was established in conjunction with a minimum *query coverage* of 50 and a minimum *bitscore* of 100. To further enhance the reliability of our classification, we introduced an *e-value* cutoff, conservatively set at 1e-10, to facilitate the plasmid genome classification (Figure 1C). In instances where plasmid genomes yielded no blast results or exhibited an *e-value* exceeding 0.01, we categorized them as non-mob.

2.3. Word embeddings using a language model

To characterize the features and patterns within each MOB category and use numerical word vectors to represent them, we utilized a skip-gram language model [30, 31] to learn from plasmid genomes. Using a fixed-size sliding window, the skip-gram algorithm calculated the likelihood between segmented words and outputted a probability distribution over the context words. The training steps were as follows (Figure 1D):

(1) Word generation. Because the plasmid DNA sequences are composed of four nucleotide bases: ‘A’, ‘T’, ‘C’, and ‘G’, we used a 4-mer sliding window to generate overlapping input words. For example, ‘ATCGCTGA’ would be segmented into ‘ATCG’, ‘TCGC’, ‘CGCT’, ‘GCTG’ and ‘CTGA’. In this step, 256 unique words will be generated.

(2) Word encoding initialization. Each word is initially assigned a random vector.

(3) Skip-gram model. We employ a standard skip-gram model as described in [30, 31] for the word vector generation through the dna2vec module [31]. A two-layer neural network was used to construct the skip-gram model. The input is the initialized vectors, and the output is a probability distribution over the input words. Layer 1 is a hidden layer to convert the input initialized vectors into a 100-dimensional word vector representation as predefined by Ng [31]. Layer 2 is used to compute and maximize the probability of the correct context words using the negative sampling function, and the size of context words was set to 20 (10 words for upstream and downstream

respectively) as pre-set by Ng [31].

(4) Model training. For each input plasmid genome, we used an optimization algorithm to minimize the loss function. Using the default setting, we then used backpropagation to update the neural network parameters (word vectors) for 10 epochs.

(5) Word vector extraction. After the training process, the word vectors in the hidden layer would be extracted to characterize the plasmid fragments in metagenomics.

2.4. Benchmark datasets construction

Because there is no real metagenomic data to serve as a benchmark, using simulated data as benchmark dataset is the common approach for bioinformatics tools development [16, 24]. In the development of MOBFinder, we artificially generated simulated datasets with the following steps:

(1) For classified plasmid genomes in each MOB category, we randomly split them with a proportion of 70% and 30% to construct the training and test datasets.

(2) Training dataset. To predict plasmid fragments with different lengths, we generated contigs with different length ranges: 100-400 bp, 401-800 bp, 801-1,200 bp and 1,201-1,600 bp. For each MOB class in four length ranges, we randomly generated 90,000 artificial contigs. Plasmid fragments longer than 1600 bp would be segmented into shorter contigs and predicted using models designed for the corresponding lengths.

(3) Test dataset. Because the plasmid fragments in real metagenomics were much longer, we generated other four length groups to assess the performance of MOBFinder: Group A with a length range of 801-1,200 bp, Group B with a length range of 1201-1,600 bp, Group C with a length range of 3,000-4,000 bp and Group D with a length range of 5,000-10,000 bp. For each MOB class in four groups, 500 fragments were randomly extracted.

2.5. Classification algorithm design

To efficiently handle the training dataset and improve the robustness of MOBFinder, we employed random forest to train four predictive models using the training dataset. The detailed steps are as follows (Figure 1E):

(1) Word representations' calculation. For each contig in the training dataset, we used a 4-mer sliding window to generate overlapping words and transformed them into numerical word vectors using trained word embeddings. To characterize underlying features and patterns of the input contigs, we summed up all the word vectors to compute their average as input of random forest.

(2) Classification models' training. To improve the performance of MOBFinder, we trained four classification models for different lengths in the training dataset: 100-400 bp, 401-800 bp, 801-1,200 bp and 1,201-1,600 bp, and the number of trees was set to 500 to generate predictive models.

(3) Model ensemble. Four trained models for different lengths were ensembled into MOBFinder to make more accurate predictions. For fragments shorter than 100 bp, we used a model designed for 100-400 bp to predict the MOB type. For fragments longer than 1,600 bp, we segmented them into short contigs according to the length of trained models and make predictions using the corresponding model. For example, a fragment with a length of 4,000 bp would be segmented into two contigs with a length of 1,600 bp and one contig with a length of 800 bp. After predicting with the corresponding models, we aggregated and calculated the weighted average scores for each MOB class, and the MOB type with the highest score will be selected as the predicted result for the input fragment.

(4) Plasmid bins' classification. Metagenomic binning is an essential step for the reconstruction of genomes from individual microorganisms. Thus, MOBFinder can perform MOB typing on both plasmid contigs and plasmid bins. If the input is a plasmid bin, MOBFinder predicted the likelihood of each MOB class for fragments within the bin. For each MOB category, MOBFinder aggregated the scores of each sequence within the bin, and calculated the weighted average scores based on the sequence length. The MOB category with the maximum score is selected as the prediction result.

2.6. Performance validation.

Test dataset was used to assess the performance of MOBFinder, and compared to MOB-suite and MOBscan. Since MOBscan can only predict the MOB type using plasmid protein sequences rather than DNA sequences, we first annotated the proteins in the plasmid fragments of the test set using Prokka [38] and then used MOBscan to predict the MOB type based on the annotated proteins. We calculated overall *accuracy*, *kappa* and *run time* by comparing the predicted classes and true classes. Given that MOBScan operates as an online tool and cannot be executed locally, the calculation of MOBScan's *run time* was confined to the duration spent on preprocessing with Prokka locally. The overall *accuracy* was the proportion of accurate predictions. The *kappa* (a) was calculated to assess the overall consistency between the predictions and true classes, which took into account the possibility of random prediction. *Po* represents observed accuracy [$Po = (A_{11} + A_{22} + ... + Ann) / N$], where A_{11}, A_{22} and *Ann* represent the values on the diagonal of the confusion matrix, and *n* represents the number of MOB categories, while *N* represents the total number of samples. *Pe* represents expected accuracy [$Pe = (E_{11} + E_{22} + ... + Enn) / N^2$], where E_{11}, E_{22} and *Enn* represent the expected values in each cell of the confusion matrix, *n* represents the number of MOB classes, and *N* represents the total number of samples. The *run time* was recorded using the command 'time' in Linux.

$$kappa = (Po - Pe)/(1 - Pe) \qquad (a)$$

$$balanced\ accuracy = (TPR + TNR)/2 \qquad (b)$$

$$harmonic\ mean = 2 * Sn * Sp/(Sn + Sp) \qquad (c)$$

$$F1 - score = 2 * precision * recall/(precision + recall) \qquad (d)$$

For each MOB category, we also calculated the *balanced accuracy* (b), *harmonic mean* (c) and *F1-score* (d). Considering the class imbalance within the training dataset, *balanced accuracy* was used to measure the average accuracy of each MOB category, where *TPR* is the true positive rate [*TRP* = true positives / (true positives + false negatives)], and *TNR* is the true negative rate [*TNR* = true negatives / (true negatives + false positives)]. *Harmonic mean* could provide an overall evaluation of a model's performance, where *Sn* and *Sp* represent sensitivity [*Sn* = true positives/ (true positives + false negatives)] and specificity [*Sp* = true negatives / (true negatives + false positives)]. *F1-score* combines *precision* and *recall*, providing a balanced measure of a model's performance, where *precision* was the correct positive prediction in all positive predictions [*precision* = true positives /

(true positives + false positives)] and *recall* was the correct positive predictions in all actual positives [*recall* = true positives / (true positives + false negatives]].

The receiver operating characteristic (ROC) curve was used to visualize the performance of MOBFinder in predicting each MOB category, where the x-axis and y-axis were false positive rate (*FPR*) and true positive rate (*TPR*). The ROC curve that is closer to the left and top indicates a higher *TPR* and lower *FPR*, which means better performance. For each MOB class, the area under the curve (AUC) value was calculated to quantify the performance of MOBFinder. The AUC value between 0.5 and 1 indicates the model performs better than random chance, and a higher AUC value indicates better prediction capability.

2.7. Annotation and analysis of T2D metagenomic data

Metagenomic sequencing data (SRA045646) were retrieved from NCBI short read archive (SRA) database to investigate whether the plasmids within different MOB classes were associated with the resistance enrichment in T2D patients, as suggested by the previous studies [39, 40]. All metagenomic data were preprocessed using the same protocols. PRINSEQ [41] was used to remove low-quality reads and bowtie2 [42] to remove host reads by aligning them to the human GRCH38 reference genome downloaded from ENSEMBL database. We excluded metagenomic samples that did not pass the quality control. Because the plasmid abundance in metagenomes was much lower than bacteria, we only retained samples with more than 10,000,000 paired-end reads for downstream analysis (Supplementary Table 2).

To improve the efficiency and accuracy of assembly, we used MEGAHIT [43] to generate metagenomic contigs. PPR-Meta [24] was utilized to identify and extract plasmid fragments from the assembled fragments, while filtering out bacterial and phage sequences. COCACOLA [44] was employed to cluster plasmid fragments into bins based on their sequence similarity and composition. This could help us to investigate the plasmid fragments from same originate and enable a better annotation and anlysis of their functions.

MOBFinder was applied to annotate the MOB types for each plasmid bin. The average fragments

per kilobase per million (FPKM) of each plasmid bin was calculated using bowtie2 to represent its abundance. We conducted an analysis on the significance of differences of plasmid bins and various MOB types between healthy and T2D groups using the unpaired Wilcoxon signed-rank two-sided test. The calculation of *p* values was adjusted for multiple comparisons using the Benjamini-Hochberg (BH) method (denoted as *p.adjust*). ABRicate [45] was utilized to annotate the antibiotic resistance genes (*identity*>50% and *qcov*>50%) in each plasmid bin, based on four antibiotic resistance gene databases [46-49]. The Tukey's Honest Significant Difference test was performed to compare the identified resistant genes for different MOB classes. All statistical analysis were achieved using R.

3. Results

3.1. MOB typing for plasmid genomes

To construct the benchmark datasets, we obtained 90,395 complete plasmid genomes and categorized them into 11 MOB categories using blast (Table 2). We removed 22,470 plasmid genomes potentially classified into more than one MOB class, leaving 67,925 classified genomes for the training and optimization of MOBFinder (Figure 2A). Our analysis results revealed significant variations among the plasmid genomes of different MOB types, including the number, average length, and GC content. Notably, non-MOB type plasmid genomes held the highest count and longest average length, whereas MOBB and MOBM had the fewest plasmid genomes and shortest average length, respectively. In terms of GC content, MOBL and MOBQ represent the lowest and highest MOB type. Moreover, plasmids of different MOB types exhibited diverse host ranges in genus level (Figure 2B). MOBB was predominantly found in *Bacteroides*, *Hymenobacter*, *Parabacteroides*, *Phocaeicola* and *Spirosoma*. Particularly, *Phocaeicola* has been detected in the human gut and possessed the gene for porphyran degradation through horizontal gene transfer [50]. MOBC, MOBF, MOBH and MOBP all existed in *Escherichia* and *Klebsiella*. Furthermore, *Klebsiella* is a multidrug-resistant bacterium that has demonstrated resistance to multiple antibiotics [51]. MOBL, MOBT and MOBV were mainly discovered in *Bacillus* and *Enterococcus*. Additionally, almost all MOBM type plasmid genomes were present in *Clostridium* and *Enterocloster*, and some species in *Clostridium* could cause various diseases [52]. MOBQ

demonstrated a broader host range, including *Acinetobacter*, *Agrobacterium*, *Escherichia*, *Rhizobium*, *Lactiplantibacillus* and *Staphylococcus*. Non-mobilizable plasmids were detected in the majority of bacteria. These results illustrated the relationship between different MOB types and their host range, and it demonstrated that MOB typing for plasmid fragments is feasible in the absence of relaxases.

Table 2. The number, average length, and GC content of plasmid genomes for each MOB type.

Figure 2. Benchmark dataset construction using a high-resolution strategy. (A). The proportion of classified plasmid genomes. The confidence is ‘sure’ means the classified plasmid genomes had a *mob-score* more than 0.5 and an *e-value* less than 1e-10, while ‘possible’ had not. Plasmid genomes identified as ‘sure’ were used to generate benchmark datasets. Non-mob represents non-mobilizable plasmid. (B). The host range of classified plasmid genomes in the genus level. Different colors represent different genera, and genera accounting for less than 5% of the total abundance are grouped under the category ‘other’.

3.2. Overall performance of MOBFinder

We used the *accuracy*, *kappa* and *run time* to evaluate the overall performance of MOBFinder. In the comparison, it was observed that MOBscan did not perform well, achieving low *accuracy* and *kappa* values across sequences of varying lengths, while MOB-suite exhibited marginally better performance than MOBscan when handling sequences of greater length (Figure 3A, 3B). Compared to MOB-suite, the *accuracy* of MOBFinder ranged from 70% to 77%, which exhibited a significant improvement of at least 59% (Figure 3A). The *kappa* of MOBFinder ranged between 67% and 75% and was approximately 65% higher than MOB-suite (Figure 3B). Moreover, MOBFinder exhibited a significantly shorter *run time* in the test dataset, with a more gradual increase trend (Figure 3C). In general, MOBFinder demonstrated a consistent performance improvement as the sequence length increased.

Figure 3. Overall performance of MOBFinder and comparison to MOB-suite and MOBScan. (A-C) The performance evaluation and comparison between MOBFinder, MOB-suite and MOBscan

using *accuracy* (A), *kappa* (B) and *run time* (C). In test datasets, four length groups were generated: Group A: 801-1200 bp, Group B: 1201-1600 bp, Group C: 3000-4000 bp and Group D: 500-10000 bp. (D) For each MOB type, the *balanced accuracy*, *harmonic mean* and *F1-score* were used to assess the performance of MOBFinder and compared to MOB-suite and MOBscan. MOBFinder, MOB-suit and MOBscan are represented by blue lines, orange lines and gray lines respectively.

3.3. Evaluation in each MOB category

To evaluate the discrimination ability of MOBFinder in each MOB type, we calculated the *balanced accuracy*, *harmonic mean* and *F1-score* using the test dataset (Figure 3D). In MOBB and MOBM, MOBFinder demonstrated the highest performance, while its ability to identify non-mob class is comparatively lower than other classes. In MOBM, the *balanced accuracy* and *harmonic mean* could reach 99%, and *F1-score* exceeded 96% in all length groups. In non-mob, the *balanced accuracy* was 65%, the *harmonic mean* was 49% and the *F1-score* was 40%. Compared to MOB-suite, MOBFinder exhibited much better performance in predicting all MOB classes. Even in non-mob, MOBFinder showed approximately 13% improvement in *balanced accuracy*, 34% in *harmonic mean* and 24% in *F1-score*.

The receiver operating characteristic (ROC) curve exhibited the performance of MOBFinder in all MOB categories, and the area under the curve (AUC) was calculated to quantify the performance (Figure 4). We found all AUCs were greater than 0.8, which indicated MOBFinder could effectively distinguish positive and negative samples in each MOB class. The AUC values were higher than 0.9 in most MOB types, while in MOBT and non-mob were less than 0.9. The performance differences in identifying each MOB type might be attributed to the varying host ranges and sequence features. Additionally, the imbalance in the training dataset for each MOB type may also be a primary factor contributing to the performance disparities.

Figure 4. The ROC curves and AUC values of MOBFinder. The ROC curves were plotted using the output scores of MOBFinder, and the AUC values were calculated to quantify the performance of MOBFinder in each MOB class.

3.4. Application in T2D metagenomic analysis

Upon analysis of fecal samples in metagenomic studies, antibiotic resistance pathways were found to be enriched in patients with T2D [40]. The precise mechanism, however, remained elusive. We applied MOBFinder to analyze real T2D metagenomic data [39]. After preprocessing and assembly, 2,217,064 metagenomic fragments were generated, and plasmid assemblies were identified using PPR-Meta. Subsequently, the plasmid fragments were clustered into 55 bins and annotated using MOBFinder. By employing MOBFinder, we assigned 2 bins to the MOBF class, 8 bins to MOBL, 17 bins to MOBQ, and identified 28 bins as non-mob (Figure 5A). Furthermore, we detected 15 bins that exhibited significant difference between the T2D group and the control group. Among them, 1 bin was classified as MOBF, 2 bins as MOBL, 5 bins as MOBQ, and 7 bins as non-mob (Figure S2). Among above MOB types, MOBQ contains the highest number of bins enriched in T2D, while MOBF is widely present in *Escherichia* and *Klebsiella* (Figure 2B), and some strains in *Klebsiella* is resistant to multiple antibiotics, including carbapenems [53], indicating that these two kinds of MOB types' plasmid might contribute to the antibiotic resistance enrichment in T2D.

Figure 5. The annotation of T2D-related plasmid bins using MOBFinder. (A). Heatmap of plasmid bins between T2D and control. Each column represents a sample, and each row represents a plasmid bin. For each column, the color blue represents the T2D group, and the color yellow represents the control group. The four distinct colors on the y-axis represent identified four different MOB types using MOBFinder. (B). The abundance comparison the four identified MOB types between T2D and control. The *p-value* was calculated using Wilcoxon signed-rank two-sided test, adjusted by "BH" method for multiple comparisons. ("*" represents *p.adjust* < 0.01, "**" represents *p.adjust* < 0.05 and "***" represents *p.adjust* < 0.001.)

We compared the average abundance between the T2D group and the control group for each MOB type (Figure 5B). We indeed observed a significant increase in the abundance of MOBF and MOBQ within the T2D group, and these two types of MOB plasmids can be transferred among multiple bacterial species. This suggested that the increase of MOBF-type and MOBQ-type plasmids could potentially raise the risk of infection among individuals with T2D. Subsequently, we used four databases [46-49] to detect drug resistance genes in four MOB types (Figure 6). Among them, the

number of identified drug resistance genes in the MOBF-type plasmids was significantly higher than the other three MOB types. This indicates that MOBF-type plasmids could carry much more drug resistance genes compared to other MOB classes. These findings suggested that the increase of MOBF type and MOBQ type plasmids could result in more bacteria acquiring drug resistance genes, thereby leading to the enrichment of resistance pathways in T2D. In summary, our results demonstrated the utility of MOBFinder for annotation of plasmid fragments in metagenomes, shedding light on the mechanisms fueling the emergence of antibiotic resistance enrichment in metagenomic analysis.

Figure 6. Comparison of resistant genes among different MOB types. Four databases were used to identify resistant gene within each MOB type, and the *p-value* was calculated using Tukey's Honest Significant Difference test. The two groups without annotated significant differences exhibit no statistical disparity. ("*" represents *p-value* < 0.01, "**" represents *p-value* < 0.05 and "***" represents *p-value* < 0.001.)

3.5. The usage of MOBFinder

MOBFinder can predict the MOB type of plasmid fragments and bins in metagenomics. For plasmid metagenomic fragments, MOBFinder takes a FASTA file as input. The output file consists of 13 columns. The first column represents the fragment ID, the second column displays the predicted MOB type and columns three to thirteen represent the scores for each MOB class, namely MOBB, MOBC, MOBF, MOBH, MOBL, MOBM, MOBP, MOBQ, MOBT, MOBV, and non-mob.

For plasmid metagenomic bins, MOBFinder requires two input files: a FASTA file containing the plasmid fragments and a meta table that records the mapping between plasmid fragment IDs and bin IDs. The output results are similar to the output of plasmid fragments. The first column is the plasmid bin's id. The second column is the predicted MOB class of plasmid bins. The other columns were MOB scores of different MOB categories produced by MOBFinder.

4. Discussion

In this paper, we developed MOBFinder based on a language model and the random forest. Using the relaxase-alignment method, plasmid genomes were classified into distinct MOB categories. Our analysis revealed substantial variations in parameters such as the number, average length, and GC content across various MOB types. Additionally, there are noteworthy differences in the host range among different MOB classes. These results suggested the potential of utilizing sequence features from different MOB types for plasmid metagenomic fragment MOB typing. To characterize the plasmids within each MOB type, we used skip-gram to generate word vectors. MOBFinder, integrating multiple random forest models, demonstrated superior overall performance compared to existing tools. Specifically, for each MOB category, MOBFinder exhibited significant improvements in *balanced accuracy*, *harmonic mean* and *F1-score*, with values reaching up to 99% in the MOBM category.

In the past, *k*-mer frequency models and one-hot encoding were commonly employed methods for digitizing biological sequences, extensively applied across various machine learning algorithms [54]. However, both models simply mark or count the frequency of various characters appearing in sequences, failing to profoundly reflect the biological significance underlying each character. Concurrently, these models may encounter dimensional issues [54]. For instance, in the *k*-mer model, if k is set to 8, the dimensionality of each DNA sequence's *k*-mer vector becomes 4^8, which is problematic in metagenomics where most fragment lengths do not reach this magnitude, resulting in significant noise in the feature vector and causing overfitting. Similarly, in the one-hot model, for a sequence of length L using 4-mers as the base unit, it would require L one-hot vectors each with a dimensionality of 4^4. In such instances, if the dataset for training is not sufficiently large, this representation method could also lead to overfitting due to high dimensionality. In contrast, word vector models offer a superior solution to these problems. Word vector models initially perform a random initialization of vectors for each word. Taking the skip-gram algorithm utilized in this study as an example, the dimension of this random vector can be 1-of-n, where n represents the size of the vocabulary [30]. Following unsupervised pre-training on large datasets, the algorithm maps characters with similar contexts to similar feature spaces. The dimensions of these feature spaces' coordinates (i.e., the word vectors) will be lower than those of the initial random vectors. Thus, through unsupervised pre-training driven by large datasets, language models can compress high-

dimensional initial vectors into lower-dimensional word vectors (e.g., MOBFinder's word vectors with a dimensionality of 100), enabling these feature vectors to contain more character information while effectively avoiding dimensional issues in supervised training.

In metagenomic sequences classification task, 4-mer is the most widely used as the basic unit in various bioinformatics tools [55], and MOBFinder also takes the 4-mer as a "word". To assess the impact of training word vectors with different *k*-mer lengths on performance, we compared models with *k*-mer lengths of 2, 3, 4, 5, 6, 7, and 8 (Figure S3). We observed lower overall *accuracy* and *kappa* values for *k*=2. At *k*=4, the *balanced accuracy*, *harmonic mean*, *F1-score*, and *AUC* values stabilized across different MOB types. Subsequently, as the *k*-mer length increased, there was no significant improvement in *accuracy* or other metrics, while the *run time* gradually increased. Therefore, we chose a *k*-mer length of 4 for training word vectors and developing MOBFinder.

Interestingly, through our analysis of T2D metagenomic sequencing data [39], we noted a significant increase in MOBF and MOBQ type plasmids within T2D patients. Moreover, we found that the drug resistance genes were enriched in the MOBF class, and the dominant host of MOBF class, *Klebsiella* and *Escherichia* are associated with the spread of multidrug resistance. Although previous analysis of metagenomic data from patients with T2D found the drug resistance pathways enriched [40], our analysis revealed a potential reason behind this phenomenon: the increased abundance of MOBF and MOBQ type plasmids in the gut of individuals with T2D may lead to the dissemination of more antibiotic resistance genes, resulting in the enrichment of the antibiotic resistance pathway.

At present, a large amount of human metagenomic data derived from second-generation sequencing populates various databases. However, our understanding of the functions of numerous disease-linked microbial sequences remains limited, attributed to the incomplete nature of metagenomic fragments. The development of MOBFinder enables the MOB annotation for plasmid fragments in metagenomics, and provides a powerful tool to investigate the transmission mechanisms of plasmid-mediated antibiotic resistance genes and virulence factors.

5. Conclusions

In summary, we have developed MOBFinder as a tool for MOB typing of plasmid fragments and bins in metagenomic data. Our analysis of classified plasmid genomes unveiled notable differences in sequence characteristics and host range across various MOB types. Based on this, we employed a language model to extract the sequence features specific to each MOB type and represented them using word vectors. Additionally, we boosted prediction accuracy by training and integrating several random forest classification models. MOBFinder surpassed the existing tool in the performance tests and was successful in detecting an increase in certain MOB type plasmids in T2D patients from the T2D metagenomic data analysis. Importantly, these MOB type plasmids harbor potential drug-resistant genes, thus offering an explanation for the observed antibiotic resistance in T2D individuals. This suggests MOBFinder's potential in aiding the formulation of specific medications to curb drug resistance transmission. We anticipate that MOBFinder will be a powerful tool for the analysis of plasmid-medicated transmission.

Availability of Source Code and Requirements

- Project name: MOBFinder
- Project homepage: https://github.com/FengTaoSMU/MOBFinder
- Operating system(s): Linux
- Programming language: Python, R script
- Other requirements: BLAST, biopython
- License: GPL-3.0
- RRID: SCR_024451

Availability of Supporting Data

Snapshots of our code and other data further supporting this work are openly available in the GigaScience repository, GigaDB

Abbreviations

MOB: mobilization; Rep: replicon; Inc: incompatibility; MPF: mate-pair formation; non-mob: non-mobilizable; T2D: type 2 diabetes; FPKM: fragments per kilobase per million; TPR: true positive rate; TNR: true negative rate; Sn: sensitivity; Sp: specificity; ROC: the receiver operating characteristic; AUC: the area under the curve; SRA: short read archive; NCBI: National Center for Biotechnology Information; PlasTax-PCR: PLASmid TAXonomic PCR; PBRT: PCR-Based Replicon Typing; DPMT: Degenerate Primer MOB Typing.

Competing Interests

The authors declare that they have no competing interests.

Funding

This investigation was financially supported by the National Key R&D Program of China (2022YFA0806400) and National Natural Science Foundation of China (82102508, 81925026).

Authors' Contributions

TF, ZCF and HWZ proposed and designed this work. TF and ZCF developed and optimized the software. TF, ZCF, SFW and HWZ wrote and revised the manuscript.

Supplementary data

Supplementary Material.

Supplementary Table 1. The accessions list of classified plasmid genomes.

Supplementary Table 2. The list of metagenomic samples used in our analysis.

Supplementary Figure 1. MOB typing using MOB-suite. Single-class represents plasmid genomes

classified into one MOB type, multi-class represents plasmid genomes classified into more than one MOB categories and non-mob represents non-mobilizable plasmids.

Supplementary Figure 2. The abundance of each significantly different plasmid bin from various types between the T2D and CON groups.

Supplementary Figure 3. Comparison results for developing MOBFinder using word vectors trained with different *k*-mer lengths. (A-C) Overall *accuracy*, *kappa*, and *run time* of the MOB classification model trained with word vectors trained using different lengths of k-mers. (D) *Balanced accuracy*, *harmonic mean*, *F1-score* and AUC of word vectors trained with different k-mer lengths across different MOB types.

References

1. Helinski DR. A Brief History of Plasmids. EcoSal Plus. 2022 Dec 15;10(1):eESP00282021. doi: 10.1128/ecosalplus.esp-0028-2021
2. Garcillán-Barcia MP, Francia MV, de la Cruz F. The diversity of conjugative relaxases and its application in plasmid classification. FEMS Microbiol Rev. 2009 May;33(3):657-87. doi: 10.1111/j.1574-6976.2009.00168.x
3. Rodríguez-Beltrán J, DelaFuente J, León-Sampedro R, MacLean RC, San Millán Á. Beyond horizontal gene transfer: the role of plasmids in bacterial evolution. Nat Rev Microbiol. 2021 Jun;19(6):347-359. doi: 10.1038/s41579-020-00497-1
4. Shintani M, Sanchez ZK, Kimbara K. Genomics of microbial plasmids: classification and identification based on replication and transfer systems and host taxonomy. Front Microbiol. 2015 Mar 31;6:242. doi: 10.3389/fmicb.2015.00242
5. Redondo-Salvo S, Bartomeus-Peñalver R, Vielva L, Tagg KA, et al. COPLA, a taxonomic classifier of plasmids. BMC Bioinformatics. 2021 Jul 31;22(1):390. doi: 10.1186/s12859-021-04299-x
6. Carattoli A, Hasman H. PlasmidFinder and In Silico pMLST: Identification and Typing of Plasmid Replicons in Whole-Genome Sequencing (WGS). Methods Mol Biol. 2020;2075:285-294. doi: 10.1007/978-1-4939-9877-7_20

7. Smillie C, Garcillán-Barcia MP, Francia MV, Rocha EP, de la Cruz F. Mobility of plasmids. Microbiol Mol Biol Rev. 2010 Sep;74(3):434-52. doi: 10.1128/MMBR.00020-10
8. Francia MV, Varsaki A, Garcillán-Barcia MP, Latorre A, Drainas C, de la Cruz F. A classification scheme for mobilization regions of bacterial plasmids. FEMS Microbiol Rev. 2004 Feb;28(1):79-100. doi: 10.1016/j.femsre.2003.09.001
9. Garcillán-Barcia MP, Francia MV, de la Cruz F. The diversity of conjugative relaxases and its application in plasmid classification. FEMS Microbiol Rev. 2009 May;33(3):657-87. doi: 10.1111/j.1574-6976.2009.00168.x
10. Garcillán-Barcia MP, Alvarado A, de la Cruz F. Identification of bacterial plasmids based on mobility and plasmid population biology. FEMS Microbiol Rev. 2011 Sep;35(5):936-56. doi: 10.1111/j.1574-6976.2011.00291.x
11. Bradley P, den Bakker HC, Rocha EPC, McVean G, Iqbal Z. Ultrafast search of all deposited bacterial and viral genomic data. Nat Biotechnol. 2019 Feb;37(2):152-159. doi: 10.1038/s41587-018-0010-1
12. Alvarado A, Garcillán-Barcia MP, de la Cruz F. A degenerate primer MOB typing (DPMT) method to classify gamma-proteobacterial plasmids in clinical and environmental settings. PLoS One. 2012;7(7):e40438. doi: 10.1371/journal.pone.0040438
13. Garcillán-Barcia MP, Alvarado A, de la Cruz F. Identification of bacterial plasmids based on mobility and plasmid population biology. FEMS Microbiol Rev. 2011 Sep;35(5):936-56. doi: 10.1111/j.1574-6976.2011.00291.x
14. Cuartas R, Coque TM, de la Cruz F, Garcillán-Barcia MP. PLASmid TAXonomic PCR (PlasTax-PCR), a Multiplex Relaxase MOB Typing to Assort Plasmids into Taxonomic Units. Methods Mol Biol. 2022;2392:127-142. doi: 10.1007/978-1-0716-1799-1_10
15. Carattoli A, Bertini A, Villa L, Falbo V, Hopkins KL, Threlfall EJ. Identification of plasmids by PCR-based replicon typing. J Microbiol Methods. 2005 Dec;63(3):219-28. doi: 10.1016/j.mimet.2005.03.018
16. Fang Z, Zhou H. Identification of the conjugative and mobilizable plasmid fragments in the plasmidome using sequence signatures. Microb Genom. 2020 Nov;6(11):mgen000459. doi: 10.1099/mgen.0.000459
17. Li X, Xie Y, Liu M, Tai C, Sun J, Deng Z, Ou HY. oriTfinder: a web-based tool for the

identification of origin of transfers in DNA sequences of bacterial mobile genetic elements. Nucleic Acids Res. 2018 Jul 2;46(W1):W229-W234. doi: 10.1093/nar/gky352

18. Garcillán-Barcia MP, Redondo-Salvo S, Vielva L, de la Cruz F. MOBscan: Automated Annotation of MOB Relaxases. Methods Mol Biol. 2020;2075:295-308. doi: 10.1007/978-1-4939-9877-7_21
19. Robertson J, Nash JHE. MOB-suite: software tools for clustering, reconstruction and typing of plasmids from draft assemblies. Microb Genom. 2018 Aug;4(8):e000206. doi: 10.1099/mgen.0.000206
20. Robertson J, Bessonov K, Schonfeld J, Nash JHE. Universal whole-sequence-based plasmid typing and its utility to prediction of host range and epidemiological surveillance. Microb Genom. 2020 Oct;6(10):mgen000435. doi: 10.1099/mgen.0.000435
21. Krawczyk PS, Lipinski L, Dziembowski A. PlasFlow: predicting plasmid sequences in metagenomic data using genome signatures. Nucleic Acids Res. 2018 Apr 6;46(6):e35. doi: 10.1093/nar/gkx1321
22. Roosaare M, Puustusmaa M, Möls M, Vaher M, Remm M. PlasmidSeeker: identification of known plasmids from bacterial whole genome sequencing reads. PeerJ. 2018 Apr 2;6:e4588. doi: 10.7717/peerj.4588
23. Pellow D, Mizrahi I, Shamir R. PlasClass improves plasmid sequence classification. PLOS Comput Biol. 2020;16:e1007781. doi: 10.1371/journal.pcbi.1007781
24. Fang Z, Tan J, Wu S, Li M, Xu C, Xie Z, et al. PPR-Meta: a tool for identifying phages and plasmids from metagenomic fragments using deep learning. 2019;8:1–14. 10.1093/gigascience/giz066
25. Pradier L, Tissot T, Fiston-Lavier AS, Bedhomme S. PlasForest: a homology-based random forest classifier for plasmid detection in genomic datasets. BMC Bioinformatics. 2021 Jun 26;22(1):349. doi: 10.1186/s12859-021-04270-w
26. Sobecky PA, Hazen TH. Horizontal gene transfer and mobile genetic elements in marine systems. Methods Mol Biol. 2009;532:435-53. doi: 10.1007/978-1-60327-853-9_25
27. Suzuki H, Yano H, Brown CJ, Top EM. Predicting plasmid promiscuity based on genomic signature. J Bacteriol. 2010 Nov;192(22):6045-55. doi: 10.1128/JB.00277-10
28. Wu S, Fang Z, Tan J, Li M, Wang C, Guo Q, Xu C, Jiang X, Zhu H. DeePhage: distinguishing

virulent and temperate phage-derived sequences in metavirome data with a deep learning approach. Gigascience. 2021 Sep 8;10(9):giab056. doi: 10.1093/gigascience/giab056

29. Fang Z, Feng T, Zhou H, Chen M. DeePVP: Identification and classification of phage virion proteins using deep learning. Gigascience. 2022 Aug 11;11:giac076. doi: 10.1093/gigascience/giac076

30. Mikolov T, Chen K, Corrado G, and Dean J. Efficient estimation of word representations in vector space. 2013. arXiv preprint. doi: arXiv:1301.3781.

31. Patrick Ng. dna2vec: Consistent vector representations of variable-length k-mers. arXiv. doi: 10.48550/arXiv.1701.06279

32. Tsukiyama S, Hasan MM, Fujii S, Kurata H. LSTM-PHV: prediction of human-virus protein-protein interactions by LSTM with word2vec. Brief Bioinform. 2021 Nov 5;22(6):bbab228. doi: 10.1093/bib/bbab228

33. Sharma R, Shrivastava S, Kumar Singh S, Kumar A, Saxena S, Kumar Singh R. Deep-ABPpred: identifying antibacterial peptides in protein sequences using bidirectional LSTM with word2vec. Brief Bioinform. 2021 Sep 2;22(5):bbab065. doi: 10.1093/bib/bbab065

34. Asgari E, Mofrad MR. Continuous Distributed Representation of Biological Sequences for Deep Proteomics and Genomics. PLoS One. 2015 Nov 10;10(11):e0141287. doi: 10.1371/journal.pone.0141287

35. Wisniewski JA, Traore DA, Bannam TL, Lyras D, Whisstock JC, Rood JI. TcpM: a novel relaxase that mediates transfer of large conjugative plasmids from Clostridium perfringens. Mol Microbiol. 2016 Mar;99(5):884-96. doi: 10.1111/mmi.13270

36. Ramachandran G, Miguel-Arribas A, Abia D, Singh PK, Crespo I, Gago-Córdoba C, Hao JA, Luque-Ortega JR, Alfonso C, Wu LJ, Boer DR, Meijer WJ. Discovery of a new family of relaxases in Firmicutes bacteria. PLoS Genet. 2017 Feb 16;13(2):e1006586. doi: 10.1371/journal.pgen.1006586

37. Camacho C, Coulouris G, Avagyan V, Ma N, Papadopoulos J, Bealer K, Madden TL. BLAST+: architecture and applications. BMC Bioinformatics. 2009 Dec 15;10:421. doi: 10.1186/1471-2105-10-421

38. Seemann T. Prokka: rapid prokaryotic genome annotation. Bioinformatics. 2014 Jul 15;30(14):2068-9. doi: 10.1093/bioinformatics/btu153

39. Qin J, Li Y, Cai Z, Li S, Zhu J, Zhang F et al. A metagenome-wide association study of gut microbiota in type 2 diabetes. Nature. 2012 Oct 4;490(7418):55-60. doi: 10.1038/nature11450
40. Wu H, Tremaroli V, Schmidt C, Lundqvist A, Olsson LM, Krämer M, Gummesson A, Perkins R, Bergström G, Bäckhed F. The Gut Microbiota in Prediabetes and Diabetes: A Population-Based Cross-Sectional Study. Cell Metab. 2020 Sep 1;32(3):379-390.e3. doi: 10.1016/j.cmet.2020.06.011
41. Schmieder R, Edwards R. Quality control and preprocessing of metagenomic datasets. Bioinformatics. 2011 Mar 15;27(6):863-4. doi: 10.1093/bioinformatics/btr026
42. Langmead B, Salzberg SL. Fast gapped-read alignment with Bowtie 2. Nat Methods. 2012 Mar 4;9(4):357-9. doi: 10.1038/nmeth.1923
43. Li D, Liu CM, Luo R, Sadakane K, Lam TW. MEGAHIT: an ultra-fast single-node solution for large and complex metagenomics assembly via succinct de Bruijn graph. Bioinformatics. 2015 May 15;31(10):1674-6. doi: 10.1093/bioinformatics/btv033
44. Lu YY, Chen T, Fuhrman JA, Sun F. COCACOLA: binning metagenomic contigs using sequence COmposition, read CoverAge, CO-alignment and paired-end read LinkAge. Bioinformatics. 2017 Mar 15;33(6):791-798. doi: 10.1093/bioinformatics/btw290
45. Seemann T. Abricate. Github. https://github.com/tseemann/abricate
46. Gupta SK, Padmanabhan BR, Diene SM, Lopez-Rojas R, Kempf M, Landraud L, Rolain JM. ARG-ANNOT, a new bioinformatic tool to discover antibiotic resistance genes in bacterial genomes. Antimicrob Agents Chemother. 2014;58(1):212-20. doi: 10.1128/AAC.01310-13
47. Jia B, Raphenya AR, Alcock B, Waglechner N, Guo P, Tsang KK, Lago BA, Dave BM, Pereira S, Sharma AN, Doshi S, Courtot M, Lo R, Williams LE, Frye JG, Elsayegh T, Sardar D, Westman EL, Pawlowski AC, Johnson TA, Brinkman FS, Wright GD, McArthur AG. CARD 2017: expansion and model-centric curation of the comprehensive antibiotic resistance database. Nucleic Acids Res. 2017 Jan 4;45(D1):D566-D573. doi: 10.1093/nar/gkw1004
48. Doster E, Lakin SM, Dean CJ, Wolfe C, Young JG, Boucher C, Belk KE, Noyes NR, Morley PS. MEGARes 2.0: a database for classification of antimicrobial drug, biocide and metal resistance determinants in metagenomic sequence data. Nucleic Acids Res. 2020 Jan 8;48(D1):D561-D569. doi: 10.1093/nar/gkz1010
49. Feldgarden M, Brover V, Haft DH, Prasad AB, Slotta DJ, Tolstoy I, Tyson GH, Zhao S, Hsu

CH, McDermott PF, Tadesse DA, Morales C, Simmons M, Tillman G, Wasilenko J, Folster JP, Klimke W. Validating the AMRFinder Tool and Resistance Gene Database by Using Antimicrobial Resistance Genotype-Phenotype Correlations in a Collection of Isolates. Antimicrob Agents Chemother. 2019 Oct 22;63(11):e00483-19. doi: 10.1128/AAC.00483-19

50. Hehemann JH, Correc G, Barbeyron T, Helbert W, Czjzek M, Michel G. Transfer of carbohydrate-active enzymes from marine bacteria to Japanese gut microbiota. Nature. 2010 Apr 8;464(7290):908-12. doi: 10.1038/nature08937

51. Fu S, Wang R, Xu Z, Zhou H, Qiu Z, Shen L and Yang Q. Metagenomic sequencing combined with flow cytometry facilitated a novel microbial risk assessment framework for bacterial pathogens in municipal wastewater without cultivation. iMeta. 2023;2:e77 .Wdoi:10.1002/imt2.77

52. Dieterle MG, Rao K, Young VB. Novel therapies and preventative strategies for primary and recurrent Clostridium difficile infections. Ann N Y Acad Sci. 2019 Jan;1435(1):110-138. doi: 10.1111/nyas.13958

53. Yang X, Dong N, Chan EW, Zhang R, Chen S. Carbapenem Resistance-Encoding and Virulence-Encoding Conjugative Plasmids in Klebsiella pneumoniae. Trends Microbiol. 2021 Jan;29(1):65-83. doi: 10.1016/j.tim.2020.04.012

54. Jaillard M, Palmieri M, van Belkum A, Mahé P. Interpreting k-mer-based signatures for antibiotic resistance prediction. Gigascience. 2020 Oct 17;9(10):giaa110. doi: 10.1093/gigascience/giaa110

55. Sedlar K, Kupkova K, Provaznik I. Bioinformatics strategies for taxonomy independent binning and visualization of sequences in shotgun metagenomics. Comput Struct Biotechnol J. 2016 Dec 5;15:48-55. doi: 10.1016/j.csbj.2016.11.005

Table 1. Experimental and computational schemes developed for plasmid classification.

Technology category	Method	Classification scheme	Material	Description
Experimental	DPMT [12]	MOB typing	Plasmid DNA collections from clinical isolates	Using degenerate primers to hybridize relaxase-coding gene to identify and classify plasmids isolated from clinical isolates
	PlasTax-PCR [14]	Taxonomic typing	Plasmid DNA collections from clinical isolates	Utilized PCR primers that target conserved segments of the relaxase gene of plasmid taxonomic units (PTUs) to identify specific PTUs of transmissible plasmids
	PBRT [15]	Rep typing or Inc typing	Plasmid DNA collections from clinical isolates	Used multiplex PCR to amplify DNA fragments of replicon and detect known replicon types of plasmids
Computational	MOBscan [18]	MOB typing	Plasmid protein sequences	Used the HMMER model to annotated the relaxases and further perform MOB typing
	MOB-suite [19, 20]	MOB typing, MPF typing and Rep typing	Complete plasmid genomes or plasmid assembly clusters (Linux)	Utilized collected relaxase, oriT, replicon and T4SS sequences to construct database, then performing classification for plasmids assembly clusters with BLAST
	PlasTans [16]	transmissible plasmid identification	plasmid assembly contigs (Linux)	Using the convolutional neural network of the deep learning technique to classify plasmid DNA fragment
	PlasmidFinder [6]	Rep typing or Inc typing	Raw reads or complete plasmid genomes or plasmid assembly contigs (web server)	Utilized collected replicon sequences and BLASTn to perform Rep typing and Inc typing
	pMLST [6]	Rep typing or Inc typing	Raw reads or Complete plasmid genomes or plasmid assembly contigs (web server)	Used collected plasmid multilocus sequence typing (pMLST) allele sequences, known sequence type profiles and BLAST to perform Rep typing and Inc typing
	oriTfinder [17]	MOB typing, MPF typing	Complete plasmid genomes (web server)	Utilized collected oriT, relaxase, T4CP and T4SS sequences to annotate plasmids with BLAST

COPLA [5]	Taxonomic typing	Complete plasmid genomes or plasmid assembly sets (Linux)	Using average nucleotide identity (ANI) metrics and hierarchical stochastic block modeling (HSBM) to create plasmid taxonomic units (PTUs) and predict the taxonomic host

Table 2. The number, average length, and GC content of plasmid genomes for each MOB type.

Class	Number	Average length	GC (%)
MOBB	623	10921.77	51.27
MOBC	3218	19965.28	47.14
MOBF	21268	103802.80	52.07
MOBH	4880	151108.10	48.37
MOBL	3446	51430.63	34.57
MOBM	1761	2684.14	27.12
MOBP	15617	32237.88	49.70
MOBQ	9347	89357.64	56.77
MOBT	1181	11643.24	36.92
MOBV	4405	6595.43	37.75
Non-mob	24649	37581.85	49.84

Figure 1 Click here to access/download;Figure;Figure1.tif

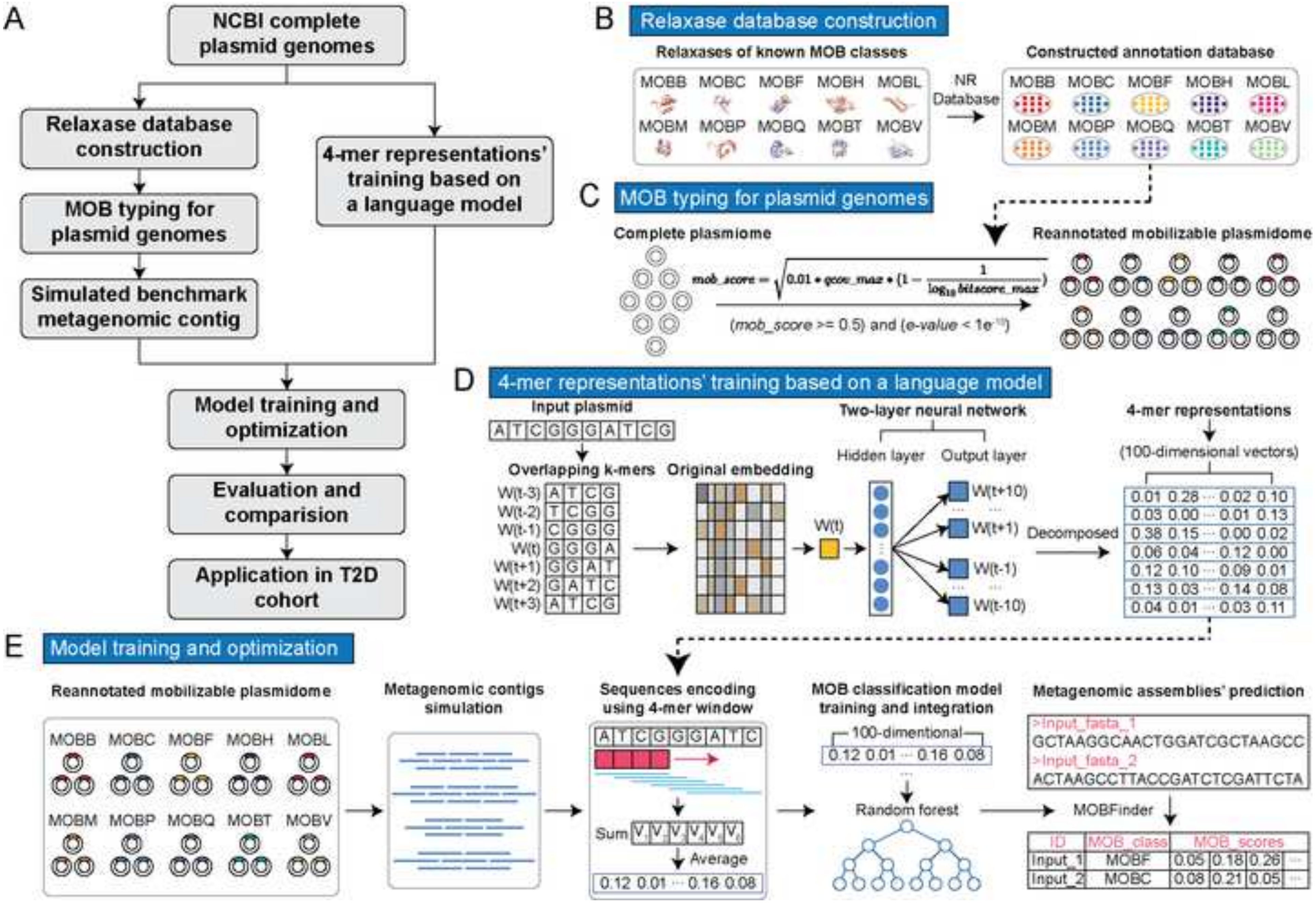

Figure 2

Click here to access/download;Figure;Figure2.tif

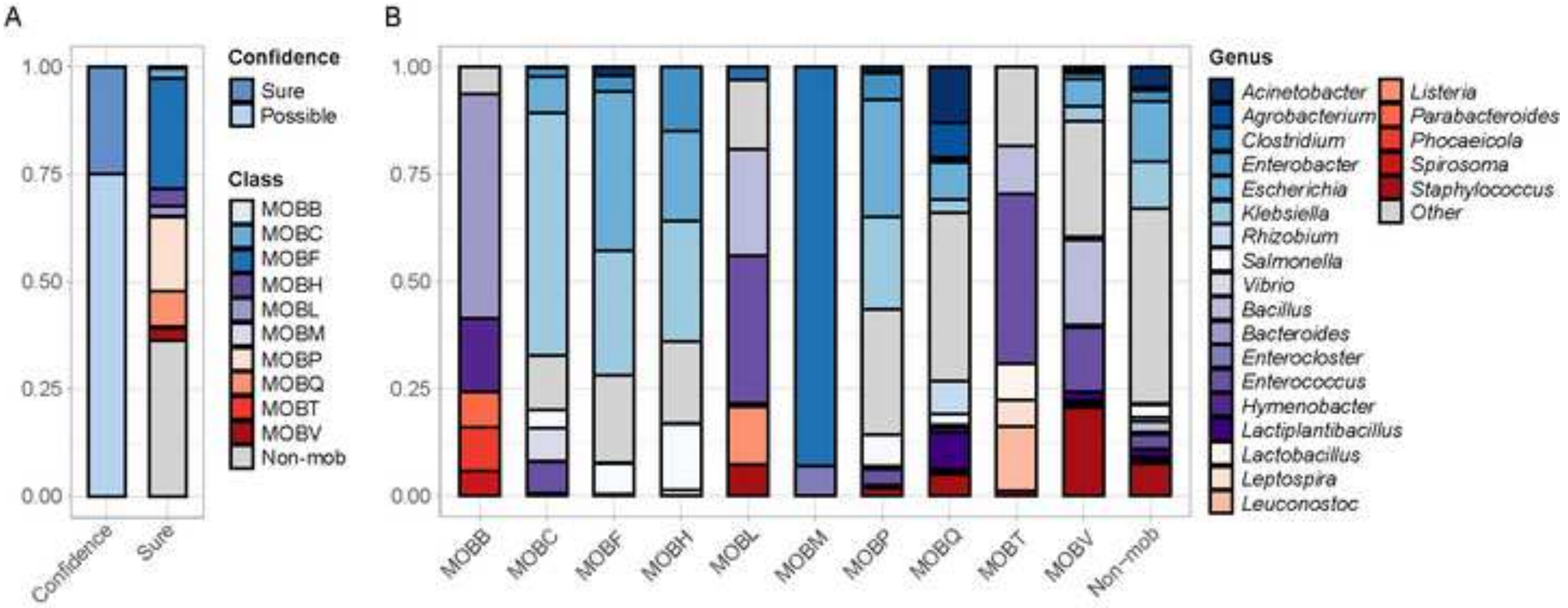

Figure 3 Click here to access/download;Figure;Figure3.tif

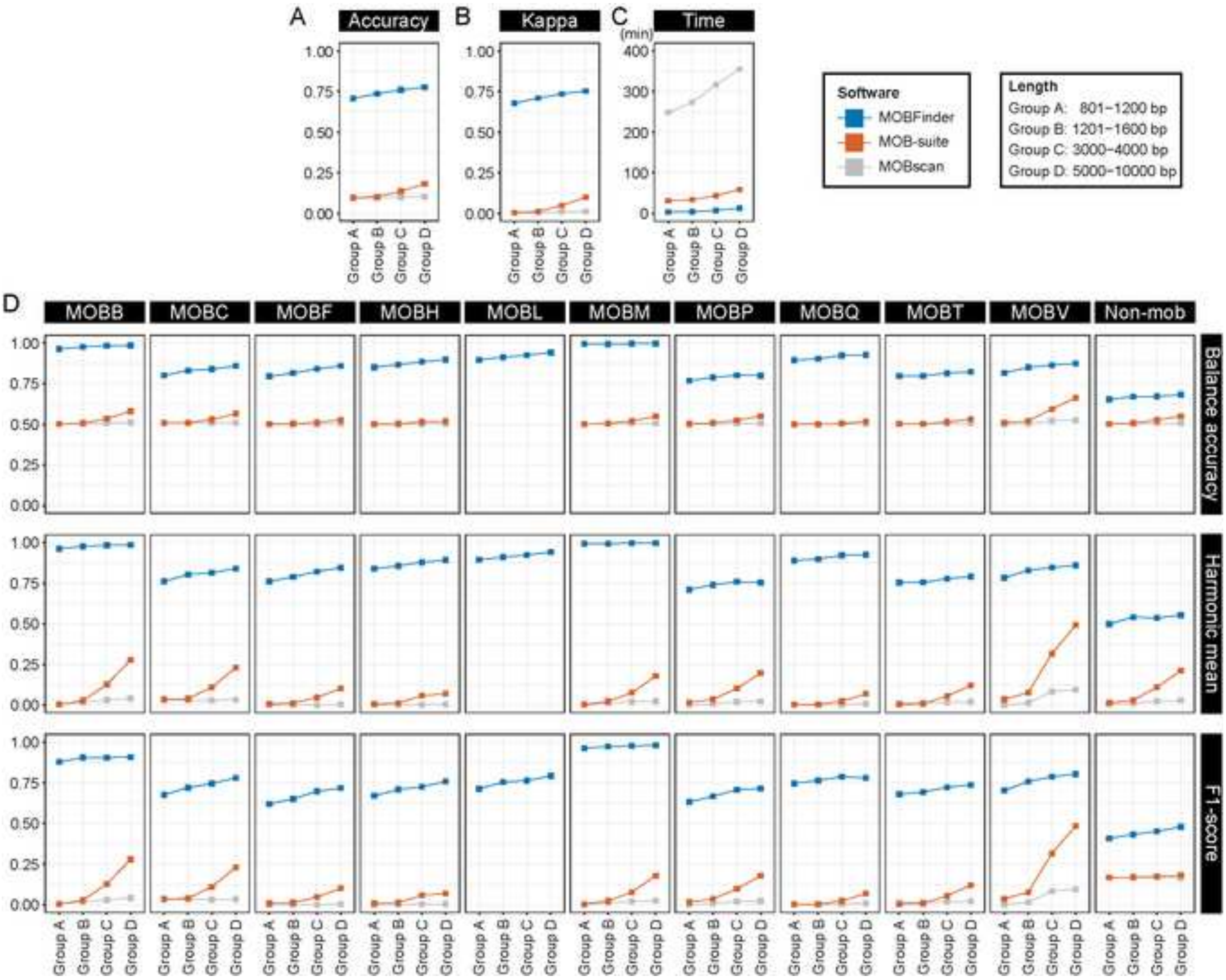

Figure 4

Click here to access/download;Figure;Figure4.tif

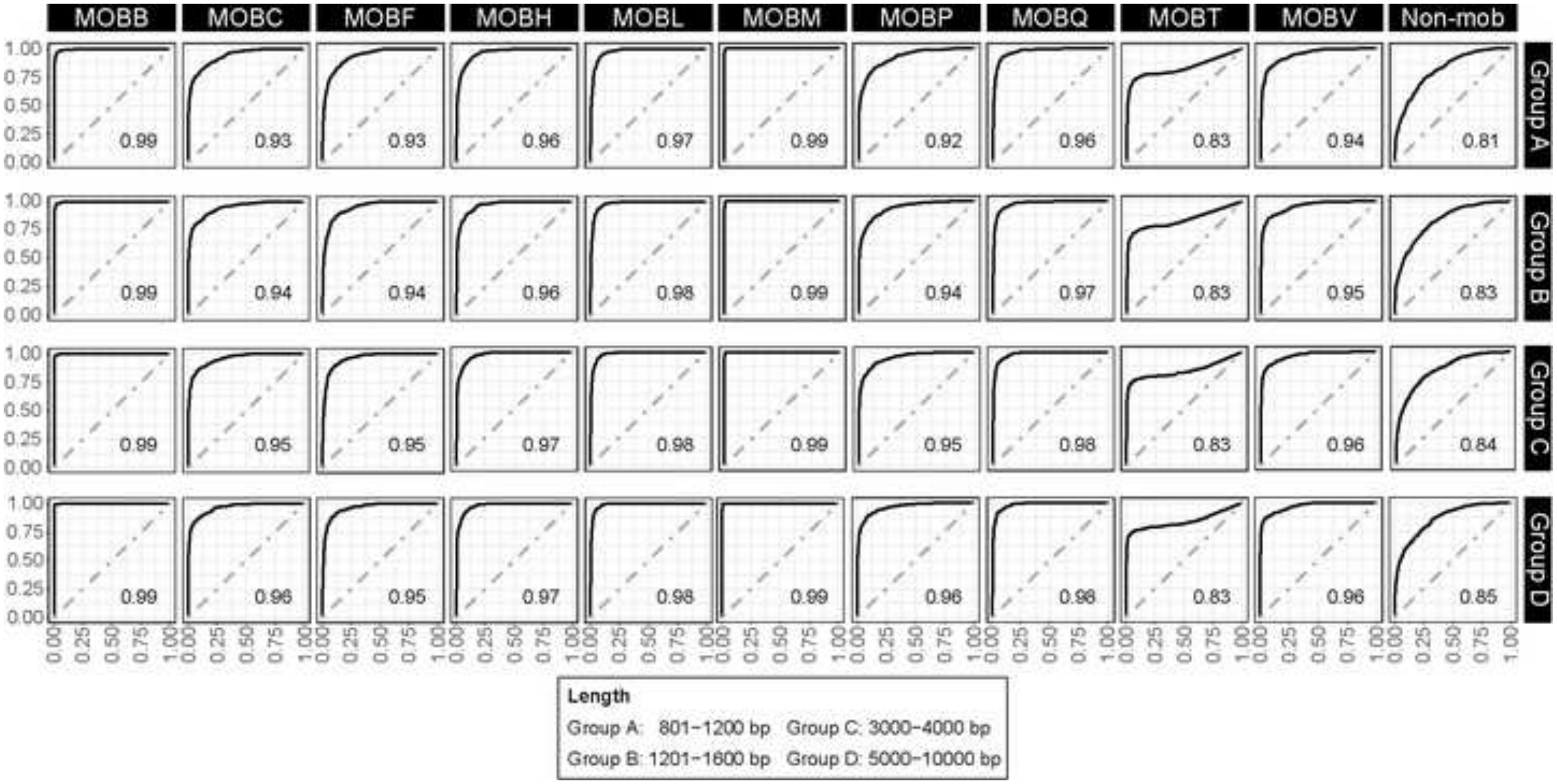

Figure 5

Click here to access/download;Figure;Figure5.tif

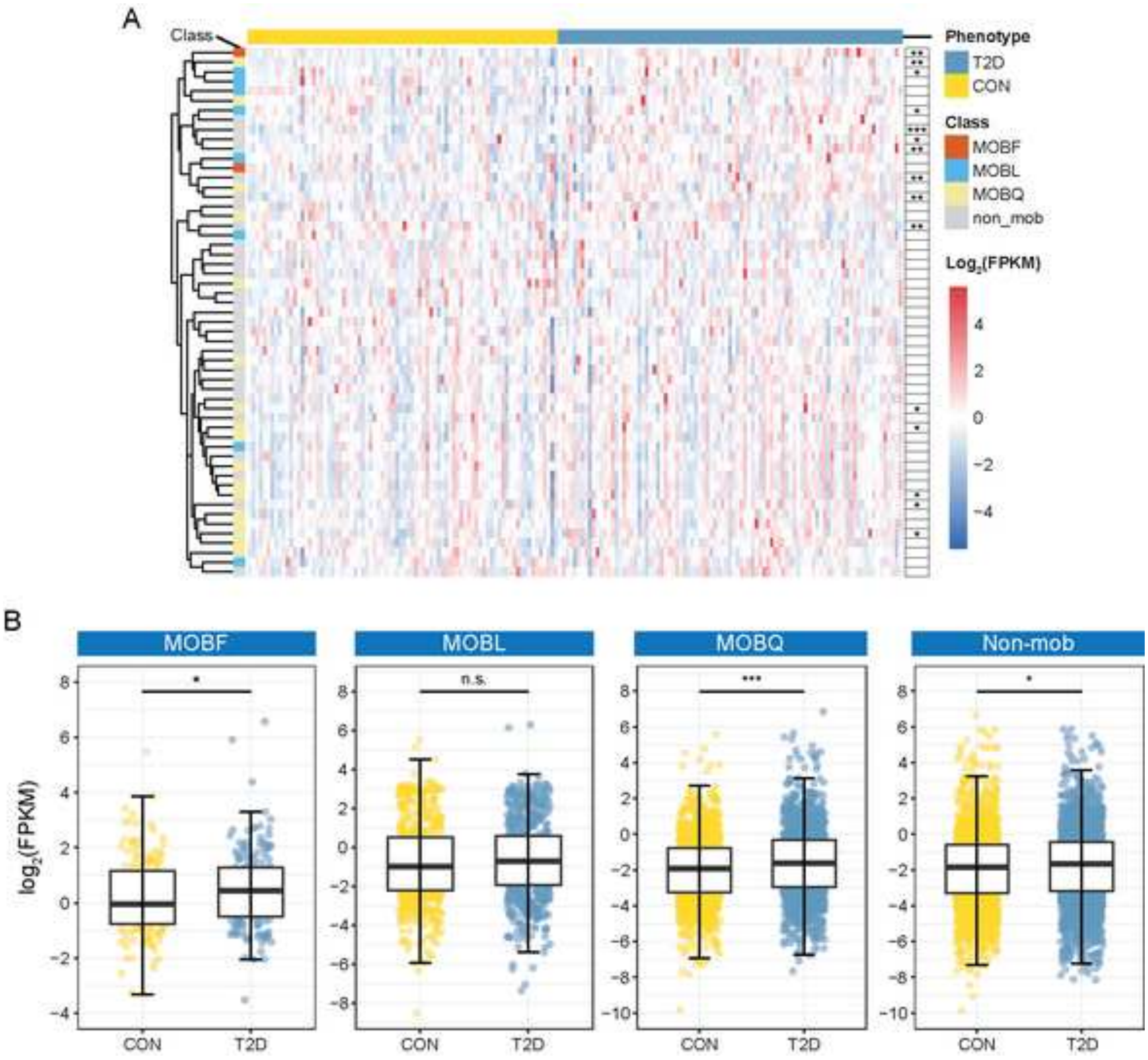

Click here to access/download;Figure;Figure6.tif

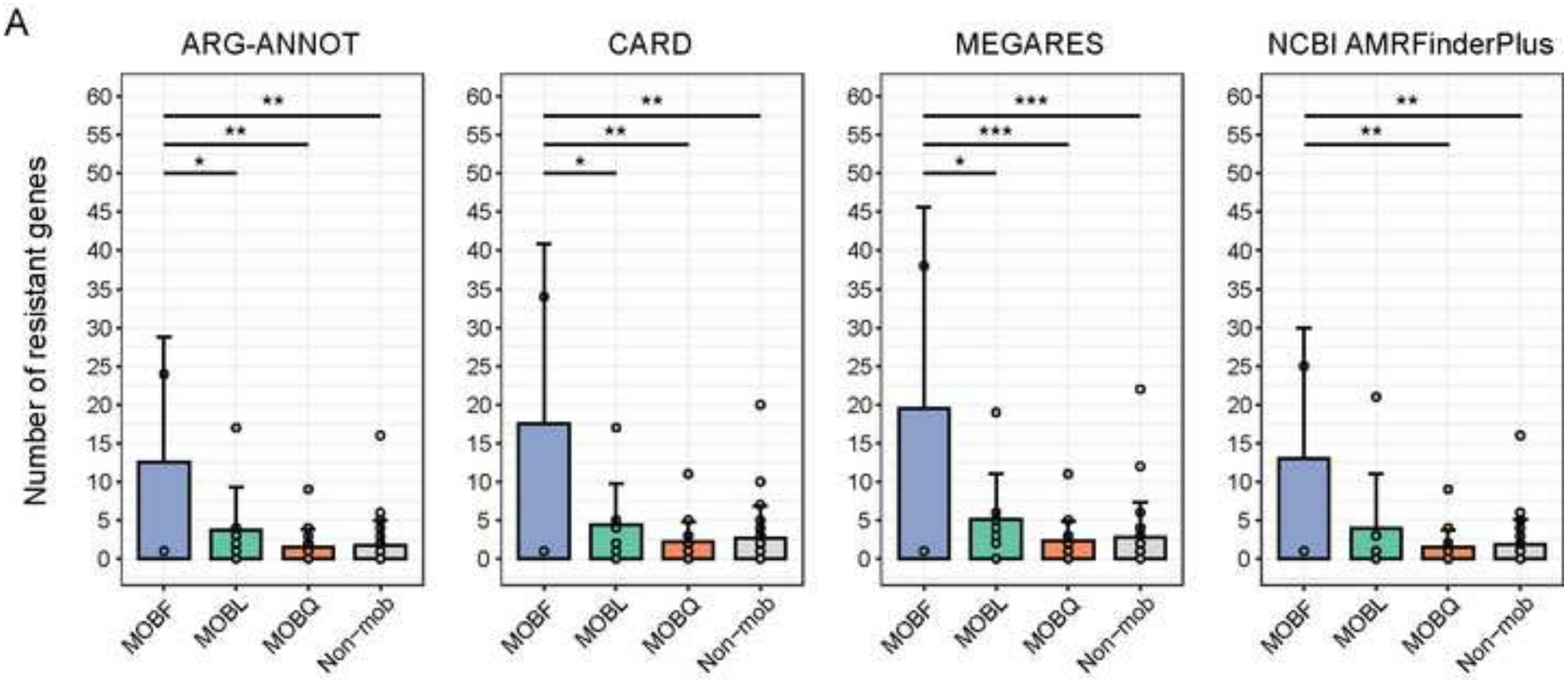

Supplementary Figures

Click here to access/download
Supplementary Material
Suplplementaty Figures.docx

Supplementary Table 1

Click here to access/download
Supplementary Material
Suplplementaty Table 1.xlsx

Supplementary Table 2

Click here to access/download

Supplementary Material

Suplplementaty Table 2.xlsx

Personal Cover
Click here to access/download;Personal Cover;cover letter.docx

Cover Letter

Dear Editor,

Thank you very much for you previous E-mail on January 05, 2024, regarding our manuscript, "MOBFinder: a tool for MOB typing for plasmid metagenomic fragments based on language model" (Manuscript Number: GIGA-D-23-00284). First, we would like to express our sincere gratitude for giving us the chance to revise and resubmit our work. Meanwhile, we are really appreciative of two Reviewer's overall positive comments about out work. We appreciate so much substantial and valuable advice and comments from you and two Reviewers, which absolutely helped up improve our work.

Following your advice and two Reviewer's comments, we have made an effort to revise the manuscript substantially. In this cover letter and the revised manuscript, we used red text for all the changed works, sentences, or paragraphs.

Before we report out response to Reviewers' comments, we would like to first summarize some major revisions in the revised manuscript as follows:

1. We added the results of a performance comparison between MOBFinder and MOBscan in the revised manuscript.

Following Reviewer 1's suggestion, we compared the performance of MOBFinder, MOB-suite, and MOBscan in predicting different MOB types. Using the same test data, we tested the performance of MOBFinder, MOB-suite, and MOBscan within the following length ranges: 801-1200bp, 1201-1600bp, 3000-4000bp, and 5000-10000bp. Since MOBscan can only predict the MOB type with plasmid proteins, we annotated the plasmids in the test set with Prokka, then manually submitted them to the MOBscan website for MOB type annotation.

<u>Compared to MOB-suite and MOBscan, MOBFinder showed the best overall performance in predicting metagenomic plasmid assembly fragments.</u> We evaluated the comprehensive performance of the three tools using *overall accuracy*, *kappa* values, and *running time*. The results showed that MOBFinder had the highest *overall accuracy*, at least 0.59 higher than MOB-suite and at least 0.61 higher than MOBscan. MOBFinder's *kappa* value was at least 0.65 higher than MOB-suite and at least 0.67 higher than MOBscan. MOBFinder's *running time* was only 1/5 of MOB-suite's and 1/28 of MOBscan's. In tests of each MOB type, MOBFinder's *balanced accuracy*, *harmonic mean*, and *F1-score* was significantly higher than those of MOB-

suite and MOBscan.

2. We evaluated the impact of word vectors trained with different *k*-mer lengths on the performance of MOBFinder, confirming that word vectors trained with 4-mer as the digital feature for metagenomic plasmid fragments offer the better comprehensive performance.

Following the suggestions of Reviewer 1 and Reviewer 2, we used the plasmid genomes as input and then trained word vectors with different lengths of *k*-mer using the skip-gram model. Using the word vectors trained with different *k*-mer length, we encoded the metagenomic plasmid fragments of each MOB type in the training set and trained a random forest classification model. Using the same test data, we evaluated the classification models trained with word vectors of different *k*-mer lengths.

After comparing the classification models of word vectors corresponding to different *k*-mer lengths, **we found that the model trained with word vectors of 4-mer had better overall performance, achieving higher *accuracy* with shorter *running time***. We evaluated the comprehensive performance of the classification models for different *k*-mer lengths using *overall accuracy*, *kappa* values, and *running time*. We found that the model trained with word vectors of *k*-mer length 2 had the lowest *overall accuracy* and *kappa* values. At *k*-mer length of 4, the classification model's *overall accuracy* and *kappa* values tended to stabilize, with short running times. For $k>4$, there was no significant increase in *overall accuracy* and *kappa* values, but the *running time* gradually increased. For each MOB type, we compared the *balanced accuracy*, *harmonic mean*, and *F1-score* of the classification models trained with word vectors of different *k*-mer lengths. We found that at *k*-mer length of 4, the three metrics for each MOB type tended to stabilize. Therefore, we chose to train the MOB classification model with word vectors trained with a *k*-mer length of 4.

3. We have redrawn the workflow diagram of Figure 1 to enhance the clarity of the construction process of MOBFinder for the readers.

In response to Reviewer 2's comments, which highlighted a lack of detailed description of certain technical aspects in the original illustration, such as "*how feature word vectors are generated, and how they are integrated with the random forest model*", we have significantly revised Figure 1. In the updated figure, we commence with a subfigure that outlines the workflow diagram for constructing MOBFinder. Following this, each key step is depicted through separate subfigures, allowing readers to gain a clearer understanding of the working principles of MOBFinder through these visual representations accompanied by corresponding textual descriptions.

5. We have made minor revisions to correct grammatical errors and clarify unclear statements, as well as rectify minor flaws in our analysis process without altering the conclusions.

After a thorough checking of the manuscript, and in consideration of the comments provided by two reviewers, we have corrected various grammatical and spelling errors. Additionally, we have addressed instances of unclear statements or flawed analytical processes. All amendments have been highlighted in the revised manuscript using red font. Below, we will describe some of the principal modifications made.

(1) In our analysis of the T2D population, the primary conclusion drawn was that MOBF type and MOBQ type plasmids may expedite the transmission of antibiotic resistance among T2D patients. This conclusion stems from our observation that, compared to healthy individuals, these two types of plasmids exhibit a significantly higher abundance in T2D patients and carry a greater number of antibiotic resistance genes. Moreover, the bacterial hosts for MOBF plasmids are predominantly pathogenic strains of *Escherichia* and *Klebsiella*. As noted in Line 488-489 of the manuscript, "**This suggested that the increase of MOBF-type and MOBQ-type plasmids could potentially raise the risk of infection among individuals with T2D**". However, upon reviewing the abstract and certain sections of the text, we realized that only MOBF was mentioned, omitting references to MOBQ. In the revised manuscript, we have rectified this by incorporating descriptions of MOBQ where relevant. In Line 62-65 of the "Abstract" Section, we have revised the sentence as "In an application focused on a T2D cohort, MOBFinder offered insights suggesting that the MOBF type plasmid, which is widely present in *Escherichia* and *Klebsiella*, and MOBQ type plasmid, might accelerate the antibiotic resistance transmission in patients suffering from T2D"; In Line 471-474 of the Section "3.4. Application in T2D metagenomic analysis" in the revised manuscript, the sentence has been revised as "Among above MOB types, MOBQ contains the highest number of bins enriched in T2D, while MOBF is widely present in *Escherichia* and *Klebsiella* (Figure 2B), and some strains in *Klebsiella* is resistant to multiple antibiotics, including carbapenems [54], indicating that these two kinds of MOB types' plasmid might contribute to the antibiotic resistance enrichment in T2D."

(2) In Line 377, we have specified the thresholds for gene annotation using ABRicate as "(*identity*>50% and *qcov*>50%)". Given the complexity of plasmid sequences, employing relatively lenient thresholds for gene annotation in plasmid metagenomes is more appropriate (Ref: PMID: 32091572).

(3) In the revised manuscript, Figure 5 displays slight differences in the FPKM values for each bin compared to the original manuscript. This variation is attributed to the initial FPKM calculations not adjusting for read length. We have rectified this in the revised manuscript by implementing the necessary read length corrections. Additionally, based on Figure 5B, we have incorporated a supplementary figure (Figure S2) to more specifically showcase the abundance of each significantly different plasmid bin from various types between the T2D and CON groups, thus rendering the results display more detailed.

(4) In Line 250, the softmax function has been revised to “negative sampling function”, This modification is made because the standard softmax function is not employed here; rather, negative sampling is used to enhance the efficiency of the softmax function.

(5) Due to the addition of substantial content in the discussion section of the revised manuscript, we have removed the second paragraph from the original manuscript's discussion, as its related content has already been described in the results section. This deletion makes the discussion more concise.

(6) In the “Classification algorithm design” section, we removed formulas (b) and (c) from the original manuscript. Their operations are simple and well-described in the text, making their technical presentation unnecessary for reader comprehension.

(7) In the section “Application in T2D metagenomic analysis”, we have removed the sentence from the original manuscript that stated, “Since almost half of the plasmid bins were classified into multiple MOB classes using MOB-suite [18, 19], we excluded them from further analysis”. This described operation is irrelevant to the analytical steps of our paper and was mistakenly included from another text during manuscript preparation. We apologize for this oversight.

(8) In Line 84-85 of the “Introduction” section, “known as replicons” has been revised as “known as replication initiation (Rep) protein [4, 6]”. The original term, replicons, was not very precise.

(9) Some other grammatical and spelling errors and unclear statements have been revised. For example, in Line 151, “input each fragment” was revised to “each input fragment”; in Line 224, we added the phrase “with highest bit score” to make the

meaning of the sentence clearer; in Line 365, “generate metagenomic fragments” has been revised as “generate metagenomic contigs”, since unassembled reads can also be referred to as fragments, the use of the term “contigs” is more accurate; in Line 309-310, We have emphasized that the average score is a “weighted average scores based on the sequence length” to more clearly articulate the computational process of the tool.

We then report our revisions and responses to two reviewers’ all comments (italic text) one by one as follows:

To Reviewer #1:

General Comments:

The authors developed MOBFinder, a tool based on a language model for MOB typing of plasmid metagenomic fragments. They claim that 'MOBFinder, integrating multiple random forest models, demonstrated superior overall performance compared to existing tools.' However, I suggest that MOBFinder should be compared not only with MOB-typer (using BLAST) but also with MOBscan (using HMMER). Additionally, the authors are encouraged to explore different parameters. For instance, they should provide clarification on why a 4-mer sliding window was chosen. If possible, they should conduct a performance comparison with various window sizes to demonstrate the superiority of 4-mer over other k-mers, such as k = 2, 3, 5, 6, etc.

We are very grateful for Reviewer 1’s suggestions, which have helped us better demonstrate the advantages of MOBFinder. We appreciate Reviewer 1’s recommendation to include a comparison between MOBFinder and MOBscan, aiding in a more effective evaluation of MOBFinder’s performance. Additionally, we agree with the suggestion of using different *k*-mer lengths for word vector training and comparison. We have recognized the importance of these suggestions and have made corresponding modifications in the revised manuscript. The specific details of our response to Reviewer 1 are as follows.

Specific Comments:

1. page 7: line 26

Why was a 4-mer sliding window used? Was the performance compared with different window sizes? Is 4-mer better than other k-mers, such as k = 2, 3, 5, 6, etc.?

We apologize for not providing a detailed explanation as to why we selected 4-mer as the basic unit for word vector training in our study. Actually, **in metagenomic**

sequences classification task, 4-mer is the most widely used as the basic unit in various bioinformatics tools (Ref: PMID: 27980708). In this work, we also found that **the model trained with word vectors of 4-mer had better overall performance, achieving higher *accuracy* with shorter *running time***. We evaluated the comprehensive performance of the classification models for different *k*-mer lengths using *overall accuracy*, *kappa* values, and *running time*. We found that the model trained with word vectors of *k*-mer length 2 had the lowest overall *accuracy* and *kappa* values. At *k*-mer length of 4, the classification model's overall *accuracy* and *kappa* values tended to stabilize, with short *running times*. For *k*>4, there was no significant increase in *overall accuracy* and *kappa* values, but the *running time* gradually increased. For each MOB type, we compared the *balanced accuracy*, *harmonic mean*, and *F1-score* of the classification models trained with word vectors of different *k*-mer lengths. We found that at *k*-mer length of 4, the three metrics for each MOB type tended to stabilize. Therefore, we chose to train the MOB classification model with word vectors trained with a *k*-mer length of 4.

To provide readers with a clearer understanding of our rationale for choosing 4-mer for word vector training, we have included the following description in Section "Discussion":

In metagenomic sequences classification task, 4-mer is the most widely used as the basic unit in various bioinformatics tools [56], and MOBFinder also takes the 4-mer as a "word". To assess the impact of training word vectors with different *k*-mer lengths on performance, we compared models with *k*-mer lengths of 2, 3, 4, 5, 6, 7, and 8 (Figure S3). We observed lower overall *accuracy* and *kappa* values for *k*=2. At *k*=4, the *balanced accuracy, harmonic mean, F1-score,* and *AUC* values stabilized across different MOB types. Subsequently, as the *k*-mer length increased, there was no significant improvement in *accuracy* or other metrics, while the *run time* gradually increased. Therefore, we chose a *k*-mer length of 4 for training word vectors and developing MOBFinder. (Please refer to Line 554-561 in the revised manuscript).

We have displayed the performance comparison results of word vectors trained with different *k*-mer lengths, including 2, 3, 4, 5, 6, 7, 8, in Supplementary Figure 3.

2. page 10: line 19-20

I wonder if the p-values were adjusted for multiple comparisons. When using the 'Wilcoxon signed-rank two-sided test,' it is essential to clearly specify the pairs between the two comparative groups, namely T2D and control. For example, this information

should be shown in 'Supplementary Table 2. The list of metagenomic samples used in our analysis.'

Regarding the first point of this comment, we are very grateful to Reviewer 1 for reminding us of the necessity to adjust the *p*-values for multiple comparisons. In the original manuscript, we did not adjust these *p*-values. In the revised manuscript, we have applied the Benjamini-Hochberg (BH) method to correct all *p*-values presented in Figure 5. **Although the adjusted *p*-values are slightly increased, this does not affect the conclusions related to their significance.** Additionally, since the *p*-value calculations presented in Figure 6 were derived from Tukey's Honest Significant Difference test, which is inherently designed for multiple comparisons, there is no need for further adjustment of the *p*-values.

In response to the second point raised in this comment, we express our gratitude to Reviewer 1 for the helpful discussion on the use of paired tests. We would like to clarify that the hypothesis tests discussed here involve comparisons between two distinct groups, namely the case and control groups. Importantly, the samples within these groups do not come from the same individuals, rendering the application of paired tests inappropriate for our analysis. Paired tests are predominantly suited for scenarios such as intervention studies, where the objective is to assess the variation of a certain metric in the same subjects before and after an intervention. This methodology relies on the premise that the pre- and post-intervention samples are obtained from the identical subjects, thereby justifying the use of paired tests. Conversely, in the context of comparisons between case and control groups, the prerequisites for employing paired tests are generally not satisfied.

In the manuscript, we have revised the relevant descriptions to clarify the adjustments made to the *p*-values, as well as to indicate that the hypothesis tests employed are unpaired. The pertinent descriptions are as follows:

We conducted an analysis on the significance of differences of plasmid bins and various MOB types between healthy and T2D groups using the unpaired Wilcoxon signed-rank two-sided test. The calculation of *p* values was adjusted for multiple comparisons using the Benjamini-Hochberg (BH) method (denoted as *p.adjust*). (Please refer to Line 373-376 in the revised manuscript).

Additionally, in Line 476-483 in the revised manuscript, we have made corresponding modifications to the description of the p-values in the legend of Figure

5:

Figure 5. The annotation of T2D-related plasmid bins using MOBFinder. (A). Heatmap of plasmid bins between T2D and control. Each column represents a sample, and each row represents a plasmid bin. For each column, the color blue represents the T2D group, and the color yellow represents the control group. The four distinct colors on the y-axis represent identified four different MOB types using MOBFinder. (B). The abundance comparison the four identified MOB types between T2D and control. The *p-value* was calculated using Wilcoxon signed-rank two-sided test, adjusted by "BH" method for multiple comparisons. ("*" represents *p.adjust* < 0.01, "**" represents *p.adjust* < 0.05 and "***" represents *p.adjust* < 0.001.)

3. page 13: line 28-29
'MOBFidner' should be 'MOBFinder'.

We thank Reviewer 1 for the careful checking of our manuscript, and we have revised MOBFidner to MOBFinder in Line 520 of the revised manuscript.

4. #) page 13: line 36-37
The authors should compare the performance not only with MOB-typer (using BLAST) but also with MOBscan (using HMMER).
https://github.com/santirdnd/COPLA/
The MOBscan relaxase database was downloaded from https://castillo.dicom.unican.es/mobscan_about

We are deeply grateful to Reviewer 1 for the suggestion to compare MOBFinder with MOBscan, which has been immensely beneficial in further enhancing the quality of our manuscript. In the revised manuscript, we compared the performance of MOBFinder, MOB-suite, and MOBscan in predicting different MOB types. Using the same test data, we tested the performance of MOBFinder, MOB-suite, and MOBscan within the following length ranges: 801-1200bp, 1201-1600bp, 3000-4000bp, and 5000-10000bp. Since MOBscan can only predict the MOB type with plasmid proteins, we annotated the plasmids in the test set with Prokka, then manually submitted them to the MOBscan website for MOB type annotation.

Compared to MOB-suite and MOBscan, MOBFinder showed the best overall performance in predicting metagenomic plasmid assembly fragments. We evaluated the comprehensive performance of the three tools using *overall accuracy*,

kappa values, and *running time*. The results showed that MOBFinder had the highest *overall accuracy*, at least 0.59 higher than MOB-suite and at least 0.61 higher than MOBscan. MOBFinder's *kappa* value was at least 0.65 higher than MOB-suite and at least 0.67 higher than MOBscan. MOBFinder's *running time* was only 1/5 of MOB-suite's and 1/28 of MOBscan's. In tests of each MOB type, MOBFinder's *balanced accuracy*, *harmonic mean*, and *F1-score* was significantly higher than those of MOB-suite and MOBscan.

In the main text, within the Section "2.6 Performance validation", we have supplemented our manuscript with a description of the comparison between MOBFinder and MOBscan as follows:

"Test dataset was used to assess the performance of MOBFinder, and compared to MOB-suite and MOBscan. Since MOBscan can only predict the MOB type using plasmid protein sequences rather than DNA sequences, we first annotated the proteins in the plasmid fragments of the test set using Prokka [38] and then used MOBscan to predict the MOB type based on the annotated proteins. We calculated overall *accuracy*, *kappa* and *run time* by comparing the predicted classes and true classes. Given that MOBScan operates as an online tool and cannot be executed locally, the calculation of MOBScan's *run time* was confined to the duration spent on preprocessing with Prokka locally." (Please refer to Line 313-319 in the revised manuscript).

In the main text, under the section "3.2. Overall performance of MOBFinder," we have added a description of the comparison results between MOBFinder and MOBscan as follows:

In the comparison, it was observed that MOBscan did not perform well, achieving low *accuracy* and *kappa* values across sequences of varying lengths, while MOB-suite exhibited marginally better performance than MOBscan when handling sequences of greater length (Figure 3A, 3B). (Please refer to Line 418-421 in the revised manuscript).

Due to the inclusion of MOBscan results in Figure 3, we have made the following modifications to the legend of the figure (please refer to Line 429-435 in the revised manuscript):

Figure 3. Overall performance of MOBFinder and comparison to MOB-suite and MOBScan. (A-C) The performance evaluation and comparison between MOBFinder, MOB-suite and MOBscan using *accuracy* (A), *kappa* (B) and *run time* (C). In test

datasets, four length groups were generated: Group A: 801-1200 bp, Group B: 1201-1600 bp, Group C: 3000-4000 bp and Group D: 500-10000 bp. (D) For each MOB type, the *balanced accuracy*, *harmonic mean* and *F1-score* were used to assess the performance of MOBFinder and compared to MOB-suite and MOBscan. MOBFinder, MOB-suit and MOBscan are represented by blue lines, orange lines and gray lines respectively.

Moreover, we have added a description of MOBscan in Section “1. Introduction” as follows: “For the MOB typing, MOBscan [18] uses the HMMER model to annotated the relaxases and further perform MOB typing.” (Please refer to Line 108-110 in the revised manuscript). Additionally, in Table 1 of the revised manuscript, we have also included a description of MOBscan.

Furthermore, we have also supplemented the “Abstract” with relevant content regarding MOBscan as follows: “Evaluating the tool over the benchmark dataset, MOBFinder demonstrates higher performance compared to the existing tools MOBscan and MOB-suite” (please refer to Line 59-61 in the revised manuscript).

5. page 26-27: Figure 5; Figure 6
I recommend presenting individual data points in Figure 5B and Figure 6 rather than using a bar graph (mean ± SE). This recommendation aligns with the perspective presented in the following paper:
https://journals.plos.org/plosbiology/article?id=10.1371/journal.pbio.1002128

We are very grateful for Reviewer 1's valuable suggestion. In Figure 5 of the revised manuscript, we have redrawn the box and scatter plots to show the expression of plasmid bins of different MOB types in each sample. In Figure 6 of the revised manuscript, we added data points for antibiotic resistance genes annotated within each MOB type plasmid bin and also introduced error bars for the histograms of each MOB type.

To Reviewer #2:

General Comments:
The authors have introduced an interesting and potentially impactful computational tool aimed at advancing our understanding of plasmid-mediated antibiotic resistance. The innovative use of a language model for MOB typing in plasmid metagenomics is commendable. However, the manuscript, in its current form, requires several revisions

for clarity, precision, and substantiation of the claims made.

The manuscript would benefit significantly from careful proofreading and correction of typographical errors, which detract from the overall readability and professional quality of the text. Furthermore, the technical descriptions and methodological justifications need to be more detailed to ensure the reproducibility of the research and the robustness of the tool's algorithm.

I believe that addressing these issues will greatly improve the manuscript and make it a strong contribution to the field of metagenomics.

Herein, we express our appreciation for Reviewer 2's positive comments on our current work, describing MOBFinder as "*an interesting and potentially impactful computational tool aimed at advancing our understanding of plasmid-mediated antibiotic resistance*". We would like to thank Reviewer 2 for these comments and suggestions, which were certainly helpful for us to improve our work. We are particularly grateful to Reviewer 2 for the meticulous review of our manuscript, pointing out several spelling and grammatical errors. For Reviewer 2's following concerns on our work, we present our responses and the corresponding improvements or revisions as follows.

Specific Comments:

1. Page 4, Line 11: The text "...relaxes information..." should be corrected to "relaxase information" to reflect accurate terminology associated with plasmid conjugation.

We apologize for this mistake. In Line 55 in the revised manuscript, we have revised "relaxes information" to "relaxase information".

2. Page 5, Line 3: There is a typographical error in the phrase "...possess dinstinct transmission...". It should be revised to "...possess distinct transmission..." to maintain the professionalism of the manuscript.

We thank Reviewer 2 for the careful checking of our manuscript. In Line 92 in the revised manuscript, we have revised "dinstinct" to "distinct".

3. Page 5, Line 22: The term "mash" appears to reference a proprietary noun and should be capitalized as "Mash" if it refers to a software or method name.

We are immensely grateful for the suggestion from Reviewer 2. The term "mash" referred to herein signifies a method for measuring distance. To clarify the sentence further, we have revised "mash" to "Mash distance" in Line 111 of the revised manuscript.

4. Page 6, Line 3: The statement regarding the potential significance of MOBFinder in understanding and addressing antibiotic resistance in specific patient populations is made without sufficient evidence. It is advisable to frame such claims conditionally or to emphasize the need for further research to substantiate these conclusions.

We sincerely appreciate Reviewer 2's discussion on the potential value of MOBFinder. We acknowledge that, despite the demonstration of MOBFinder's application in populations within this paper, it is inappropriate to use the results to support the statement that "MOBFinder has value in understanding and addressing antibiotic resistance in specific patient populations". Following Reviewer 2's advice, we have rephrased this statement to "This indicates that MOBFinder offers an effective data analysis approach for investigating plasmid-mediated horizontal gene transfer within microbial communities." (Please refer to Line 164-166 in the revised manuscript). This revision emphasizes MOBFinder as a novel tool that provides researchers with a new analysis method in data analysis, de-emphasizing the biological problems MOBFinder can solve, thereby making the article more rigorous.

5. Page 6, Line 28: A grammatical error is present in the phrase "...strategy to for categorizing...". This should be corrected to "...strategy for categorizing..." to ensure grammatical integrity.

We extend our gratitude once again to Reviewer 2 for identifying the grammatical error. In Line 173 of the revised manuscript, we have corrected "strategy to for categorizing" to "strategy for categorizing". We apologize for this oversight.

6. Page 6, Line 35: For a technical audience, a brief explanation of how feature word vectors are generated, and how they are integrated with the random forest model, would be beneficial. Providing this information would strengthen the credibility and reproducibility of the work.

We are deeply grateful to Reviewer 2 for the suggestions regarding the description of Figure 1. We apologize for our failure to clearly present and describe some technical details in Figure 1. In the revised manuscript, we have redrawn the workflow diagram in Figure 1 to enhance the clarity of the construction process of MOBFinder for our readers. In the updated figure, we commence with a subfigure that outlines the workflow diagram for constructing MOBFinder. Following this, each key step is depicted through separate subfigures, allowing readers to gain a clearer understanding of the working principles of MOBFinder through these visual representations accompanied by corresponding textual descriptions. Specifically, **we illustrate the process of generating word vectors in Figure 1D and use a dashed arrow to link the crucial information between Figure 1D and Figure 1E, demonstrating how the**

word vectors are integrated with the random forest model.

Due to our update to Figure 1, we have also rewritten the legend for Figure 1. The revised legend summarizes each step in the development of MOBFinder, emphasizing how word vectors are generated and how they are input into the random forest model. By reading Figure 1 and its legend, readers can fundamentally understand the main technical details of MOBFinder. The modified legend is as follows (please refer to Line 177-189 in the revised manuscript):

Figure 1. The overview of the technical approach utilized in this study. (A). The workflow for the development and testing of the MOBFinder tool. (B). Using plasmid relaxases with known MOB types as reference sequences, we developed a database of relaxases from the NR database representing different MOB types. (C). Utilizing the relaxase database constructed in (B), complete plasmid genomes from the NCBI were subjected to MOB typing. (D). Based on the plasmid complete genome data in NCBI, we trained a 4-mer language model using the skip-gram algorithm, allowing each 4-mer to be represented by a 100-dimensional word vector. For a DNA fragment, the average word vector of all 4-mers on its sequence serves as the feature vector for that DNA. (E). We constructed simulated metagenomic contigs from the plasmid complete genomes that had been MOB typed in (C) as a benchmark and encoded these contigs into word vectors. These word vectors were then used to train a random forest. The trained random forest, with metagenomic DNA fragments as input, can predict the MOB typing of the corresponding DNA fragment based on its word vectors.

Additionally, due to the inclusion of several new subfigures in Figure 1, we have made references to specific subfigures at relevant points in the revised manuscript whenever content related to a particular subfigure is discussed. This includes the following locations:

Line 153 in the Section “1. Introduction”: “The overview of this work is shown in Figure 1A”.

Line 174 in the Section “2.1. The workflow of MOBFinder”: “…we constructed a benchmark dataset using a high-resolution MOB typing strategy for categorizing complete plasmid genomes (Figure 1B, 1C)”.

Line 175 in the Section “2.1. The workflow of MOBFinder”: “Then, based on a language model and random forest, we designed an algorithm to perform MOB typing

for plasmid metagenomic fragments (Figure 1D, 1E)".

Line 203 in the Section "2.2. MOB typing for complete plasmid genomes": "Ten validated MOB relaxase protein families were collected, including MOBB, MOBC, MOBF, MOBH, MOBL, MOBM, MOBP, MOBQ, MOBT and MOBV [7-10,35,36] (Figure 1B)".

Line 228 in the Section "2.2. MOB typing for complete plasmid genomes": "…to facilitate the plasmid genome classification (Figure 1C)".

Line 236 in the Section "2.3. Word embeddings using a language model": "The training steps were as follows (Figure 1D)".

Line 284 in the Section "2.5. Classification algorithm design": "The detailed steps are as follows (Figure 1E)".

7. Page 6, Line 39: Is the described method a standard approach to MOB typing for plasmid genomes? The authors should discuss how they validate the correctness of this method.

We understand Reviewer 2's concern regarding the standard approach to MOB typing for plasmid genomes. We would like to clarify that the presence of a relaxase gene on a plasmid serves as an indicator of the plasmid's capability to transfer between cells, and **the classification of plasmid genomes based on relaxase gene sequence similarity has been widely accepted and can be considered a standard method** (Ref: PMID: 20805406; PMID: 25873913; PMID: 14975531; PMID: 19396961; PMID: 21711366). In the "Introduction" section, we have also addressed the biological rationale behind MOB typing based on relaxase sequence similarity as follows: "Compared to these methods, MOB typing, another classification scheme, classifies plasmids based on the relaxase gene, which is present in all transmissible plasmids [8-10]." (Please refer to Line 89-91 in the revised manuscript). While there may be slight variations in the method of calculating relaxase sequence similarity and the selection of thresholds across different studies, the fundamental concept remains consistent. **In this work, we primarily employ a BLAST-based sequence alignment method for the MOB typing of relaxases, which is a recognized approach** (Ref: PMID: 30052170; PMID: 25873913). Furthermore, in our response to Reviewer 2's Comment 8, we will elaborate on the rationale behind our selection of specific thresholds when analyzing BLAST results.

To better elucidate the rationale behind our approach, we have added the following description in Section “2.2. MOB typing for complete plasmid genomes” of the revised manuscript.

Traditionally, plasmid MOB typing of complete plasmid genomes has been a bioinformatics task based on the analysis of relaxase sequence similarity. The practice of annotating MOB types through BLAST similarity searches using representative sequences of different MOB type relaxases has gradually evolved into the standard method for MOB typing [4, 19, 20]. In this work, we aim to construct simulated metagenomic benchmark contigs using plasmid complete genome data with known MOB typing, and published works on plasmid complete genome MOB typings have included a relatively small number of plasmids in their analyses. To expand the MOB typing dataset for plasmid complete genomes, we annotated the newly collected plasmid complete genome data for MOB typing, utilizing relaxase information. (Please refer to Line 192-200 in the revised manuscript).

Lastly, we wish to emphasize that, given the relative maturity of techniques for MOB typing of complete plasmid genomes based on relaxase, the focus of this paper is not on how to improve MOB typing of relaxase per se. Instead, our research explores the strategies that can be employed for MOB typing within plasmid metagenomic sequence fragments, especially when these fragments lack relaxase sequences. Consequently, in constructing our benchmark dataset, we opted for a widely recognized method to perform MOB typing on complete plasmid genome data.

8. Page 6, Line 43: The selection criteria for the threshold are not discussed. It would be helpful if the authors could explain how the thresholds were chosen.

We apologize for not clearly explaining the selection of thresholds. In our response to the Comment 7 of Reviewer 2, we mentioned that the approach we employed for MOB typing of complete plasmid genomes is widely recognized. Regarding the choice of thresholds, we opted for slightly more stringent criteria than those used in published literature. The primary reason for selecting more stringent thresholds was to filter out relaxases that were annotated as belonging to multiple MOB types, thereby making our benchmark dataset more reliable. To clarify this point, we have added the following description in the revised manuscript:

In previous study, the selection criteria for homologous protein sequence searches are established with an *e-value* threshold of 1e-5, and minimum requirements for *query coverage* and *identity* set at 50% [4]. However, employing these criteria, we observed

that certain relaxases could be annotated as belonging to multiple MOB types. To eliminate ambiguous annotations and construct a more reliable dataset for the training of MOBFinder, we imposed stricter criteria for the homologous sequence search of relaxases, setting the *e-value* threshold to 1e-10, and raising both identity and query coverage to 70%. (Please refer to Line 206-212 in the revised manuscript).

9. Page 7, Line 26: The use of a 4-mer sliding window could result in the loss of information between nucleotides. The rationale and potential limitations of this strategy should be discussed.

We are very grateful to Reviewer 2 for the discussion on 4-mer word vectors. Firstly, we would like to emphasize that **incorporation of information between nucleotides into our model is a strength of word vectors, rather than a weakness**. Below, we will briefly further elucidate the concept of word vectors.

Word vectors are a representational technique for words in the field of natural language processing. Researchers hypothesize that words with similar meanings often have similar contexts across different texts, as their substitution does not significantly alter the sentence's meaning. The core idea of word vectors is to map contextually similar words to spatially similar points, with the coordinates of these points representing the word vectors. The relative positions of these points can reflect the meanings between different words. A classic example is that after word vector encoding, the word vectors of "king", "man", "woman", and "queen" satisfy the equation "king – man + woman = queen" (Ref: https://doi.org/10.48550/arXiv.1301.3781), demonstrating that word vector coordinates can reflect word meanings. When this technology is applied to biological sequences, including DNA and protein sequences, it has a similar effect: **by making use of the information between nucleotides, word vectors are able to capture the spatial information that reflects the biochemical functions and properties of the sequences**.

Compared to word vector models, traditional biological sequence representation models such as *k*-mer frequency vectors and one-hot encoding simply record the occurrence positions or frequencies of characters on the sequence and fail to capture the deeper meaning of these characters. To more effectively highlight the advantages of word vectors to our readers, we have added the following introduction to word vectors in the "Introduction" section: In this methodology, short sequences of nucleotides (referred to as *k*-mers) or amino acids are analogous to "words", and the longer sequences of DNA or proteins are analogous to "sentences". Through the application of unsupervised learning on large datasets, each "word" is linked to a feature vector that

captures its context, offering a more sophisticated analysis than the traditional *k*-mer frequency method, which simply counts the occurrence of nucleotide sequences without acknowledging their biochemical characteristics. Unlike the conventional method, this language model-based approach assesses sequences based on their contextual importance across different genetic environments, positioning contextually similar sequences close together in a multidimensional space. This technique provides deeper insights into the biochemical complexities of nucleotide sequences, thereby furnishing a more comprehensive understanding of an organism's functional biology [34]. (Please refer to Line 132-142 in the revised manuscript). In the text mentioned above, we have included the reference [34], through which readers can further explore the application of word vectors in the representation of biological sequences.

Additionally, regarding the rationale for selecting 4-mer as the basic unit for word vector encoding, whether this length is sufficient, and whether choosing different *k*-mer values might be more advantageous, we have also presented related results and explanations in the revised manuscript. After comparing the classification models of word vectors corresponding to different *k*-mer lengths, **we found that the model trained with word vectors of 4-mer had better overall performance, achieving higher *accuracy* with shorter *running time***. We evaluated the comprehensive performance of the classification models for different *k*-mer lengths using *overall accuracy*, *kappa* values, and *running time*. We found that the model trained with word vectors of *k*-mer length 2 had the lowest *overall accuracy* and *kappa* values. At *k*-mer length of 4, the classification model's *overall accuracy* and *kappa* values tended to stabilize, with short running times. For *k*>4, there was no significant increase in *overall accuracy* and *kappa* values, but the *running time* gradually increased. For each MOB type, we compared the *balanced accuracy*, *harmonic mean*, and *F1-score* of the classification models trained with word vectors of different *k*-mer lengths. We found that at *k*-mer length of 4, the three metrics for each MOB type tended to stabilize. Therefore, we chose to train the MOB classification model with word vectors trained with a *k*-mer length of 4.

To provide readers with a clearer understanding of our rationale for choosing 4-mer for word vector training, we have included the following description in Section "3.2 Overall performance of MOBFinder":

In metagenomic sequences classification task, 4-mer is the most widely used as the basic unit in various bioinformatics tools [56], and MOBFinder also takes the 4-mer as a "word". To assess the impact of training word vectors with different *k*-mer lengths on

performance, we compared models with *k*-mer lengths of 2, 3, 4, 5, 6, 7, and 8 (Figure S3). We observed lower overall *accuracy* and *kappa* values for *k*=2. At *k*=4, the *balanced accuracy*, *harmonic mean*, *F1-score*, and *AUC* values stabilized across different MOB types. Subsequently, as the *k*-mer length increased, there was no significant improvement in *accuracy* or other metrics, while the *run time* gradually increased. Therefore, we chose a *k*-mer length of 4 for training word vectors and developing MOBFinder. (Please refer to Line 554-561 in the revised manuscript).

10. Page 7, Line 33: The one-hot encoding process could lead to dimensionality issues, especially when dealing with large datasets. Discuss how this problem is addressed, and whether more efficient encoding methods were considered.

We understand Reviewer 2's concern regarding dimensionality issues. Firstly, we wish to correct a misstatement in our manuscript: we do not initialize word vectors with one-hot encoding but rather use a random vector, a method commonly used in word vector language models (Ref: https://doi.org/10.48550/arXiv.1301.3781). We have made several corrections to the related descriptions in Section "2.3. Word embeddings using a language model", including: in Line 243 of the revised manuscript, the sentence has been revised as: "Word encoding initialization. Each word is initially assigned a random vector."; in Line 247-249 of the revised manuscript, the sentence has been revised as: "The input is the initialized vectors, and the output is a probability distribution over the input words. Layer 1 is a hidden layer to convert the input initialized vectors into a 100-dimensional word vector representation as predefined by Ng [31]" We apologize for this inappropriate expression.

Below, we will discuss from two perspectives to elucidate why dimensionality issues do not constitute a major concern for our work.

(1) **Dimensionality issues often occur in scenarios where the number of features significantly exceeds the volume of training data, which can lead to overfitting. Nonetheless, this was not an issue we encountered in the process of pre-training the word vectors for MOBFinder.** Taking the skip-gram algorithm utilized in this study as an example, the dimension of the randomly initialized vector can be 1-of-*n*, where *n* represents the size of the vocabulary [30]. The dimensionality of this randomly initialized vector is the same as that of a one-hot vector. In MOBFinder, where we treat each 4-mer as a word, the vocabulary size becomes 4^4. Given that the number of complete plasmid genomes used for word vector pre-training exceeds 90,000, encompassing several billion bases, the volume of data far exceeds the number of features. Therefore, dimensionality issues do not arise.

(2) **<u>The training of word vectors inherently involves compressing high-dimensional initial vectors into lower-dimensional feature vectors, serving as a dimensionality reduction process</u>** [31]. Compared to characterizing biological sequences directly with traditional *k*-mer frequency models and one-hot models, employing word vector models for the representation of biological sequences generally poses fewer concerns regarding dimensionality issues.

Surrounding the content mentioned above, we have expanded our discussion on dimensionality issues in the "Discussion" section:

In the past, *k*-mer frequency models and one-hot encoding were commonly employed methods for digitizing biological sequences, extensively applied across various machine learning algorithms [55]. However, both models simply mark or count the frequency of various characters appearing in sequences, failing to profoundly reflect the biological significance underlying each character. Concurrently, these models may encounter dimensional issues [55]. For instance, in the *k*-mer model, if *k* is set to 8, the dimensionality of each DNA sequence's *k*-mer vector becomes 4^8, which is problematic in metagenomics where most fragment lengths do not reach this magnitude, resulting in significant noise in the feature vector and causing overfitting. Similarly, in the one-hot model, for a sequence of length *L* using 4-mers as the base unit, it would require *L* one-hot vectors each with a dimensionality of 4^4. In such instances, if the dataset for training is not sufficiently large, this representation method could also lead to overfitting due to high dimensionality. In contrast, word vector models offer a superior solution to these problems. Word vector models initially perform a random initialization of vectors for each word. Taking the skip-gram algorithm utilized in this study as an example, the dimension of this random vector can be 1-of-*n*, where *n* represents the size of the vocabulary [30]. Following unsupervised pre-training on large datasets, the algorithm maps characters with similar contexts to similar feature spaces. The dimensions of these feature spaces' coordinates (i.e., the word vectors) will be lower than those of the initial random vectors. Thus, through unsupervised pre-training driven by large datasets, language models can compress high-dimensional initial vectors into lower-dimensional word vectors (e.g., MOBFinder's word vectors with a dimensionality of 100), enabling these feature vectors to contain more character information while effectively avoiding dimensional issues in supervised training. (Please refer to Line 532-552 in the revised manuscript).

11. Page 7, Line 35: The description of the skip-gram model construction lacks detail

regarding the selection of network architecture, the number of units in the hidden layer, and the size of the context window.

We apologize for not clearly describing some details of the skip-gram model construction. The skip-gram model structure we adopted in this paper follows the standard structure proposed by the algorithm's original authors [30]. Given that this standard structure has been widely applied in various biological sequence representations and has shown stable performance (Ref: PMID: 30418485; PMID: 31492094; PMID: 33784381), we did not modify this structure (e.g., the number of units in the hidden layer). Additionally, Ng have provided a skip-gram algorithm interface for DNA sequences, dna2vec, based on the classic word2vec algorithm [31]. In this software package, the author has set some default parameters, such as the number of units in the hidden layer and the size of the context window, based on their preliminary experiments. We adopted these default settings in this work. In the development process of MOBFinder, our main focus was on exploring the length of *k*-mer. We have clarified our reasons for choosing *k*=4 as the basic unit for word vector encoding in our response to Comment 9 from Reviewer 2.

To provide a better description of these details, we have added the following explanation in Section "2.3. Word embeddings using a language model":

In Line 245-246 of the revised manuscript, we have added the sentence "We employ a standard skip-gram model as described in [30, 31] for the word vector generation through the dna2vec module [31]".

In Line 248-249 of the revised manuscript, we have revised the sentence as "Layer 1 is a hidden layer to convert the input initialized vectors into a 100-dimensional word vector representation as predefined by Ng [31].".

In Line 251-252 of the revised manuscript, we have added the description "the size of context words was set to 20 (10 words for upstream and downstream respectively) as pre-set by Ng [31]."

In Line 255-256, we have revised the sentence as "Using the default setting, we then used backpropagation to update the neural network parameters (word vectors) for 10 epochs".

12. Page 13, Line 29: A typographical error "MOBFidner" should be corrected to "MOBFinder" to maintain the accuracy of the text.

We apologize for this mistake, and we have revised “MOBFidner” to “MOBFinder” in Line 520 of the revised manuscript. We thank Reviewer 2 for pointing out this mistake.

13. Page 22, Figure B: The caption "Classification model enseble" contains a typographical error. The correct spelling should be "ensemble".

We express our gratitude to Reviewer 2 for the meticulous review of our manuscript. As mentioned in our response to Comment 6 of Reviewer 2, we have made significant modifications to Figure 1, and the revised Figure 1 no longer contains the error.

In hoping that the above revision has clarified all the points by two reviewers and given a point-by-point response to all the concerns, we hereby resubmit our manuscript to the journal. We thank you for your kind consideration.

Note: The initial version of the article has been made public on bioRxiv (https://doi.org/10.1101/2023.12.06.570414), and beyond that, it has not been published in any other traditional journals.

Sincerely yours,

Zhencheng Fang, Ph.D.
Microbiome Medicine Center, Department of Laboratory Medicine, Zhujiang Hospital, Southern Medical University, Guangzhou, 510280, China
Email: fangzc@smu.edu.cn
