# Supplementary material for: MOBFinder: a tool for mobilization typing of plasmid metagenomic fragments based on a language model
Source: Gigascience. 2024 Aug 5;13:giae047. doi: 10.1093/gigascience/giae047 (PMC11299106; doi:10.1093/gigascience/giae047)
Supplement: giae047_GIGA-D-24-00070_Revision_1 [file giae047_giga-d-24-00070_revision_1.pdf]

GigaScience

MOBFinder: A tool for mobilization typing for plasmid metagenomic fragments based on a language model

--Manuscript Draft--

**Manuscript Number:**	GIGA-D-24-00070R1
**Full Title:**	MOBFinder: A tool for mobilization typing for plasmid metagenomic fragments based on a language model
**Article Type:**	Technical Note
**Funding Information:**	National Key Research and Development Program of China (2022YFA0806400) — Prof. Hongwei Zhou<br>National Natural Science Foundation of China (82102508) — Dr. Zhencheng Fang<br>National Natural Science Foundation of China (81925026) — Prof. Hongwei Zhou
**Abstract:**	Background<br>Mobilization typing (MOB) is a classification scheme for plasmid genomes based on their relaxase gene. The host ranges of plasmids of different MOB categories are diverse and MOB is crucial for investigating plasmid mobilization, especially the transmission of resistance genes and virulence factors. However, MOB typing of plasmid metagenomic data is challenging due to the highly fragmented characteristics of metagenomic contigs.<br>Results<br>We developed MOBFinder, an 11-class classifier, for categorizing plasmid fragments into 10 MOB types and a non-mobilizable category. We first performed MOB typing to classify complete plasmid genomes according to relaxase information and then constructed an artificial benchmark dataset of plasmid metagenomic fragments (PMFs) from those complete plasmid genomes whose MOB types are well annotated. Next, based on natural language models, we used word vectors to characterize the PMFs. Several random forest classification models were trained and integrated to predict fragments of different lengths. Evaluating the tool using the benchmark dataset, we found that MOBFinder outperforms previous tools such as MOBscan and MOB-suite, with an overall accuracy approximately 59% higher than that of MOB-suite. Moreover, the balanced accuracy, harmonic mean, and F1-score reached up to 99% for some MOB types. When applied to a cohort of patients with type II diabetes (T2D), MOBFinder offered insights suggesting that the MOBF type plasmid, which is widely present in Escherichia and Klebsiella, and the MOBQ type plasmid, might accelerate antibiotic resistance transmission in patients suffering from T2D.<br>Conclusions<br>To the best of our knowledge, MOBFinder is the first tool for MOB typing of PMFs. The tool is freely available at https://github.com/FengTaoSMU/MOBFinder.
**Corresponding Author:**	Zhencheng Fang<br>Zhujiang Hospital of Southern Medical University<br>Guangzhou, CHINA
**Corresponding Author Secondary Information:**	
**Corresponding Author's Institution:**	Zhujiang Hospital of Southern Medical University
**Corresponding Author's Secondary Institution:**	
**First Author:**	Tao Feng
**First Author Secondary Information:**	
**Order of Authors:**	Tao Feng<br>Shufang Wu

Powered by Editorial Manager® and ProduXion Manager® from Aries Systems Corporation

	Hongwei Zhou
	Zhencheng Fang
**Order of Authors Secondary Information:**	
**Response to Reviewers:**	The response to specific reviewer and editor comments has been uploaded in the cover letter.
**Additional Information:**	
**Question**	**Response**
Are you submitting this manuscript to a special series or article collection?	No
**Experimental design and statistics**<br><br>Full details of the experimental design and statistical methods used should be given in the Methods section, as detailed in our Minimum Standards Reporting Checklist. Information essential to interpreting the data presented should be made available in the figure legends.<br><br>Have you included all the information requested in your manuscript?	Yes
**Resources**<br><br>A description of all resources used, including antibodies, cell lines, animals and software tools, with enough information to allow them to be uniquely identified, should be included in the Methods section. Authors are strongly encouraged to cite Research Resource Identifiers (RRIDs) for antibodies, model organisms and tools, where possible.<br><br>Have you included the information requested as detailed in our Minimum Standards Reporting Checklist?	Yes
**Availability of data and materials**<br><br>All datasets and code on which the conclusions of the paper rely must be either included in your submission or deposited in publicly available repositories	Yes

Powered by Editorial Manager® and ProduXion Manager® from Aries Systems Corporation

(where available and ethically appropriate), referencing such data using a unique identifier in the references and in the “Availability of Data and Materials” section of your manuscript.<br><br>Have you have met the above requirement as detailed in our Minimum Standards Reporting Checklist?	

Manuscript
Click here to access/download;Manuscript;manuscript (11).docx

MOBFinder: A tool for mobilization typing of plasmid metagenomic fragments based on a language model

Tao Feng[a], Shufang Wu[a], Hongwei Zhou[a, *], and Zhencheng Fang[a, *]

[a]Microbiome Medicine Center, Department of Laboratory Medicine, Zhujiang Hospital, Southern Medical University, Guangzhou 510280, China

[*]To whom correspondence should be addressed.

Institutional addresses: 253 Gongye Middle Avenue, Haizhu District, Guangzhou, Guangdong, China, 510280

Email addresses:
Tao Feng: fengtaosmu@foxmail.com
Shufang Wu: wu-shufang@pku.edu.cn
Hongwei Zhou: hzhou@smu.edu.cn
Zhencheng Fang: fangzc@smu.edu.cn

ORCID iDs:
Tao Feng [0000-0003-1984-4265], Shufang Wu [0000-0001-7793-2509], Hongwei Zhou [0000-0003-2472-8541], Zhencheng Fang [0000-0002-0137-4683].

Abstract

Background

Mobilization typing (MOB) is a classification scheme for plasmid genomes based on their relaxase gene. The host ranges of plasmids of different MOB categories are diverse and MOB is crucial for investigating plasmid mobilization, especially the transmission of resistance genes and virulence factors. However, MOB typing of plasmid metagenomic data is challenging due to the highly fragmented characteristics of metagenomic contigs.

Results

We developed MOBFinder, an 11-class classifier, for categorizing plasmid fragments into 10 MOB types and a non-mobilizable category. We first performed MOB typing to classify complete plasmid genomes according to relaxase information and then constructed an artificial benchmark dataset of plasmid metagenomic fragments (PMFs) from those complete plasmid genomes whose MOB types are well annotated. Next, based on natural language models, we used word vectors to characterize the PMFs. Several random forest classification models were trained and integrated to predict fragments of different lengths. Evaluating the tool using the benchmark dataset, we found that MOBFinder outperforms previous tools such as MOBscan and MOB-suite, with an overall *accuracy* approximately 59% higher than that of MOB-suite. Moreover, the *balanced accuracy*, *harmonic mean*, and *F1-score* reached up to 99% for some MOB types. When applied to a cohort of patients with type II diabetes (T2D), MOBFinder offered insights suggesting that the MOBF type plasmid, which is widely present in *Escherichia* and *Klebsiella*, and the MOBQ type plasmid, might accelerate antibiotic resistance transmission in patients suffering from T2D.

Conclusions

To the best of our knowledge, MOBFinder is the first tool for MOB typing of PMFs. The tool is freely available at https://github.com/FengTaoSMU/MOBFinder.

Keywords: MOB typing; language model; metagenomic sequencing; plasmid; random forest

1. Introduction

Plasmids are usually small, double-stranded, and circular DNA molecules found within bacterial cells [1]. Being separate from the bacterial chromosome, plasmids have the ability to replicate independently and can be transferred between bacteria through conjugation [2]. Bacteria, specifically pathogenic strains, can acquire antibiotic resistance genes or virulence factors via plasmid-mediated horizontal gene transfer, aiding their ability to adapt to various environments [3].

Plasmid classification is important for investigating multiple properties of plasmids, such as host range, replication patterns, and mobilization mechanisms [4]. Many classification schemes have been developed according to the distinct characteristics of plasmids, including taxonomic classification, replicon typing (Rep), incompatibility typing (Inc), mate-pair formation typing (MPF), and mobilization typing (MOB). In taxonomic classification, plasmids are categorized based on their host bacteria [5]. Rep typing classifies plasmids according to genes controlling their replication, known as replication initiation genes [4, 6]. Inc typing takes advantage of the fact that plasmids with similar replication or partition systems are incompatible within the same cell, categorizing plasmids based on compatibility [6]. MPF typing is based on genes encoding the MPF system, which consists of proteins that mediate contact and DNA exchange between donor and recipient cells during conjugation [4, 7]. Finally, MOB typing classifies plasmids based on the relaxase gene, which is present in all transmissible plasmids [8-10]. And plasmids with different relaxase types are categorized as different MOB types, each of possesses a distinct transmission mechanism that determines its taxonomic host range [4, 11]. This variation among different MOB types is critical in researching the spread of virulence traits, the emergence of antibiotic resistance, and the adaptation and evolution of bacteria. Moreover, MOB typing has been found to be effective for identifying novel mobilizable plasmids that were previously unassigned to any Rep or Inc types, and for investigating the mobilization characteristics of plasmids with similar mobilization systems [12, 13].

Recently, many experimental and computational schemes have been devised for plasmid typing, as well as to explore the diversity and functionality of plasmids (Table 1). For example, plasmid taxonomic PCR (PlasTax-PCR) [14], PCR-based replicon typing (PBRT) [15], and degenerate primer MOB typing (DPMT) [12] are multiplex PCR methods for identifying plasmids with

analogous replication or mobilization systems. PlasTrans, based on deep learning, identifies mobilizable metagenomic plasmid fragments [16]. Web servers such as PlasmidFinder [6], pMLST, and oriTfinder [17] were established based on collected maker gene databases and alignment-based methods to facilitate Rep, Inc, and MOB typing. COPLA [5], based on average nucleotide identity, performs taxonomic classifications of complete plasmid genomes with an overall accuracy of 41%. For the MOB typing, MOBscan [18] uses the HMMER model to annotate relaxase genes and classify plasmids accordingly. MOB-suite [19, 20] performs plasmid typing for plasmid assemblies. First, it uses Mash distance to cluster plasmid assemblies into clusters; then, it uses marker gene databases to annotate them.

Table 1. Experimental and computational schemes developed for plasmid classification.

Metagenomic sequencing makes it possible to obtain all plasmid DNA from microbial communities at once, and a number of computational tools for identifying plasmid fragments from metagenomic data have been developed, such as PlasFlow [21], PlasmidSeeker [22], PlasClass [23], PPR-Meta [24] and PlasForest [25]. As DNA fragments of plasmids and bacteria are intermingled in metagenomic data [26], recognizing the transmission mechanisms and host ranges of plasmids can be challenging. To this end, it is crucial to annotate MOB types of metagenomic plasmid fragments. However, this is difficult when plasmid assembly fragments are incomplete and essential genes for annotation are lacking. Therefore, it is worthwhile to consider alternative methods. Given that plasmids of the same MOB type have similar transmission mechanisms and host ranges, their genomic signatures (e.g., GC content and codon usage) tend to also be alike, not only relaxase [4, 27]. In this context, neural networks, which have demonstrated strong performance in the classification and identification of biological sequences [28, 29] could be useful. Furthermore, language models [30, 31] derived from such neural networks have also showcased their impressive ability to characterize sequence features [32, 33]. In this methodology, short sequences of nucleotides (referred to as *k*-mers) or amino acids are analogous to “words”, and the longer sequences of DNA or proteins are analogous to “sentences”. Through the application of unsupervised learning on large datasets, each “word” is linked to a feature vector that captures its context, offering a more sophisticated analysis than the traditional *k*-mer frequency method, which

simply counts the occurrence of nucleotide sequences without acknowledging their biochemical characteristics. Unlike the conventional method, this language model-based approach assesses sequences based on their contextual importance across different genetic environments, positioning contextually similar sequences close together in a multidimensional space. This technique provides deeper insights into the biochemical complexities of nucleotide sequences, thereby furnishing a more comprehensive understanding of an organism's functional biology [34]. To characterize the features of plasmids within the same MOB type, we employed language models to perform the MOB annotation. In addition to the relaxase-coding gene, language models exhibit the ability to capture more biological features and associations within comparable mobilization systems, making it possible to perform MOB annotation for metagenomic plasmid assemblies.

Thus, we presented MOBFinder, a tool for annotating MOB types from plasmid metagenomic fragments (PMFs). MOBFinder can process single or multiple plasmid DNA sequences, and provides predicted MOB types for each input fragment, including MOBB, MOBC, MOBF, MOBH, MOBL, MOBM, MOBP, MOBQ, MOBT, MOBV and non-MOB. Moreover, it provides the option to annotate plasmid bins from metagenomics data.

An overview of this work is shown in Figure 1A, and the development of MOBFinder involved the following steps: (1) Benchmark dataset construction. Plasmid complete genomes obtained from the National Center for Biotechnology Information (NCBI) were classified into different MOB types based on relaxase databases. Then, to simulate plasmid fragments in metagenomic data, an artificial benchmark dataset of varying lengths is generated. (2) Word embeddings. Numerical word vectors were generated using skip-gram to characterize the sequence features of different MOB categories. (3) Classification model ensemble and optimization. Several classification models, specifically designed for different lengths, were trained and integrated to predict fragments of different lengths. Evaluations against a test dataset demonstrated that MOBFinder is a powerful tool for MOB typing of plasmid fragments and bins. Its application to a cohort of patients with type II diabetes (T2D) revealed a potential correlation between some MOB types and the spread of antibiotic resistance genes among T2D patients. This suggests that MOBFinder is an effective data analysis approach for investigating plasmid-mediated horizontal gene transfer within microbial communities.

2. Materials and methods

2.1. The workflow of MOBFinder

To annotate the MOB type of plasmid fragments in metagenomics, we designed MOBFinder (Figure 1). As MOB-suite [19, 20] didn't offer a quantitative likelihood score for the outcomes and some plasmids would be classified into multiple MOB types (Figure S1), we constructed a benchmark dataset using a high-resolution MOB typing strategy for categorizing complete plasmid genomes (Figure 1B, 1C). Then, based on a language model and random forest, we designed an algorithm to perform MOB typing for PMFs (Figure 1D, 1E).

Figure 1. Flowchart of the technical approach utilized in this study. (A) General workflow of the development and testing of MOBFinder. (B) Using plasmid relaxases with known MOB types as reference sequences, we developed a database of relaxases from the non-redundant (NR) database representing different MOB types. (C) Utilizing the relaxase database, complete plasmid genomes from the NCBI were subjected to MOB typing. (D) Those complete genomes were also used to train a 4-mer language model using the skip-gram algorithm, allowing each 4-mer to be represented by a 100-dimensional word vector. For a DNA fragment, the average word vector of all 4-mers on its sequence serves as the feature vector for that DNA. (E) We constructed simulated metagenomic contigs from the complete genomes that had been MOB typed as a benchmark and encoded these contigs into word vectors. Then these word vectors were used to train a random forest algorithm. Then the trained model, with metagenomic DNA fragments as input, was used to predict the MOB typing of the corresponding DNA fragment based on its word vectors.

2.2. MOB typing of complete plasmid genomes

Traditionally, plasmid MOB typing of complete plasmid genomes has been a bioinformatics task based on the analysis of relaxase sequence similarity. The practice of annotating MOB types through BLAST similarity searches using representative sequences of different MOB type relaxases has gradually evolved into the standard method for MOB typing [4, 19, 20]. In this work, we constructed a benchmark dataset of simulated metagenomic contigs based on complete plasmid genomes with known MOB types. Previous studies have included a relatively small number of plasmids in their

analyses. To further expand the MOB typing training dataset, we annotated the newly collected plasmid complete genome data for MOB typing according to relaxase information.

Ten validated MOB relaxase protein families were collected, including MOBB, MOBC, MOBF, MOBH, MOBL, MOBM, MOBP, MOBQ, MOBT and MOBV [7-10, 35, 36] (Figure 1B). For each MOB category, blastp (RRID:SCR_001010) [37] was used to search homologous protein sequences against the NCBI non-redundant protein sequence database, with an *e-value* threshold of 1e-10, a *query coverage* threshold of 70%, and an *identity* threshold of 70%. A previous study applied an *e-value* threshold of 1e-5, and minimum requirements for *query coverage* and *identity* set at 50% [4]. However, employing these criteria, we observed that some relaxases were annotated as belonging to multiple MOB types. To eliminate ambiguous annotations and construct a more reliable dataset for the training of MOBFinder, we imposed the stricter criteria mentioned above. After the expansion of protein sequences, local relaxase databases were built using the 'makeblastdb' command for MOB typing of plasmid genomes.

Plasmid genomes were retrieved from the NCBI nucleotide database using the keywords 'complete' and 'plasmid,' and incomplete fragments were removed manually for further analysis. The accession list of these plasmids is provided in Supplementary Table 1. For each plasmid genome, coding sequences were extracted from the genebank file, and blastp [37] was employed to search for the best alignment of local relaxase databases. Here, we defined the *mob_score* to measure the likelihood of homology:

$$mob\_score = \sqrt{0.01 * qcov\_max * (1 - 1/\log10(bitscore\_max))}$$

where *qcov_max* and *bitscore_max* represent the *query coverage* and *bitscore* corresponding to the match with the highest bit score, respectively. To identify plasmid genomes encoding known relaxase families, we set a *mob_score* threshold of 0.5, which was established in conjunction with a minimum *query coverage* of 50% and a minimum *bitscore* of 100. To further enhance the reliability of our classification, we introduced an *e-value* cutoff, conservatively set at 1e-10, to complete the plasmid genome classification (Figure 1C). In instances where plasmid genomes yielded no blast results or exhibited an *e-value* exceeding 0.01, we categorized them as non-MOB.

2.3. Word embeddings using a language model

To characterize the features and patterns within each MOB category and use numerical word vectors to represent them, we utilized a skip-gram language model [30, 31] to learn from plasmid genomes. Using a sliding window, the model calculated the likelihood between segmented words and outputted a probability distribution over the context words. The training steps were as follows (Figure 1D):

(1) Word generation. Since DNA sequences are composed of different nucleotide characters, we used a *k*-mer sliding window to generate overlapping input words. For example, with k=4， 'ATCGCTGA' would be segmented into 'ATCG,' 'TCGC,' 'CGCT,' 'GCTG,' and 'CTGA'. In this step, unique words were generated.

(2) Word encoding initialization. Each word was initially assigned a random vector.

(3) Skip-gram model. We employed a standard skip-gram model as described in previous studies [30, 31] to generate word vectors through the dna2vec module [31]. A two-layer neural network was used to construct the skip-gram model. The initialized vectors were used as input, and the output was a probability distribution over the input words. Layer 1 was a hidden layer to convert the initialized vectors into a 100-dimensional word vector representation as predefined by Ng [31]. Layer 2 was used to compute and maximize the probability of the correct context words using the negative sampling function, with the size of context words set to 20 (10 words for upstream and downstream, respectively) as pre-set by Ng [31].

(4) Model training. For each input plasmid genome, we used an optimization algorithm to minimize the loss function. Then, using the default settings, we used backpropagation to update the neural network parameters (word vectors) for 10 epochs.

(5) Word vector extraction. After the training process, the word vectors in the hidden layer were extracted to characterize the plasmid fragments.

2.4. Benchmark dataset construction

Because there are no real metagenomic data to serve as a benchmark, using simulated data as a benchmark dataset is a common approach when developing bioinformatics tools [16, 24]. Therefore, in the development of MOBFinder, we artificially generated simulated datasets through the following steps:

(1) For classified plasmid genomes in each MOB category, we randomly split them at a proportion of 70% and 30% to construct the training and test datasets.

(2) Training dataset. To predict plasmid fragments with different lengths, we generated contigs of different length ranges: 100-400 bp, 401-800 bp, 801-1200 bp, and 1201-1600 bp. For each MOB class in each length range, we randomly generated 90000 artificial contigs. Plasmid fragments longer than 1600 bp were segmented into shorter contigs and predicted using models designed for the corresponding lengths.

(3) Test dataset. Because some plasmid fragments in real metagenomics datasets were much longer, we generated four length groups to assess the performance of MOBFinder: Group A with a length range of 801-1200 bp, Group B with a length range of 1201-1600 bp, Group C with a length range of 3000-4000 bp, and Group D with a length range of 5000-10000 bp. For each MOB class in these four groups, 500 fragments were randomly extracted.

2.5. Classification algorithm

To efficiently handle the training dataset and improve the robustness of MOBFinder, we employed random forest to train four predictive models using the training dataset. The detailed steps are as follows (Figure 1E):

(1) Word representation calculation. For each contig in the training dataset, we used a 4-mer sliding window to generate overlapping words and transformed them into numerical word vectors using trained word embeddings. To characterize the underlying features and patterns of the input contigs, we summed all the word vectors to compute their average as input of random forest.

(2) Classification model training. To improve the performance of MOBFinder, we trained four classification models on different lengths in the training dataset: 100-400 bp, 401-800 bp, 801-1200 bp, and 1201-1600 bp. The number of trees was set to 500 to generate predictive models.

(3) Model ensemble. The four trained models were ensembled into MOBFinder to make more accurate predictions. For fragments shorter than 100 bp, we used a model designed for 100-400 bp to predict the MOB type. For those longer than 1600 bp, we segmented them into short contigs and made predictions using the corresponding model. For example, a fragment with a length of 4000 bp would be segmented into three contigs: two with a length of 1600 bp and one of 800 bp. After predicting fragments with the corresponding models, we aggregated and calculated the weighted average scores for each MOB class, and the MOB type with the highest score was selected as the final prediction result for the input fragment.

(4) Plasmid bin classification. Metagenomic binning is an essential step in the reconstruction of genomes from individual microorganisms. Thus, we designed MOBFinder to perform MOB typing on both plasmid contigs and plasmid bins. If the input is a plasmid bin, MOBFinder predicts the likelihood of each MOB class for fragments within the bin. For each MOB category, MOBFinder aggregates the scores of each sequence within the bin and calculates the weighted average scores based on the sequence length. The MOB category with the maximum score is selected as the prediction result.

2.6. Performance validation

A test dataset was used to assess the performance of MOBFinder and compare it to MOB-suite and MOBscan. Because MOBscan can only predict MOB type using plasmid protein sequences rather than DNA sequences, we first annotated the proteins in the plasmid fragments of the test set using Prokka (RRID:SCR_014732) [38] and then used MOBscan to predict the MOB type based on the annotated proteins. We calculated overall *accuracy*, *kappa*, and *run time* by comparing the predicted classes and true classes. We used the online server of MOBscan to perform the MOB annotation, and the calculation of *run time* for MOBScan was confined to the duration spent on preprocessing

with Prokka locally. The overall *accuracy* was the proportion of accurate predictions. The *kappa* (a) was calculated to assess the overall consistency between the predictions and true classes, which took into account the possibility of random prediction. *Po* represented observed accuracy [$Po = (A_{11} + A_{22} + ... + Ann) / N$], where A_{11}, A_{22}, and *Ann* represented the values on the diagonal of the confusion matrix and *n* represented the number of MOB categories. *N* represented the total number of samples. *Pe* represented the expected accuracy [$Pe = (E_{11} + E_{22} + ... + Enn) / N$^2], where E_{11}, E_{22}, and *Enn* were the expected values in each cell of the confusion matrix, *n* was the number of MOB classes, and *N* was the total number of samples. The *run time* was recorded using the command 'time' in Linux.

$$kappa = (Po - Pe)/(1 - Pe) \quad (a)$$

$$balanced\ \ accuracy = (TPR + TNR)/2 \quad (b)$$

$$harmonic\ \ mean = 2 * Sn * Sp/(Sn + Sp) \quad (c)$$

$$F1 - score = 2 * precision * recall/(precision + recall) \quad (d)$$

For each MOB category, we also calculated the *balanced accuracy* (b), *harmonic mean* (c) and *F1-score* (d). Considering the class imbalance within the training dataset, *balanced accuracy* was used to measure the average accuracy of each MOB category, where *TPR* was the true positive rate [*TRP* = true positives / (true positives + false negatives)] and *TNR* was the true negative rate [*TNR* = true negatives / (true negatives + false positives)]. The *harmonic mean* provided an overall evaluation of the model's performance, where *Sn* and *Sp* represented sensitivity [*Sn* = true positives/ (true positives + false negatives)] and specificity [*Sp* = true negatives / (true negatives + false positives)], respectively. The *F1-score* combined *precision* and *recall*, providing a balanced measure of the model's performance, where *precision* was the number of correct positive predictions out of all positive predictions [*precision* = true positives / (true positives + false positives)] and *recall* was the number of correct positive predictions out of all actual positive predictions. [*recall* = true positives / (true positives + false negatives]].

A receiver operating characteristic (ROC) curve was used to visualize the performance of MOBFinder in predicting each MOB category, where the x-axis and y-axis were the false positive rate (*FPR*) and true positive rate (*TPR*). Plots closer to the left and top indicate higher *TPR* and lower *FPR*, which means better performance. For each MOB class, the area under the curve (AUC)

value was calculated to quantify the performance of MOBFinder. An AUC value between 0.5 and 1 indicates that the model performs better than random chance, and a higher AUC value indicates better prediction capability.

2.7. Annotation and analysis of T2D metagenomic data

Metagenomic sequencing data (SRA045646) were retrieved from the NCBI short read archive (SRA) database to investigate whether the plasmids within different MOB classes were associated with antibiotic resistance enrichment in T2D patients, as suggested by previous studies [39, 40]. All metagenomic data were preprocessed using the same protocols. PRINSEQ (RRID:SCR_005454) [41] was used to remove low-quality reads and bowtie2 (RRID:SCR_016368) [42] was used to remove host reads by aligning them to the human GRCH38 reference genome downloaded from the ENSEMBL database. We excluded metagenomic samples that did not pass quality control. Because the abundance of plasmids in metagenomes was much lower than that of bacteria, we only retained samples with more than 10,000,000 paired-end reads for downstream analysis (Supplementary Table 2).

To improve the efficiency and accuracy of assembly, we used MEGAHIT (RRID:SCR_018551) [43] to generate metagenomic contigs. PPR-Meta (RRID:SCR_016915) [24] was utilized to identify and extract plasmid fragments from the assembled fragments while filtering out bacteria and phage sequences. COCACOLA [44] was employed to cluster plasmid fragments into bins based on sequence similarity and composition. This allowed us to investigate the plasmid fragments from same originate and enabled better annotation and analysis of their functions.

MOBFinder was applied to annotate the MOB types in each plasmid bin. The average fragments per kilobase per million of each plasmid bin was calculated using bowtie2 to represent its abundance. Next, we analyzed the significance of differences in plasmid bins and various MOB types between healthy and T2D groups using the Wilcoxon rank-sum test. The calculation of *p* values was adjusted for multiple comparisons using the Benjamini-Hochberg method (denoted as *p.adjust*). ABRicate (RRID:SCR_021093) [45] was utilized to annotate antibiotic resistance genes (*identity*>50% and *qcov*>50%) in each plasmid bin, based on four antibiotic resistance gene databases [46-49]. The

Tukey's Honest Significant Difference test was performed to compare the identified resistance genes among different MOB classes. All statistical analyses were conducted using R.

3. Results

3.1. MOB typing of plasmid genomes

To construct the benchmark datasets, we obtained 90,395 complete plasmid genomes and categorized them into 11 MOB categories using blast (Table 2). We removed 22,470 of them potentially classified into more than one MOB class, leaving 67,925 classified genomes for the training and optimization of MOBFinder (Figure 2A). Our analysis results revealed significant differences in the number, average length, and GC content of plasmid genomes among MOB types. Notably, non-MOB types included the genomes with the most and longest average length, whereas MOBB and MOBM had the fewest plasmid genomes and shortest average length, respectively. In terms of GC content, MOBL had the lowest and MOBQ had the highest amounts. Moreover, plasmids of different MOB types exhibited diverse host ranges at the genus level (Figure 2B). MOBB was predominantly found in *Bacteroides*, *Hymenobacter*, *Parabacteroides*, *Phocaeicola* and *Spirosoma*. Particularly, *Phocaeicola* has been detected in the human gut and possessed the gene for porphyran degradation through horizontal gene transfer [50]. MOBC, MOBF, MOBH, and MOBP were all found in *Escherichia* and *Klebsiella*. And *Klebsiella* is a multidrug-resistant bacterium that has demonstrated resistance to multiple antibiotics [51]. MOBL, MOBT, and MOBV were mainly discovered in *Bacillus* and *Enterococcus*. Almost all MOBM type plasmid genomes were present in *Clostridium* and *Enterocloster*, and some species in *Clostridium* could cause various diseases [52]. MOBQ demonstrated a broader host range, including *Acinetobacter*, *Agrobacterium*, *Escherichia*, *Rhizobium*, *Lactiplantibacillus*, and *Staphylococcus*. Non-MOB plasmids were detected in the majority of bacteria. These results illustrate the relationship between different MOB types and their host ranges, and also demonstrate that MOB typing of plasmid fragments is feasible in the absence of relaxases.

Table 2. Number, average length, and GC content of plasmid genomes for each MOB type.

Figure 2. Benchmark dataset construction using a high-resolution strategy. (A) Proportion of

classified plasmid genomes. A confidence level of 'sure' means that the classified plasmid genomes had a *mob_score* of more than 0.5 and an *e-value* of less than 1e-10, while 'possible' did not. Plasmid genomes identified as 'sure' were used to generate benchmark datasets. Non-MOB, non-mobilizable plasmid. (B) Host range of the classified plasmid genomes at the genus level. Different colors represent different genera, and genera accounting for less than 5% of the total abundance are grouped under the category 'other.'

3.2. Overall performance of MOBFinder

We evaluated the overall performance of MOBFinder in terms of *accuracy*, *kappa*, and *run time*, and compared the tool to MOBscan and MOB-suite. MOBscan did not perform well, achieving low *accuracy* and *kappa* values across sequences of varying lengths, while MOB-suite exhibited marginally better performance than MOBscan when handling sequences of greater length (Figure 3A, 3B). In comparison, the *accuracy* of MOBFinder ranged from 70% to 77%, a significant improvement of at least 59% over MOB-suite (Figure 3A). The *kappa* of MOBFinder ranged between 67% and 75% and was approximately 65% higher than that of MOB-suite (Figure 3B). Moreover, MOBFinder exhibited a shorter *run time* in the test dataset, with a more gradual increase trend (Figure 3C). In general, these results indicate that MOBFinder greatly outperformed the other tools, and consistently improved in accuracy and consistency as the sequence length increased.

Figure 3. Overall performance of MOBFinder and comparison to MOB-suite and MOBScan. Evaluation and comparison in terms of (A) *accuracy*, (B) *kappa*, and (C) *run time* (C). The four fragment length groups in the test dataset were Group A (801-1200 bp), Group B (1201-1600 bp), Group C (3000-4000 bp), and Group D (500-10000 bp). (D) For each MOB type, the *balanced accuracy*, *harmonic mean*, and *F1-score* were used to assess the performance of MOBFinder and compared to MOB-suite and MOBscan. Since MOB-suite and MOBscan do not include the prediction of MOBL, only the results of MOBL from MOBFinder are provided. MOBFinder, MOB-suite and MOBscan are represented by blue lines, orange lines and gray lines respectively.

3.3. Evaluation by MOB category

Next, to evaluate the discrimination ability of MOBFinder for each MOB type, we calculated the

balanced accuracy, *harmonic mean*, and *F1-score* using the test dataset (Figure 3D). It demonstrated the highest performance for MOBB and MOBM, while its ability to identify non-MOB types was comparatively low. For MOBM, the *balanced accuracy* and *harmonic mean* reached up to 99% and the *F1-score* exceeded 96% for all length groups. For non-MOB, the *balanced accuracy* was 65%, the *harmonic mean* was 49%, and the *F1-score* was 40%. Compared to MOB-suite, MOBFinder exhibited much better performance in predicting all MOB classes. Even for non-MOB, it showed an approximate 13% improvement over the other tools in terms of *balanced accuracy*, 34% in terms of *harmonic mean*, and 24% in terms of *F1-score*.

In AUC analyses (Figure 4), all values were greater than 0.8, indicating that the tool effectively distinguished between positive and negative samples in each MOB class. In fact, most values were higher than 0.9, except for MOBT and non-MOB. The performance differences by MOB type might be attributable to the differences in host ranges and sequence features among types. Additionally, the imbalance in the training dataset for each MOB type may also be a primary factor contributing to the performance disparities.

Figure 4. ROC curves and AUC values for MOBFinder. The curves were plotted using the output scores of MOBFinder, and the AUC values were calculated to quantify the performance of the tool for each MOB class.

3.4. Application to T2D metagenomic data

In a previous study, enrichment analysis of fecal samples identified antibiotic resistance pathways in patients with T2D [40]. The precise mechanism of this enrichment, however, remained elusive. We used MOBFinder to analyze real T2D metagenomic data [39]. After preprocessing and assembly, 2,217,064 metagenomic fragments were generated, and plasmid assemblies were identified using PPR-Meta. Subsequently, the plasmid fragments were clustered into 55 bins and annotated using MOBFinder. By employing MOBFinder, we assigned 2 bins to the MOBF class, 8 bins to MOBL, 17 bins to MOBQ, and identified 28 bins as non-MOB (Figure 5A). Furthermore, we detected 15 bins that exhibited significant differences between the T2D group and a control group. Among them, 1 bin was classified as MOBF, 2 as MOBL, 5 as MOBQ, and 7 as non-MOB (Figure S2). Among

above MOB types, MOBQ contains the highest number of bins enriched in T2D, while MOBF is widely present in *Escherichia* and *Klebsiella* (Figure 2B), and that some strains of *Klebsiella* are resistant to multiple antibiotics, including carbapenems [53], these two MOB types might contribute to antibiotic resistance in T2D patients. Indeed, when we compared the average abundance of each MOB type between the T2D group and the control group (Figure 5B), the abundances of MOBF and MOBQ were significantly greater in the T2D group.

Figure 5. Annotation of T2D-related plasmid bins using MOBFinder. (A) Heatmap of plasmid bins between T2D patients and controls. Each column represents a sample, and each row represents a plasmid bin. (B) Comparison of the abundance of the four identified MOB types between T2D patients and controls. The *p-value* was calculated using the Wilcoxon rank-sum test, adjusted using the Benjamini-Hochberg method for multiple comparisons. (**p.adjust* < 0.05, ***p.adjust* < 0.01, and ****p.adjust* < 0.001.)

In addition, these two MOB types can be transferred among multiple bacterial species. This suggests that an increase in these two MOB types could potentially raise the risk of bacterial infection among individuals with T2D. Subsequently, we used four databases [46-49] to detect drug resistance genes in four MOB types (Figure 6). The number of such genes was significantly higher in MOBF than in the other three MOB types. This suggests that MOBF plasmids may carry more drug resistance genes than the other MOB types. Furthermore, the increase in MOBF and MOBQ plasmids could result in more bacteria acquiring drug resistance genes, thereby leading to more antibiotic resistance pathways in T2D patients. In summary, our results demonstrate the utility of MOBFinder for annotating plasmid fragments in metagenomes, uncovering the potential mechanisms underlying the antibiotic resistance enrichment in metagenomic analysis.

Figure 6. Comparison of resistance genes among different MOB types. Four databases were used to identify antibiotic resistance gene within each MOB type, and the *p-value* was calculated using Tukey's Honest Significant Difference test. The two groups without significance markings indicate no statistical difference. (**p-value* < 0.05, ***p-value* < 0.01 and ****p-value* < 0.001.)

3.5. Use of MOBFinder

MOBFinder can predict the MOB type of plasmid fragments and bins in metagenomics. For PMFs, it takes a FASTA file as input. The output file consists of 13 columns. The first column represents the fragment ID, the second column displays the predicted MOB type, and columns 3 to 13 represent the scores for each MOB class, namely MOBB, MOBC, MOBF, MOBH, MOBL, MOBM, MOBP, MOBQ, MOBT, MOBV, and non-MOB.

For plasmid metagenomic bins, MOBFinder requires two input files: a FASTA file containing the plasmid fragments and a meta table that records the mapping between plasmid fragment IDs and bin IDs. The output results are similar to those of plasmid fragments. The first column is the plasmid bin ID. The second is the predicted MOB class of the plasmid bins. The other columns present the MOB scores of the different MOB types.

4. Discussion

We developed MOBFinder based on a language model and the random forest algorithm to classify plasmid fragments and bins from metagenomics data into MOB types. First, using the relaxase-alignment method, plasmid genomes were classified into distinct MOB categories. Analyses revealed substantial differences in parameters such as the number, average length, and GC content of plasmid genomes across MOB types. Additionally, there were noteworthy differences in the host ranges among different MOB classes. These results suggest the potential of utilizing sequence features from different MOB types for PMF MOB typing. To characterize the plasmids within each MOB type, we used the skip-gram model to generate word vectors. Our tool demonstrated superior overall performance compared to other tools. Specifically, for each MOB category, MOBFinder exhibited significant improvements in *balanced accuracy*, *harmonic mean*, and *F1-score*, with values reaching up to 99% for the first two measures in the MOBM category.

Traditionally, *k*-mer frequency models and one-hot encoding have commonly been employed to digitize biological sequences, extensively applied across various machine learning algorithms [54]. However, both models simply mark or count the frequency of various characters in sequences, failing to reflect the biological significance underlying each character. These models may also

encounter dimensionality issues [54]. For instance, in the *k*-mer model, if *k* is set to 8, the dimensionality of the *k*-mer vector of each DNA sequence becomes 4^8, which is problematic in metagenomics where most fragment lengths do not reach this magnitude. This would result in significant noise in the feature vector and cause overfitting. Similarly, in the one-hot model, for a sequence of length *L* using 4-mers as the base unit, it would require *L* one-hot vectors each with a dimensionality of 4^4. In such instances, if the dataset for training is not sufficiently large, this representation method could also lead to overfitting due to high dimensionality. In contrast, word vector models offer a superior solution to these problems. Such models initially perform a random initialization of vectors for each "word." Taking the skip-gram algorithm utilized in this study as an example, the dimension of a random vector can be 1-of-*n*, where *n* represents the size of the vocabulary [30]. Following unsupervised pre-training on large datasets, the algorithm maps characters with similar contexts to similar feature spaces. The dimensions of the coordinates (i.e., the word vectors) of these feature spaces will be lower than those of the initial random vectors. Thus, through unsupervised pre-training on large datasets, language models can compress high-dimensional initial vectors into lower-dimensional word vectors (e.g., MOBFinder's word vectors have a dimensionality of 100), enabling the feature vectors to contain more character information while effectively avoiding dimensionality issues during supervised training.

In a metagenomic sequences classification task, 4-mer is widely used as the basic unit in various bioinformatics tools [55], thus MOBFinder takes this as a "word." To assess the impact of training word vectors with different *k*-mer lengths on performance, we compared models with *k*-mer lengths of 2, 3, 4, 5, 6, 7, and 8 (Figure S3). We observed lower overall *accuracy* and *kappa* values for *k*=2. At *k*=4, the *balanced accuracy*, *harmonic mean*, *F1-score*, and *AUC* values stabilized across different MOB types. Subsequently, as the *k*-mer length increased, there was no significant improvement in *accuracy* or other metrics, while the *run time* gradually increased. Therefore, we chose a *k*-mer length of 4 for training word vectors and developing MOBFinder.

Interestingly, in an analysis of T2D metagenomic sequencing data [39], we noted a significant increase in MOBF and MOBQ type plasmids in T2D patients. Moreover, we found more drug resistance genes in the MOBF class, whose dominant hosts are *Klebsiella* and *Escherichia*, which

are associated with the spread of multidrug resistance. Although previous analyses of gut metagenomic data from patients with T2D have reported enrichment of drug resistance pathways [40], our results suggest a potential reason for it: the increased abundance of MOBF and MOBQ type plasmids in the guts of individuals with T2D may disseminate more antibiotic resistance genes, resulting in such enrichment.

At present, databases contain a large amount of human metagenomic data derived from second-generation sequencing. However, understanding of the functions of numerous disease-linked microbial sequences remains limited, attributable to the incomplete nature of metagenomic fragments. The development of MOBFinder enables MOB annotation for plasmid fragments from metagenomics data and provides a powerful tool for investigating the transmission mechanisms of plasmid-mediated antibiotic resistance genes and virulence factors.

5. Conclusions

In summary, MOBFinder is a tool for MOB typing of plasmid fragments and bins from metagenomic data. Analyses of classified plasmid genomes unveiled notable differences in sequence characteristics and host ranges across MOB types. Hence, we employed a language model to extract the sequence features specific to each MOB type and represented them using word vectors. Additionally, we boosted prediction accuracy by training and integrating several random forest classification models. MOBFinder surpassed other tools in performance tests and successfully detected an increase in certain MOB type plasmids in T2D patients. Importantly, these MOB type plasmids harbor potential drug-resistance genes, thus offering an explanation for the observed antibiotic resistance in T2D individuals. This suggests that MOBFinder could potentially aid the formulation of specific medications to curb drug resistance transmission. We anticipate that MOBFinder will be a powerful tool for the analysis of plasmid-mediated transmission.

Availability of Source Code and Requirements

- Project name: MOBFinder
- Project homepage: https://github.com/FengTaoSMU/MOBFinder
- Operating system(s): Linux

- Programming language: Python, R script
- Other requirements: BLAST, biopython
- License: GPL-3.0
- RRID: SCR_024451
- biotoolsID: MOBFinder

Data Availability

Snapshots of our code and other data further supporting this work are openly available in the GigaScience repository, GigaDB [56].

Abbreviations

MOB: mobilization typing; Rep: replicon typing; Inc: incompatibility typing; MPF: mate-pair formation typing; non-MOB: non-mobilizable; T2D: type 2 diabetes; TPR: true positive rate; TNR: true negative rate; Sn: sensitivity; Sp: specificity; ROC: the receiver operating characteristic; AUC: the area under the curve; SRA: short read archive; NCBI: National Center for Biotechnology Information; PlasTax-PCR: Plasmid Taxonomic PCR; PBRT: PCR-based replicon typing; DPMT: degenerate primer MOB typing.

Competing Interests

The authors declare that they have no competing interests.

Funding

This investigation was financially supported by the National Key R&D Program of China (2022YFA0806400) and the National Natural Science Foundation of China (82102508, 81925026).

Author Contributions

TF, ZCF, and HWZ proposed and designed this work. TF and ZCF developed and optimized the software. TF, ZCF, SFW, and HWZ wrote and revised the manuscript.

Supplementary data

Supplementary Table 1. Accessions list of classified plasmid genomes.

Supplementary Table 2. List of metagenomic samples used in our analysis.

Supplementary Figure 1. MOB typing using MOB-suite. Single-class, plasmid genomes classified into one MOB type; multi-class, plasmid genomes classified into more than one MOB category; non-MOB, non-mobilizable plasmids.

Supplementary Figure 2. Abundance of each significantly different plasmid bin from various MOB types between patients with type II diabetes and controls.

Supplementary Figure 3. Comparison results for the development of MOBFinder using word vectors trained with different *k*-mer lengths. (A-C) Overall *accuracy*, *kappa*, and *run time* of the MOB classification model trained with word vectors trained using different lengths of *k*-mers. (D) *Balanced accuracy*, *harmonic mean*, *F1-score*, and AUC of word vectors trained with different *k*-mer lengths across different MOB types.

References

1. Helinski DR. A Brief History of Plasmids. EcoSal Plus. 2022 Dec 15;10(1):eESP00282021. doi: 10.1128/ecosalplus.esp-0028-2021
2. Garcillán-Barcia MP, Francia MV, de la Cruz F. The diversity of conjugative relaxases and its application in plasmid classification. FEMS Microbiol Rev. 2009 May;33(3):657-87. doi: 10.1111/j.1574-6976.2009.00168.x
3. Rodríguez-Beltrán J, DelaFuente J, León-Sampedro R, MacLean RC, San Millán Á. Beyond horizontal gene transfer: the role of plasmids in bacterial evolution. Nat Rev Microbiol. 2021 Jun;19(6):347-359. doi: 10.1038/s41579-020-00497-1
4. Shintani M, Sanchez ZK, Kimbara K. Genomics of microbial plasmids: classification and identification based on replication and transfer systems and host taxonomy. Front Microbiol. 2015 Mar 31;6:242. doi: 10.3389/fmicb.2015.00242
5. Redondo-Salvo S, Bartomeus-Peñalver R, Vielva L, Tagg KA, et al. COPLA, a taxonomic classifier of plasmids. BMC Bioinformatics. 2021 Jul 31;22(1):390. doi: 10.1186/s12859-021-04299-x

6. Carattoli A, Hasman H. PlasmidFinder and In Silico pMLST: Identification and Typing of Plasmid Replicons in Whole-Genome Sequencing (WGS). Methods Mol Biol. 2020;2075:285-294. doi: 10.1007/978-1-4939-9877-7_20
7. Smillie C, Garcillán-Barcia MP, Francia MV, Rocha EP, de la Cruz F. Mobility of plasmids. Microbiol Mol Biol Rev. 2010 Sep;74(3):434-52. doi: 10.1128/MMBR.00020-10
8. Francia MV, Varsaki A, Garcillán-Barcia MP, Latorre A, Drainas C, de la Cruz F. A classification scheme for mobilization regions of bacterial plasmids. FEMS Microbiol Rev. 2004 Feb;28(1):79-100. doi: 10.1016/j.femsre.2003.09.001
9. Garcillán-Barcia MP, Francia MV, de la Cruz F. The diversity of conjugative relaxases and its application in plasmid classification. FEMS Microbiol Rev. 2009 May;33(3):657-87. doi: 10.1111/j.1574-6976.2009.00168.x
10. Garcillán-Barcia MP, Alvarado A, de la Cruz F. Identification of bacterial plasmids based on mobility and plasmid population biology. FEMS Microbiol Rev. 2011 Sep;35(5):936-56. doi: 10.1111/j.1574-6976.2011.00291.x
11. Bradley P, den Bakker HC, Rocha EPC, McVean G, Iqbal Z. Ultrafast search of all deposited bacterial and viral genomic data. Nat Biotechnol. 2019 Feb;37(2):152-159. doi: 10.1038/s41587-018-0010-1
12. Alvarado A, Garcillán-Barcia MP, de la Cruz F. A degenerate primer MOB typing (DPMT) method to classify gamma-proteobacterial plasmids in clinical and environmental settings. PLoS One. 2012;7(7):e40438. doi: 10.1371/journal.pone.0040438
13. Garcillán-Barcia MP, Alvarado A, de la Cruz F. Identification of bacterial plasmids based on mobility and plasmid population biology. FEMS Microbiol Rev. 2011 Sep;35(5):936-56. doi: 10.1111/j.1574-6976.2011.00291.x
14. Cuartas R, Coque TM, de la Cruz F, Garcillán-Barcia MP. PLASmid TAXonomic PCR (PlasTax-PCR), a Multiplex Relaxase MOB Typing to Assort Plasmids into Taxonomic Units. Methods Mol Biol. 2022;2392:127-142. doi: 10.1007/978-1-0716-1799-1_10
15. Carattoli A, Bertini A, Villa L, Falbo V, Hopkins KL, Threlfall EJ. Identification of plasmids by PCR-based replicon typing. J Microbiol Methods. 2005 Dec;63(3):219-28. doi: 10.1016/j.mimet.2005.03.018
16. Fang Z, Zhou H. Identification of the conjugative and mobilizable plasmid fragments in the

plasmidome using sequence signatures. Microb Genom. 2020 Nov;6(11):mgen000459. doi: 10.1099/mgen.0.000459

17. Li X, Xie Y, Liu M, Tai C, Sun J, Deng Z, Ou HY. oriTfinder: a web-based tool for the identification of origin of transfers in DNA sequences of bacterial mobile genetic elements. Nucleic Acids Res. 2018 Jul 2;46(W1):W229-W234. doi: 10.1093/nar/gky352
18. Garcillán-Barcia MP, Redondo-Salvo S, Vielva L, de la Cruz F. MOBscan: Automated Annotation of MOB Relaxases. Methods Mol Biol. 2020;2075:295-308. doi: 10.1007/978-1-4939-9877-7_21
19. Robertson J, Nash JHE. MOB-suite: software tools for clustering, reconstruction and typing of plasmids from draft assemblies. Microb Genom. 2018 Aug;4(8):e000206. doi: 10.1099/mgen.0.000206
20. Robertson J, Bessonov K, Schonfeld J, Nash JHE. Universal whole-sequence-based plasmid typing and its utility to prediction of host range and epidemiological surveillance. Microb Genom. 2020 Oct;6(10):mgen000435. doi: 10.1099/mgen.0.000435
21. Krawczyk PS, Lipinski L, Dziembowski A. PlasFlow: predicting plasmid sequences in metagenomic data using genome signatures. Nucleic Acids Res. 2018 Apr 6;46(6):e35. doi: 10.1093/nar/gkx1321
22. Roosaare M, Puustusmaa M, Möls M, Vaher M, Remm M. PlasmidSeeker: identification of known plasmids from bacterial whole genome sequencing reads. PeerJ. 2018 Apr 2;6:e4588. doi: 10.7717/peerj.4588
23. Pellow D, Mizrahi I, Shamir R. PlasClass improves plasmid sequence classification. PLOS Comput Biol. 2020;16:e1007781. doi: 10.1371/journal.pcbi.1007781
24. Fang Z, Tan J, Wu S, Li M, Xu C, Xie Z, et al. PPR-Meta: a tool for identifying phages and plasmids from metagenomic fragments using deep learning. GigaScience. 2019;8:1–14. 10.1093/gigascience/giz066
25. Pradier L, Tissot T, Fiston-Lavier AS, Bedhomme S. PlasForest: a homology-based random forest classifier for plasmid detection in genomic datasets. BMC Bioinformatics. 2021 Jun 26;22(1):349. doi: 10.1186/s12859-021-04270-w
26. Sobecky PA, Hazen TH. Horizontal gene transfer and mobile genetic elements in marine systems. Methods Mol Biol. 2009;532:435-53. doi: 10.1007/978-1-60327-853-9_25

27. Suzuki H, Yano H, Brown CJ, Top EM. Predicting plasmid promiscuity based on genomic signature. J Bacteriol. 2010 Nov;192(22):6045-55. doi: 10.1128/JB.00277-10

28. Wu S, Fang Z, Tan J, Li M, Wang C, Guo Q, Xu C, Jiang X, Zhu H. DeePhage: distinguishing virulent and temperate phage-derived sequences in metavirome data with a deep learning approach. Gigascience. 2021 Sep 8;10(9):giab056. doi: 10.1093/gigascience/giab056

29. Fang Z, Feng T, Zhou H, Chen M. DeePVP: Identification and classification of phage virion proteins using deep learning. Gigascience. 2022 Aug 11;11:giac076. doi: 10.1093/gigascience/giac076

30. Mikolov T, Chen K, Corrado G, and Dean J. Efficient estimation of word representations in vector space. 2013. arXiv preprint. doi: arXiv:1301.3781.

31. Patrick Ng. dna2vec: Consistent vector representations of variable-length k-mers. arXiv. doi: 10.48550/arXiv.1701.06279

32. Tsukiyama S, Hasan MM, Fujii S, Kurata H. LSTM-PHV: prediction of human-virus protein-protein interactions by LSTM with word2vec. Brief Bioinform. 2021 Nov 5;22(6):bbab228. doi: 10.1093/bib/bbab228

33. Sharma R, Shrivastava S, Kumar Singh S, Kumar A, Saxena S, Kumar Singh R. Deep-ABPpred: identifying antibacterial peptides in protein sequences using bidirectional LSTM with word2vec. Brief Bioinform. 2021 Sep 2;22(5):bbab065. doi: 10.1093/bib/bbab065

34. Asgari E, Mofrad MR. Continuous Distributed Representation of Biological Sequences for Deep Proteomics and Genomics. PLoS One. 2015 Nov 10;10(11):e0141287. doi: 10.1371/journal.pone.0141287

35. Wisniewski JA, Traore DA, Bannam TL, Lyras D, Whisstock JC, Rood JI. TcpM: a novel relaxase that mediates transfer of large conjugative plasmids from Clostridium perfringens. Mol Microbiol. 2016 Mar;99(5):884-96. doi: 10.1111/mmi.13270

36. Ramachandran G, Miguel-Arribas A, Abia D, Singh PK, Crespo I, Gago-Córdoba C, Hao JA, Luque-Ortega JR, Alfonso C, Wu LJ, Boer DR, Meijer WJ. Discovery of a new family of relaxases in Firmicutes bacteria. PLoS Genet. 2017 Feb 16;13(2):e1006586. doi: 10.1371/journal.pgen.1006586

37. Camacho C, Coulouris G, Avagyan V, Ma N, Papadopoulos J, Bealer K, Madden TL. BLAST+: architecture and applications. BMC Bioinformatics. 2009 Dec 15;10:421. doi: 10.1186/1471-

2105-10-421

38. Seemann T. Prokka: rapid prokaryotic genome annotation. Bioinformatics. 2014 Jul 15;30(14):2068-9. doi: 10.1093/bioinformatics/btu153

39. Qin J, Li Y, Cai Z, Li S, Zhu J, Zhang F et al. A metagenome-wide association study of gut microbiota in type 2 diabetes. Nature. 2012 Oct 4;490(7418):55-60. doi: 10.1038/nature11450

40. Wu H, Tremaroli V, Schmidt C, Lundqvist A, Olsson LM, Krämer M, Gummesson A, Perkins R, Bergström G, Bäckhed F. The Gut Microbiota in Prediabetes and Diabetes: A Population-Based Cross-Sectional Study. Cell Metab. 2020 Sep 1;32(3):379-390.e3. doi: 10.1016/j.cmet.2020.06.011

41. Schmieder R, Edwards R. Quality control and preprocessing of metagenomic datasets. Bioinformatics. 2011 Mar 15;27(6):863-4. doi: 10.1093/bioinformatics/btr026

42. Langmead B, Salzberg SL. Fast gapped-read alignment with Bowtie 2. Nat Methods. 2012 Mar 4;9(4):357-9. doi: 10.1038/nmeth.1923

43. Li D, Liu CM, Luo R, Sadakane K, Lam TW. MEGAHIT: an ultra-fast single-node solution for large and complex metagenomics assembly via succinct de Bruijn graph. Bioinformatics. 2015 May 15;31(10):1674-6. doi: 10.1093/bioinformatics/btv033

44. Lu YY, Chen T, Fuhrman JA, Sun F. COCACOLA: binning metagenomic contigs using sequence COmposition, read CoverAge, CO-alignment and paired-end read LinkAge. Bioinformatics. 2017 Mar 15;33(6):791-798. doi: 10.1093/bioinformatics/btw290

45. Seemann T. Abricate. Github. https://github.com/tseemann/abricate

46. Gupta SK, Padmanabhan BR, Diene SM, Lopez-Rojas R, Kempf M, Landraud L, Rolain JM. ARG-ANNOT, a new bioinformatic tool to discover antibiotic resistance genes in bacterial genomes. Antimicrob Agents Chemother. 2014;58(1):212-20. doi: 10.1128/AAC.01310-13

47. Jia B, Raphenya AR, Alcock B, Waglechner N, Guo P, Tsang KK, Lago BA, Dave BM, Pereira S, Sharma AN, Doshi S, Courtot M, Lo R, Williams LE, Frye JG, Elsayegh T, Sardar D, Westman EL, Pawlowski AC, Johnson TA, Brinkman FS, Wright GD, McArthur AG. CARD 2017: expansion and model-centric curation of the comprehensive antibiotic resistance database. Nucleic Acids Res. 2017 Jan 4;45(D1):D566-D573. doi: 10.1093/nar/gkw1004

48. Doster E, Lakin SM, Dean CJ, Wolfe C, Young JG, Boucher C, Belk KE, Noyes NR, Morley PS. MEGARes 2.0: a database for classification of antimicrobial drug, biocide and metal

resistance determinants in metagenomic sequence data. Nucleic Acids Res. 2020 Jan 8;48(D1):D561-D569. doi: 10.1093/nar/gkz1010

49. Feldgarden M, Brover V, Haft DH, Prasad AB, Slotta DJ, Tolstoy I, Tyson GH, Zhao S, Hsu CH, McDermott PF, Tadesse DA, Morales C, Simmons M, Tillman G, Wasilenko J, Folster JP, Klimke W. Validating the AMRFinder Tool and Resistance Gene Database by Using Antimicrobial Resistance Genotype-Phenotype Correlations in a Collection of Isolates. Antimicrob Agents Chemother. 2019 Oct 22;63(11):e00483-19. doi: 10.1128/AAC.00483-19

50. Hehemann JH, Correc G, Barbeyron T, Helbert W, Czjzek M, Michel G. Transfer of carbohydrate-active enzymes from marine bacteria to Japanese gut microbiota. Nature. 2010 Apr 8;464(7290):908-12. doi: 10.1038/nature08937

51. Fu S, Wang R, Xu Z, Zhou H, Qiu Z, Shen L and Yang Q. Metagenomic sequencing combined with flow cytometry facilitated a novel microbial risk assessment framework for bacterial pathogens in municipal wastewater without cultivation. iMeta. 2023;2:e77 .doi:10.1002/imt2.77

52. Dieterle MG, Rao K, Young VB. Novel therapies and preventative strategies for primary and recurrent Clostridium difficile infections. Ann N Y Acad Sci. 2019 Jan;1435(1):110-138. doi: 10.1111/nyas.13958

53. Yang X, Dong N, Chan EW, Zhang R, Chen S. Carbapenem Resistance-Encoding and Virulence-Encoding Conjugative Plasmids in Klebsiella pneumoniae. Trends Microbiol. 2021 Jan;29(1):65-83. doi: 10.1016/j.tim.2020.04.012

54. Jaillard M, Palmieri M, van Belkum A, Mahé P. Interpreting k-mer-based signatures for antibiotic resistance prediction. Gigascience. 2020 Oct 17;9(10):giaa110. doi: 10.1093/gigascience/giaa110

55. Sedlar K, Kupkova K, Provaznik I. Bioinformatics strategies for taxonomy independent binning and visualization of sequences in shotgun metagenomics. Comput Struct Biotechnol J. 2016 Dec 5;15:48-55. doi: 10.1016/j.csbj.2016.11.005

56. Feng T; Wu S; Zhou H; Fang Z. Supporting data for "MOBFinder: A tool for mobilization typing of plasmid metagenomic fragments based on a language model" GigaScience Database 2024. https://doi.org/10.5524/102559

Table 1. Experimental and computational schemes developed for plasmid classification.

Technology category	Method	Classification scheme	Material	Description
Experimental	DPMT [12]	MOB typing	Plasmid DNA from clinical isolates	Used degenerate primers to hybridize relaxase-coding genes to identify and classify plasmids isolated from clinical isolates
	PlasTax-PCR [14]	Taxonomic typing	Plasmid DNA from clinical isolates	Utilized PCR primers that target conserved segments of the relaxase gene of plasmid taxonomic units (PTUs) to identify specific PTUs of transmissible plasmids
	PBRT [15]	Rep typing or Inc typing	Plasmid DNA from clinical isolates	Used multiplex PCR to amplify DNA fragments of replicons and detect known replicon types of plasmids
Computational	MOBscan [18]	MOB typing	Plasmid protein sequences	Used the HMMER model to annotate the relaxases and further perform MOB typing
	MOB-suite [19, 20]	MOB typing, MPF typing and Rep typing	Complete plasmid genomes or plasmid assembly clusters (Linux)	Utilized collected relaxase, oriT, replicon, and T4SS sequences to construct database, then classified plasmid assembly clusters with BLAST
	PlasTans [16]	transmissible plasmid identification	Plasmid assembly contigs (Linux)	Used the convolutional neural network deep learning algorithm to classify plasmid DNA fragments
	PlasmidFinder [6]	Rep typing or Inc typing	Raw reads or complete plasmid genomes or plasmid assembly contigs (web server)	Utilized collected replicon sequences and BLASTn to perform Rep typing and Inc typing
	pMLST [6]	Rep typing or Inc typing	Raw reads or complete plasmid genomes or plasmid assembly contigs (web server)	Used collected plasmid multilocus sequence typing (pMLST) allele sequences, known sequence type profiles, and BLAST to perform Rep typing and Inc typing
	oriTfinder [17]	MOB typing, MPF typing	Complete plasmid genomes (web server)	Utilized collected oriT, relaxase, T4CP, and T4SS sequences to annotate plasmids with BLAST

COPLA [5]	Taxonomic typing	Complete plasmid genomes or plasmid assembly sets (Linux)	Used average nucleotide identity (ANI) metrics and hierarchical stochastic block modeling (HSBM) to create plasmid taxonomic units (PTUs) and predict taxonomic hosts

Table 2. Number, average length, and GC content of plasmid genomes for each MOB type.

Class	Number	Average length	GC (%)
MOBB	623	10921.77	51.27
MOBC	3218	19965.28	47.14
MOBF	21268	103802.80	52.07
MOBH	4880	151108.10	48.37
MOBL	3446	51430.63	34.57
MOBM	1761	2684.14	27.12
MOBP	15617	32237.88	49.70
MOBQ	9347	89357.64	56.77
MOBT	1181	11643.24	36.92
MOBV	4405	6595.43	37.75
Non-MOB	24649	37581.85	49.84

Figure 1

Click here to access/download;Figure;Figure1.tif

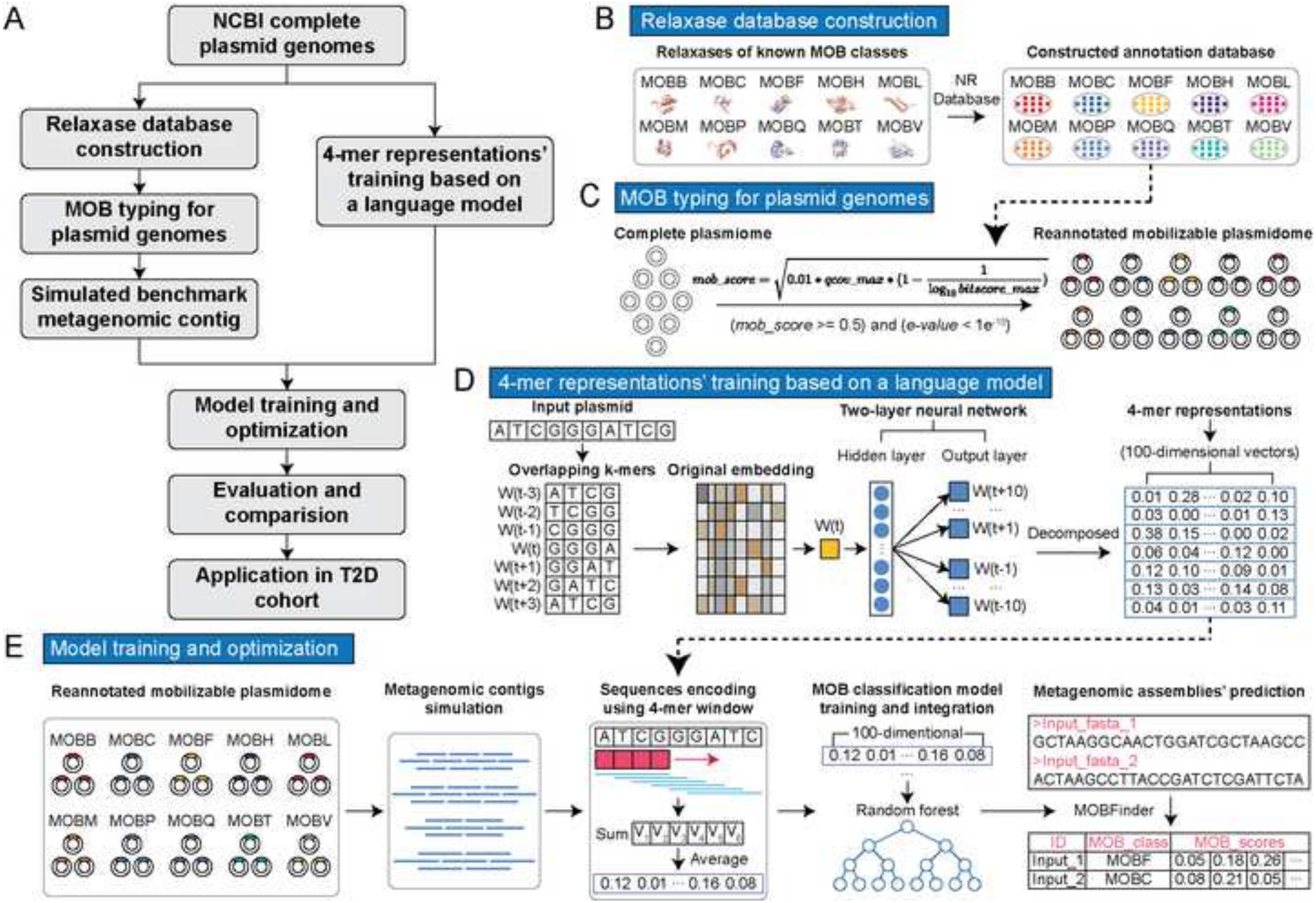

Figure 2

Click here to access/download;Figure;Figure2.tif

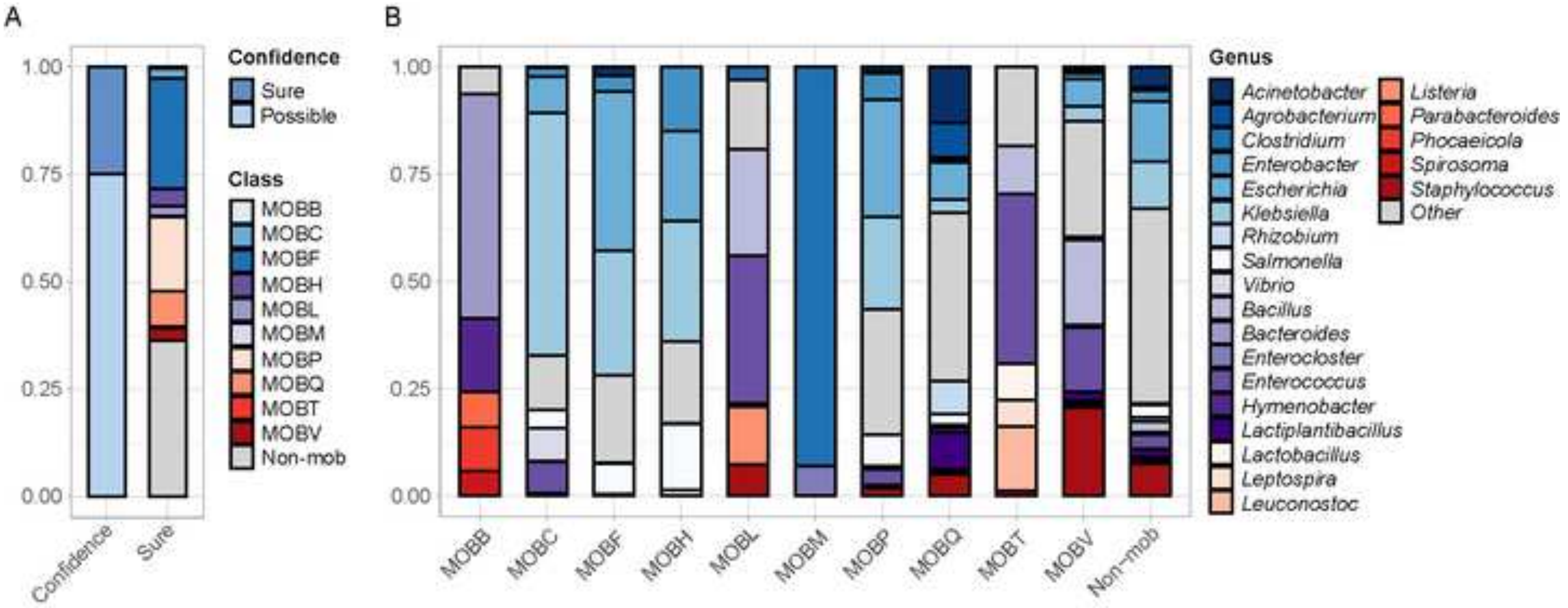

Figure 3

Click here to access/download;Figure;Figure3.tif

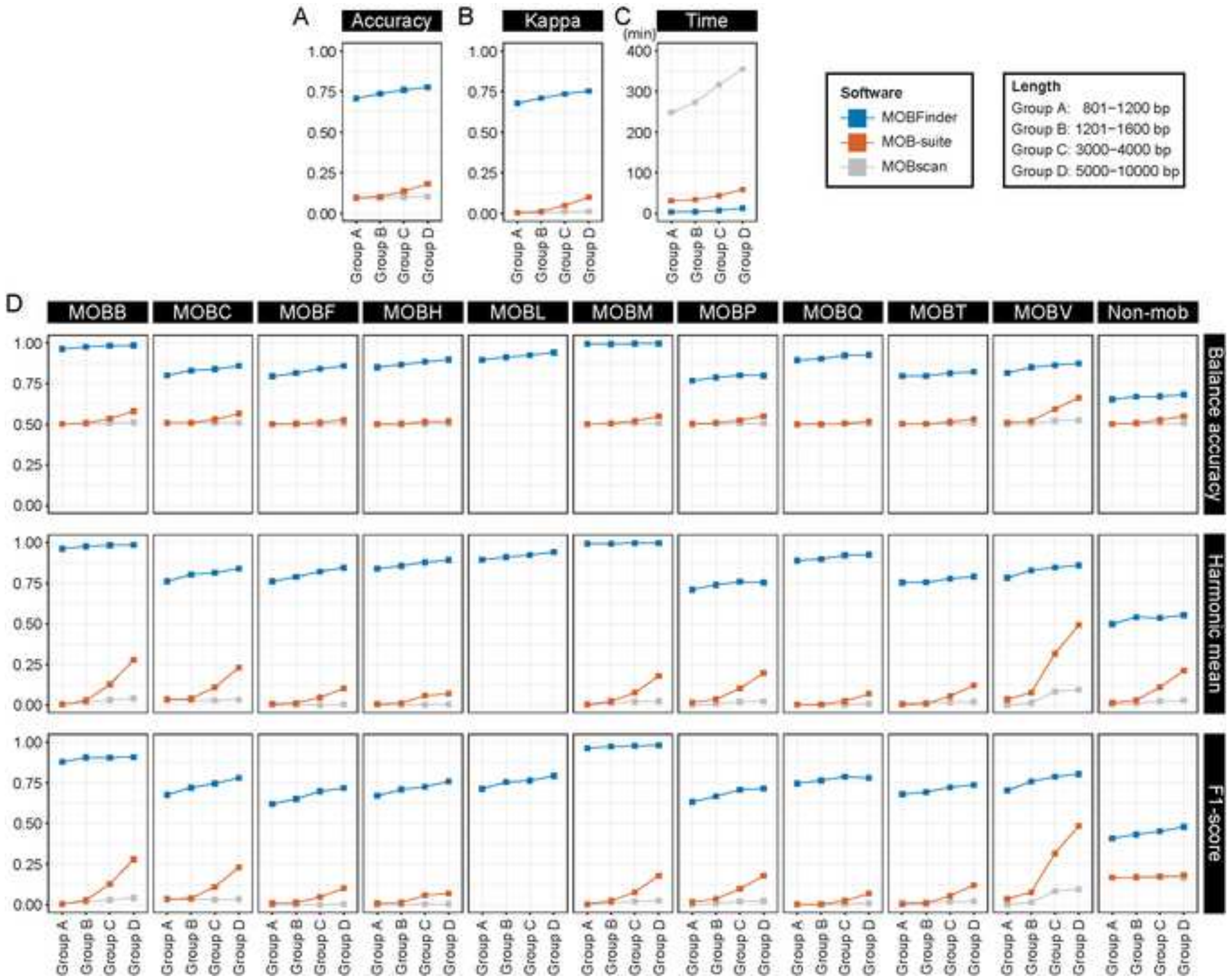

Figure 4

Click here to access/download;Figure;Figure4.tif

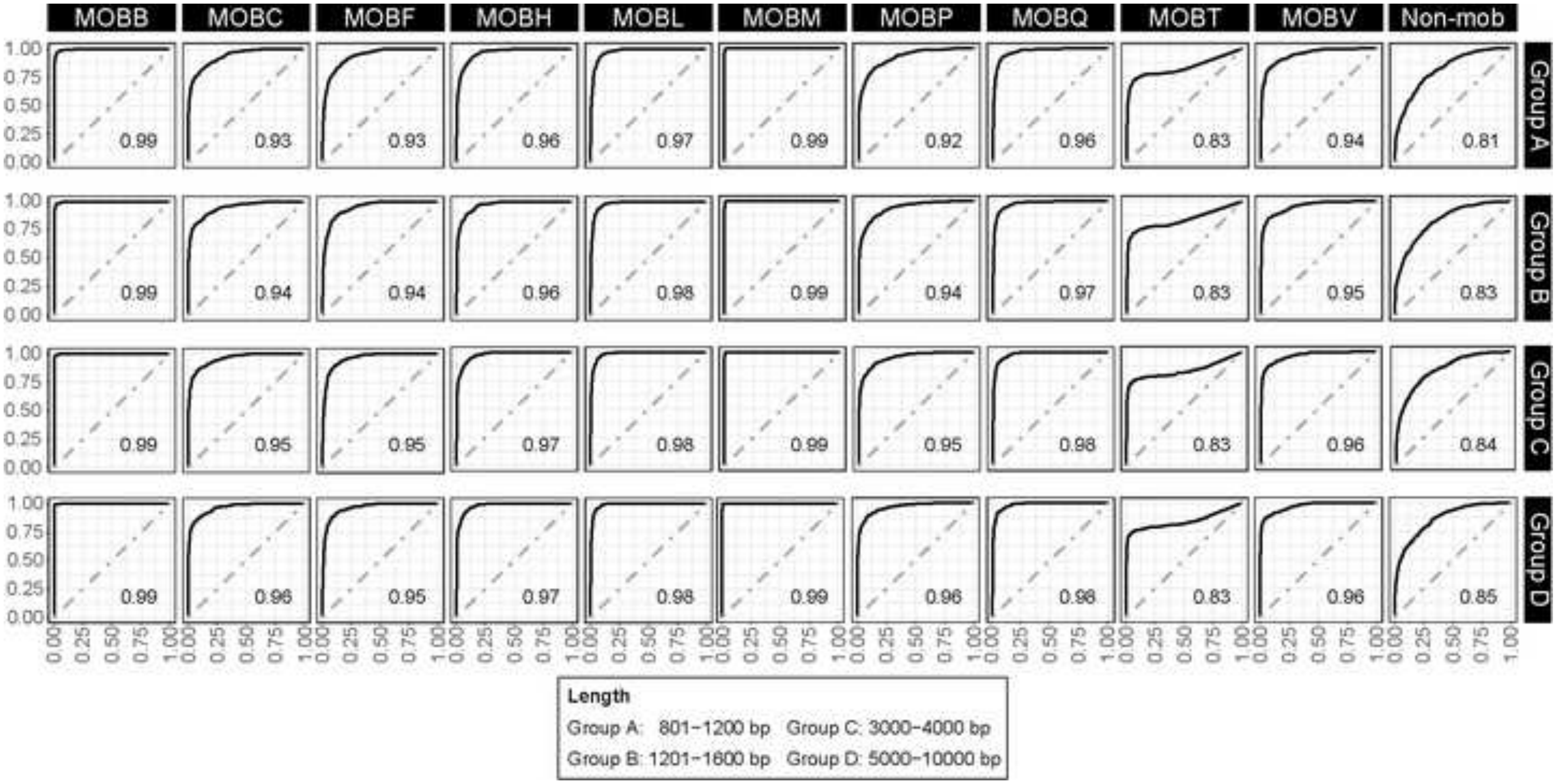

Figure 5

Click here to access/download;Figure;Figure5.tif

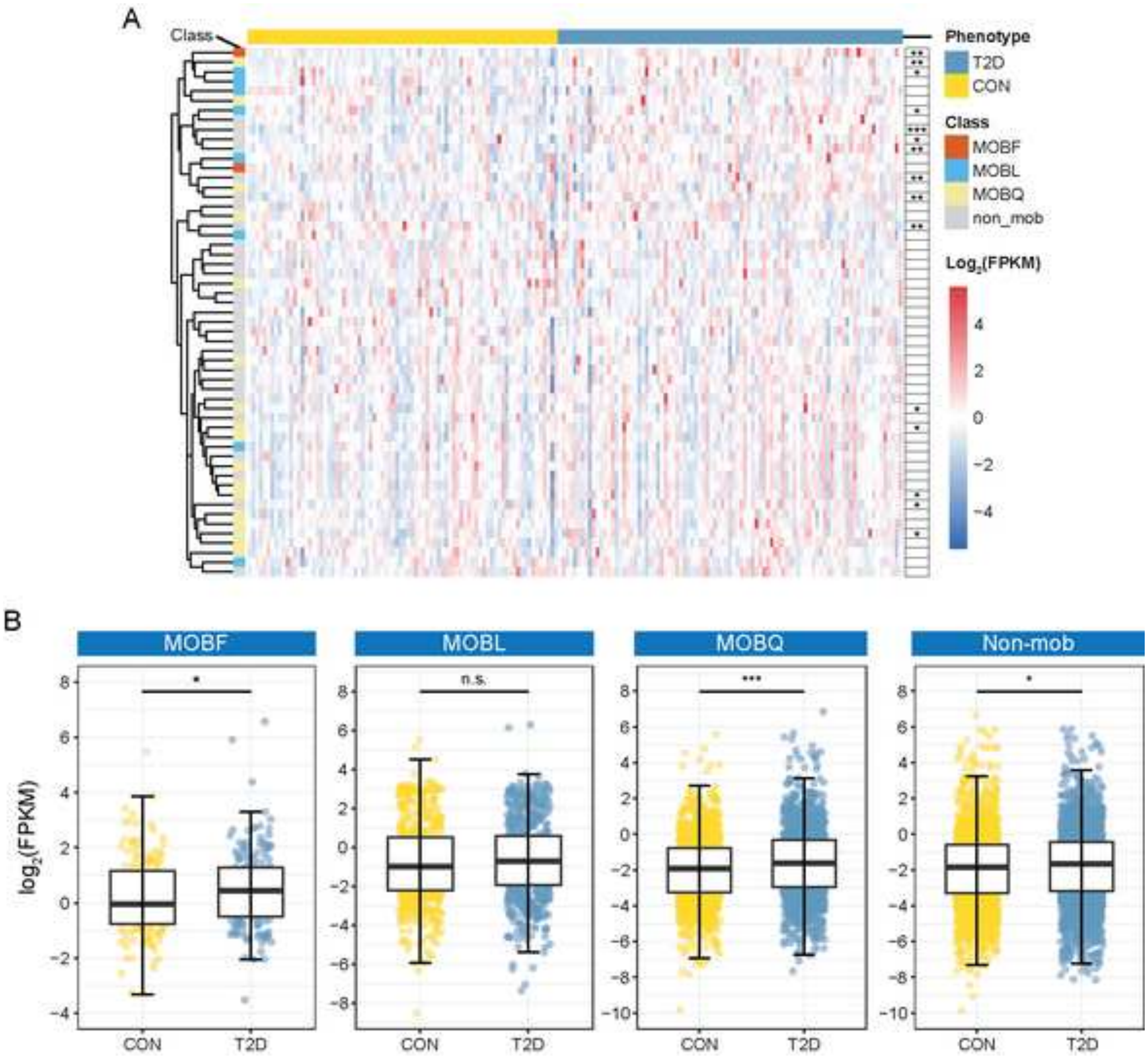

Figure 6

Click here to access/download;Figure;Figure6.tif

A

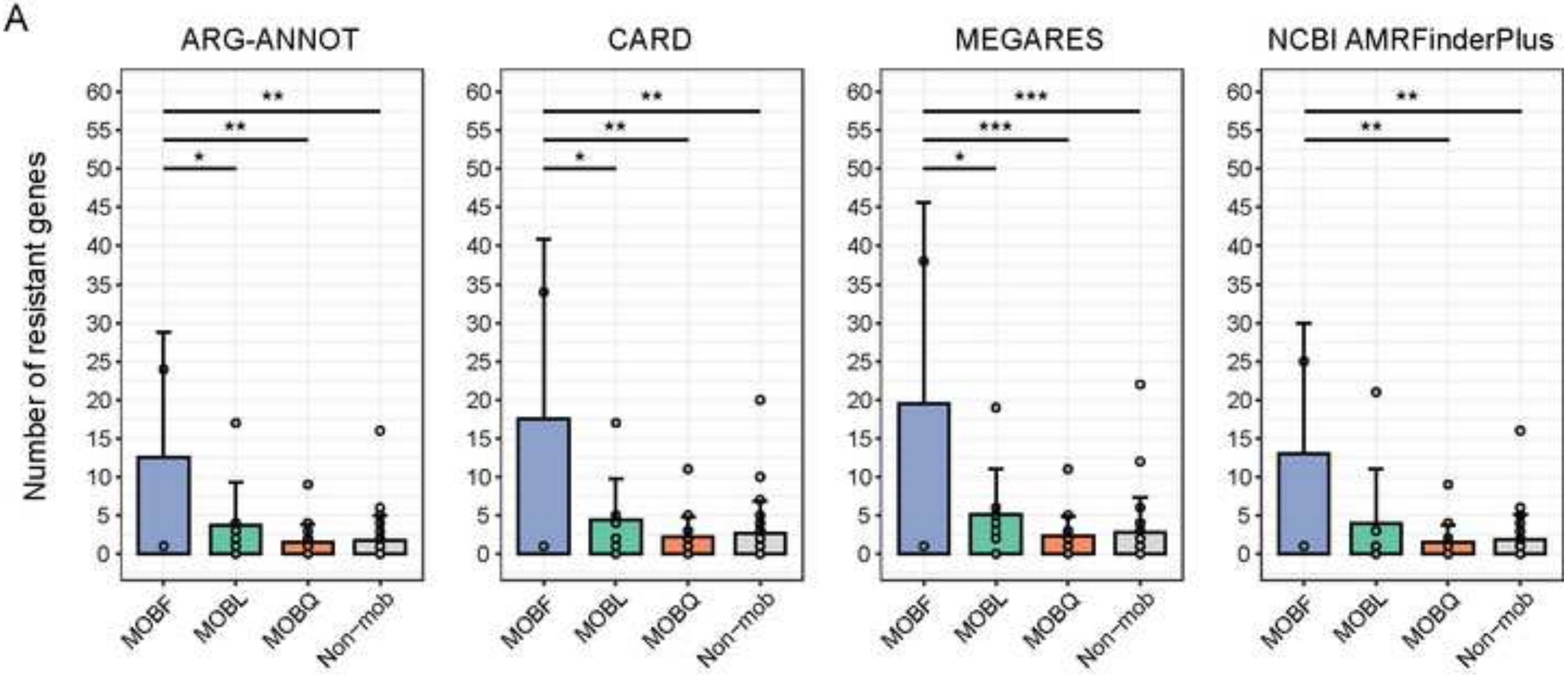

Supplementary Figures

Click here to access/download
Supplementary Material
Suplplementaty Figures.docx

Supplementary Table 1

Click here to access/download
Supplementary Material
Suplplementaty Table 1.xlsx

Supplementary Table 2

Click here to access/download
Supplementary Material
Suplplementaty Table 2.xlsx

Cover Letter

Dear Editor,

Thank you very much for you previous E-mail on May 16, 2024, regarding our manuscript, "MOBFinder: a tool for MOB typing for plasmid metagenomic fragments based on language model" (Manuscript Number: GIGA-D-24-00070). We are very pleased to know that our manuscript is potentially acceptable for publication in the journal, subject to the further revisions suggested by the reviewers. We are very grateful for your substantial and helpful advice with respect to our manuscript, and we are pleased to receive the reviewers' overall positive comments about our work. We thank the reviewers for their substantial and valuable comments, including their careful reading and checking of the manuscript, which greatly helped us improve the paper.

Our revisions and responses to the editor's and two reviewers' comments (italic text) are provided below.

To Editor:

1. One of the reviewers suggested you to improve the language, GigaScience is providing copy editing service, you can contact Qi Chen (chenqi@genomics.cn) if you need.

For this revision, we used the copy editing service of the *GigaScience* journal to refine the language. We thoroughly reviewed the entire text again to ensure that there are no serious errors in spelling, grammar, or meaning in each sentence. Specifically, based on the suggestions from the language editing, we have changed our title to "MOBFinder: A tool for mobilization typing of plasmid metagenomic fragments based on a language model".

2. In addition, please register any new software application in the bio.tools and SciCrunch.org databases to receive RRID (Research Resource Identification Initiative ID) and biotoolsID identifiers, and include these in your manuscript. Computational workflows should be registered in workflowhub.eu and the DOIs cited in the relevant places in the manuscript. These will facilitate tracking, reproducibility and re-use of your tool.

According to the journal requirement, we have registered the tool in the bio.tools and SciCrunch.org databases. In Lines 585-586 of the "Availability of Source Code and Requirements" section of the revised manuscript, we have added the following statement:

RRID: SCR_024451.

biotoolsID: MOBFinder.

Also, all the related scripts and data have also submitted to the GigaDB server.

To Reviewer #1:

Specific Comments:

1. the unpaired Wilcoxon signed-rank two-sided test.
-> should be corrected to either
"Wilcoxon rank-sum test" or "Mann-Whitney U test"

https://en.wikipedia.org/wiki/Mann%E2%80%93Whitney_U_test
"Wilcoxon rank-sum test" redirects here. For Wilcoxon signed-rank test, see Wilcoxon signed-rank test.
https://en.wikipedia.org/wiki/Wilcoxon_signed-rank_test
Not to be confused with Wilcoxon rank-sum test.

We apologize for the confusion between the Wilcoxon rank-sum test and the Wilcoxon signed-rank test. We have corrected this mistake in the revised manuscript. In Line 368 and 472 of the revised manuscript, the statistical method has been corrected to "the Wilcoxon rank-sum test".

2. Since MOBscan can only predict the MOB type with plasmid proteins, we annotated the plasmids in the test set with Prokka, then manually submitted them to the MOBscan website for MOB type annotation.

Given that MOBScan operates as an online tool and cannot be executed locally, the calculation of MOBScan's run time was confined to the duration spent on preprocessing with Prokka locally." (Please refer to Line 313-319 in the revised manuscript).

-> Actually, it can be executed locally using the scripts included in https://github.com/santirdnd/COPLA/. It may not be necessary to run MOBscan locally (it may be okay that they manually submitted them to the MOBscan website), but I'll

inform you regardless.

We are very grateful to Reviewer 1 for reminding us that MOBscan can be run locally. In the revised manuscript, we have removed the statement “MOBscan can only predict the MOB type with plasmid proteins” and revised the corresponding description to “We used the online server of MOBscan to perform the MOB annotation, and…” (Please refer to Line 312-313 in the revised manuscript).

3. Line 418-421
In the comparison, it was observed that MOBscan did not perform well, achieving low accuracy and kappa values across sequences of varying lengths, while MOB-suite exhibited marginally better performance than MOBscan when handling sequences of greater length (Figure 3A, 3B). (Please refer to Line 418-421 in the revised manuscript).

-> Do the authors' results contradict the following general expectation? MOB-typer utilizes BLAST, whereas MOBscan utilizes hmmscan, and therefore, MOBscan is expected to retrieve more distantly related proteins than MOB-typer.

We would like to thank Reviewer 1 for the discussion regarding BLAST and HMMscan. Firstly, we acknowledge that for more distantly related proteins, sequence searching based on HMM exhibits higher sensitivity than BLAST. However, we believe that our results do not contradict this theory. There are two reasons that might explain why, in this manuscript, the performance of tools based on HMM appears slightly inferior to those based on BLAST.

(1) The number of reference sequences can impact the performance of the tools. In MOB-suite, a large number of reference sequences are used for BLAST sequence alignment, whereas in MOBscan, the number of relaxase sequences used to profile HMM files for some MOB types is not very large. For instance, for the MOBF type, MOBscan utilizes 146 relaxase sequences for configuring HMM files, while MOB-suite employs 396 sequences to construct the BLAST database. The difference in the number of reference sequences could potentially lead to MOBscan’s performance being slightly inferior to that of MOB-suite.

(2) The aim of this study is not to design new methods for identifying novel relaxases. In our test data, the relaxases all come from sequenced plasmids, so there is some homology with the relaxases in the database. When the query sequence and the database sequence have high homology, the performance of BLAST may not necessarily be

worse than HMM. In fact, existing studies have shown that methods based on BLAST can sometimes outperform those based on HMM when the homology is high (Ref: PMID: 25140992).

4. MOB-suit and MOBscan are represented by blue lines, orange lines and gray lines respectively.
-> should be
"MOB-suite"

We thank Reviewer 1 for the careful checking of the manuscript. In Line 427-428 of the revised manuscript, we have revised "MOB-suit" to "MOB-suite".

5. I suggest receiving English language editing before publishing the paper.
"For the MOB typing, MOBscan [18] uses the HMMER model to annotated the relaxases and further perform MOB typing."
-> should be
"For the MOB typing, MOBscan [18] uses the HMMER model to annotate the relaxases and further perform MOB typing."

We are sorry for the grammatical error. In Line 109 of the revised manuscript, we have revised the sentence as "For the MOB typing, MOBscan [18] uses the HMMER model to annotate relaxase genes and classify plasmids accordingly".

In addition, we have used the copy editing service of the *GigaScience* journal to refine the language through the whole manuscript.

To Reviewer #2:

General Comments:

I would like to commend you on the revisions made to your manuscript following the initial round of reviews. It is evident that considerable effort has been put into addressing the concerns and suggestions raised during the first review. The changes and additions you have implemented have significantly enhanced the clarity, depth, and scholarly value of your paper. The manuscript has been improved substantially and all the initial concerns have been addressed satisfactorily. I support the publication of this manuscript in GigaScience.

Here, we would like to express our sincere gratitude to Reviewer 2 for the positive

comments on our work, describing our revised manuscript: "*The manuscript has been improved substantially and all the initial concerns have been addressed satisfactorily.*" We are very thankful for Reviewer 2's suggestions during the first revision process, which greatly enhanced the clarity, depth, and academic value of our paper.

In hoping that the above revision has clarified all the points by two reviewers and given a point-by-point response to all the concerns, we hereby submit our revised manuscript to the journal. We thank you for your kind consideration.

Note: The initial version of the article has been made public on bioRxiv (https://doi.org/10.1101/2023.12.06.570414), and beyond that, it has not been published in any other traditional journals.

Sincerely yours,

Zhencheng Fang, Ph.D.
Microbiome Medicine Center, Department of Laboratory Medicine, Zhujiang Hospital, Southern Medical University, Guangzhou, 510280, China
Email: fangzc@smu.edu.cn
