# Supplementary material for: MOBFinder: a tool for mobilization typing of plasmid metagenomic fragments based on a language model
Source: Gigascience. 2024 Aug 5;13:giae047. doi: 10.1093/gigascience/giae047 (PMC11299106; doi:10.1093/gigascience/giae047)
Supplement: giae047_Supplemental_Files [file giae047_supplemental_files.zip › Suplplementaty Figures.docx]

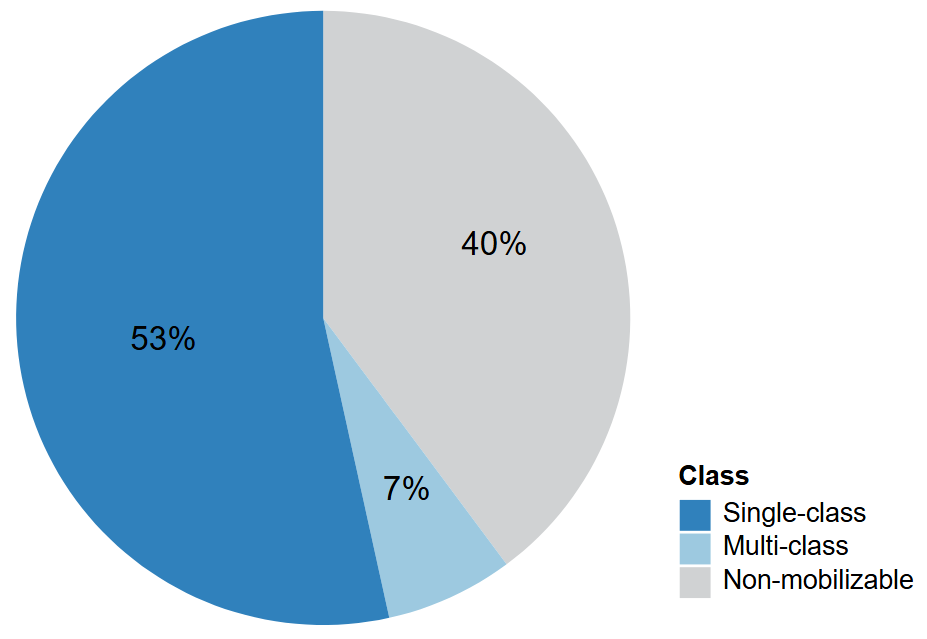


**Supplementary Figure 1**. MOB typing using MOB-suite. Single-class, plasmid genomes classified into one MOB type; multi-class, plasmid genomes classified into more than one MOB category; non-MOB, non-mobilizable plasmids.


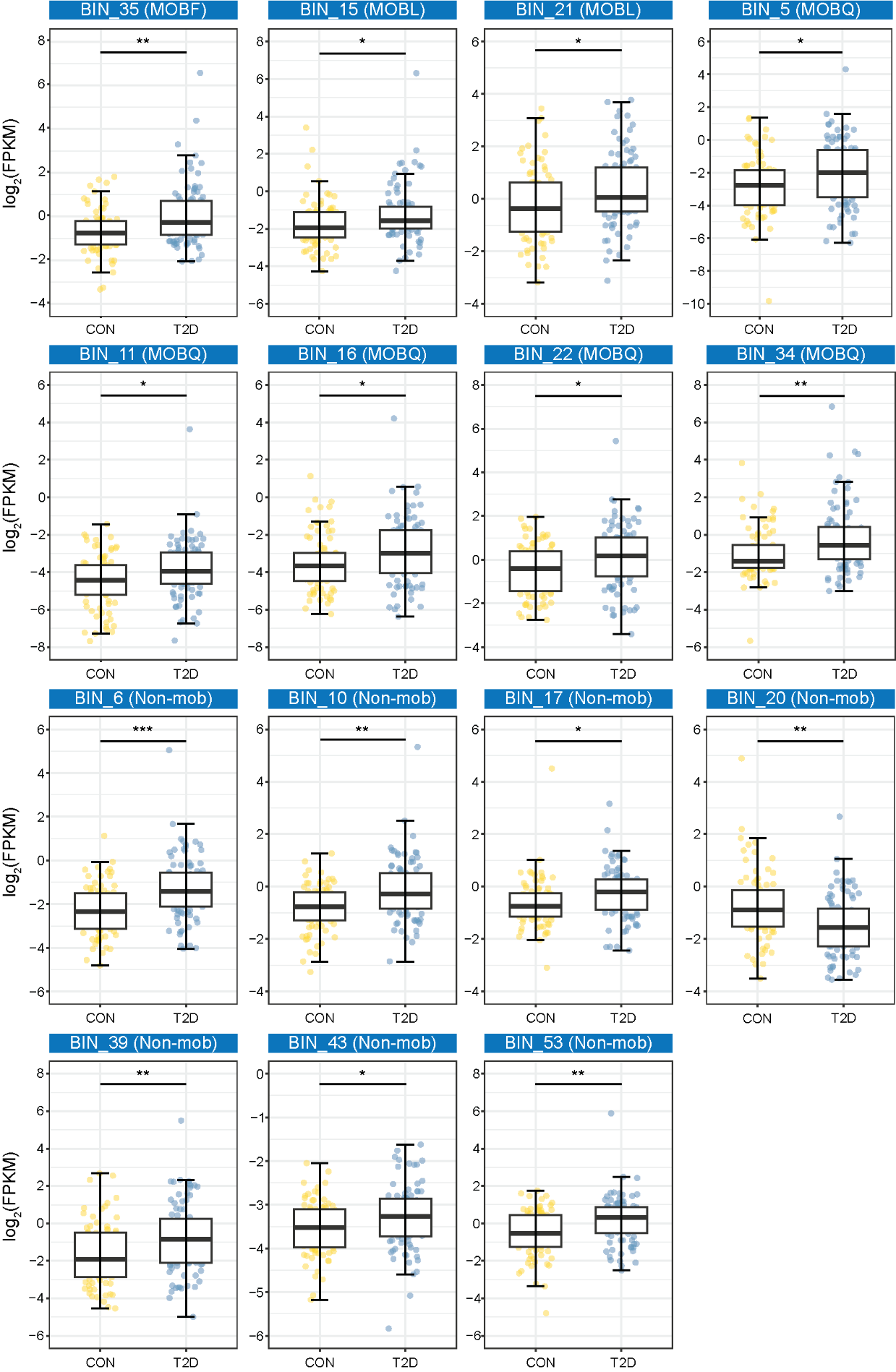


**Supplementary Figure 2.** Abundance of each significantly different plasmid bin from various MOB types between patients with type II diabetes and controls.
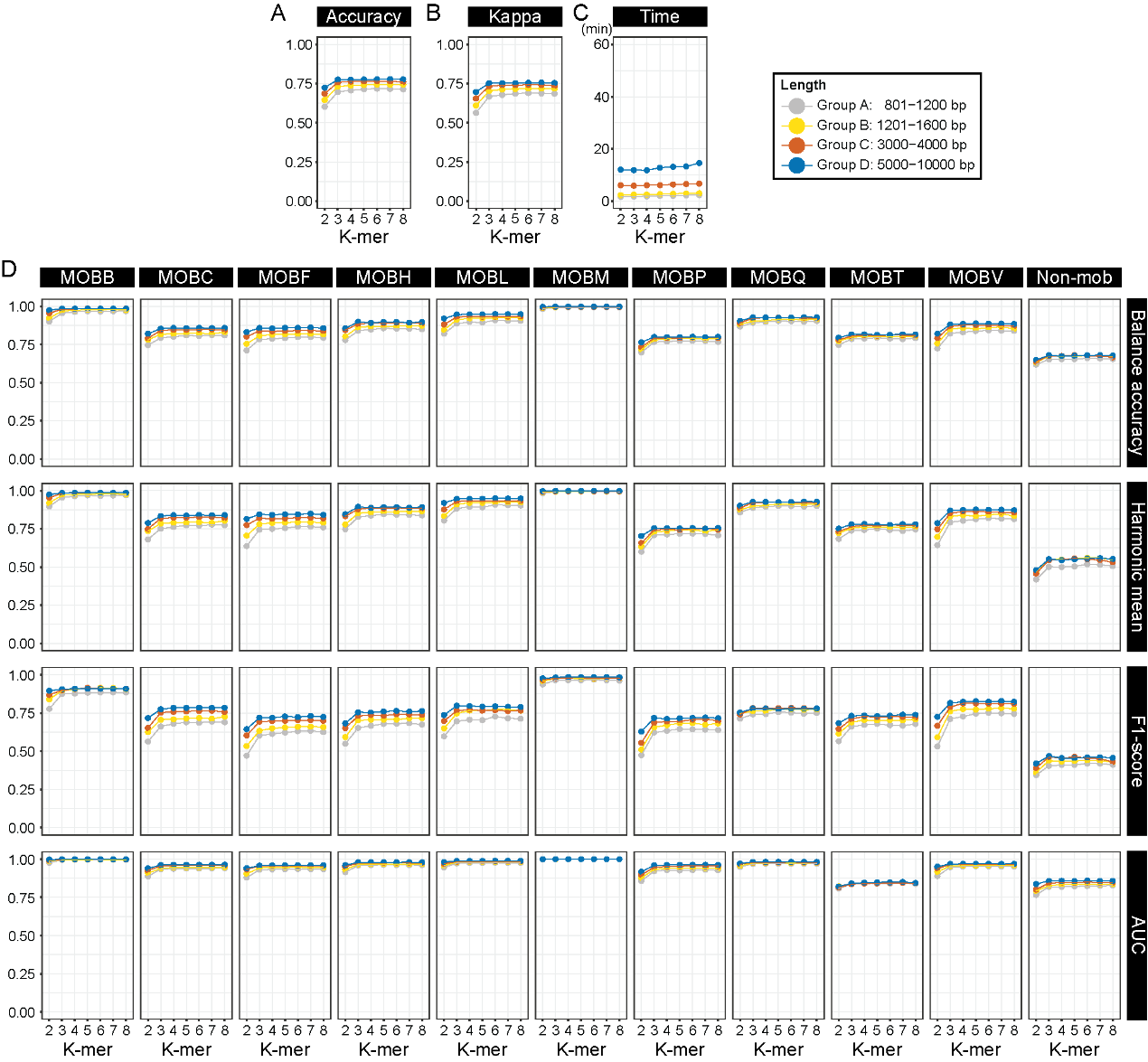


**Supplementary Figure 3.** Comparison results for the development of MOBFinder using word vectors trained with different *k*-mer lengths. (A-C) Overall *accuracy*, *kappa*, and *run time* of the MOB classification model trained with word vectors trained using different lengths of *k*-mers. (D) *Balanced accuracy*, *harmonic mean*, *F1-score*, and AUC of word vectors trained with different *k*-mer lengths across different MOB types.

Here, we trained word vectors for *k*-mers of varying lengths (*k* from 2 to 8). After completing the training, we encoded the benchmark dataset described in Section 2.4 using the word vectors of *k*-mers with different lengths and developed MOBFinder according to the method described in Section 2.5. Subsequently, we compared the outcomes of MOBFinder using word vectors trained with *k-*mers of various lengths, following the method detailed in Section 2.6.
