# Supplementary material for: MOBFinder: a tool for mobilization typing of plasmid metagenomic fragments based on a language model
Source: Gigascience. 2024 Aug 5;13:giae047. doi: 10.1093/gigascience/giae047 (PMC11299106; doi:10.1093/gigascience/giae047)
Supplement: giae047_Response_to_Reviewer_Comments_Original_Submission [file giae047_response_to_reviewer_comments_original_submission.pdf]

Author's Response To Reviewer Comments

The response to specific reviewer and editor comments has been uploaded in the cover letter.
