# Supplementary material for: MOBFinder: a tool for mobilization typing of plasmid metagenomic fragments based on a language model
Source: Gigascience. 2024 Aug 5;13:giae047. doi: 10.1093/gigascience/giae047 (PMC11299106; doi:10.1093/gigascience/giae047)
Supplement: giae047_Reviewer_1_Report_Original_Submission — Haruo Suzuki -- 3/23/2024 Reviewed [file giae047_reviewer_1_report_original_submission.pdf]

Reviewer Report

Title: MOBFinder: A tool for mobilization typing for plasmid metagenomic fragments based on a language model

Version: Original Submission **Date:** 3/23/2024

Reviewer name: Haruo Suzuki

Reviewer Comments to Author:

I recommend that the authors consider revising based on the following points.#)the unpaired Wilcoxon signed-rank two-sided test.-> should be corrected to either"Wilcoxon rank-sum test" or "Mann-Whitney U test"https://en.wikipedia.org/wiki/Mann%E2%80%93Whitney_U_test"Wilcoxon rank-sum test" redirects here. For Wilcoxon signed-rank test, see Wilcoxon signed-rank test.https://en.wikipedia.org/wiki/Wilcoxon_signed-rank_testNot to be confused with Wilcoxon rank-sum test.#)Since MOBscan can only predict the MOB type with plasmid proteins, we annotated the plasmids in the test set with Prokka, then manually submitted them to the MOBscan website for MOB type annotation.Given that MOBScan operates as an online tool and cannot be executed locally, the calculation of MOBScan's run time was confined to the duration spent on preprocessing with Prokka locally." (Please refer to Line 313-319 in the revised manuscript).-> Actually, it can be executed locally using the scripts included in https://github.com/santirdnd/COPLA/. It may not be necessary to run MOBscan locally (it may be okay that they manually submitted them to the MOBscan website), but I'll inform you regardless.#)In the comparison, it was observed that MOBscan did not perform well, achieving low accuracy and kappa values across sequences of varying lengths, while MOB-suite exhibited marginally better performance than MOBscan when handling sequences of greater length (Figure 3A, 3B). (Please refer to Line 418-421 in the revised manuscript).-> Do the authors' results contradict the following general expectation? MOB-typer utilizes BLAST, whereas MOBscan utilizes hmmscan, and therefore, MOBscan is expected to retrieve more distantly related proteins than MOB-typer.#)MOB-suit and MOBscan are represented by blue lines, orange lines and gray lines respectively.-> should be"MOB-suite"#) I suggest receiving English language editing before publishing the paper."For the MOB typing, MOBscan [18] uses the HMMER model to annotated the relaxases and further perform MOB typing."-> should be"For the MOB typing, MOBscan [18] uses the HMMER model to annotate the relaxases and further perform MOB typing."

Level of Interest

Please indicate how interesting you found the manuscript: Choose an item.

Quality of Written English

Please indicate the quality of language in the manuscript: Choose an item.

Declaration of Competing Interests

Please complete a declaration of competing interests, considering the following questions:

- Have you in the past five years received reimbursements, fees, funding, or salary from an organisation that may in any way gain or lose financially from the publication of this manuscript, either now or in the future?
- Do you hold any stocks or shares in an organisation that may in any way gain or lose financially from the publication of this manuscript, either now or in the future?
- Do you hold or are you currently applying for any patents relating to the content of the manuscript?
- Have you received reimbursements, fees, funding, or salary from an organization that holds or has applied for patents relating to the content of the manuscript?
- Do you have any other financial competing interests?
- Do you have any non-financial competing interests in relation to this paper?

If you can answer no to all of the above, write 'I declare that I have no competing interests' below. If your reply is yes to any, please give details below.

I declare that I have no competing interests

I agree to the open peer review policy of the journal. I understand that my name will be included on my report to the authors and, if the manuscript is accepted for publication, my named report including any attachments I upload will be posted on the website along with the authors' responses. I agree for my report to be made available under an Open Access Creative Commons CC-BY license (http://creativecommons.org/licenses/by/4.0/). I understand that any comments which I do not wish to be included in my named report can be included as confidential comments to the editors, which will not be published.

Choose an item.

To further support our reviewers, we have joined with Publons, where you can gain additional credit to further highlight your hard work (see: https://publons.com/journal/530/gigascience). On publication of this paper, your review will be automatically added to Publons, you can then choose whether or not to claim your Publons credit. I understand this statement.

Yes Choose an item.
